# Functional Screening of Antibiotic Resistance Genes from a Representative Metagenomic Library of Food Fermenting Microbiota

**DOI:** 10.1155/2014/290967

**Published:** 2014-08-28

**Authors:** Chiara Devirgiliis, Paola Zinno, Mariarita Stirpe, Simona Barile, Giuditta Perozzi

**Affiliations:** ^1^CRA-NUT, Food & Nutrition Research Center, Agricultural Research Council, Via Ardeatina 546, 00178 Rome, Italy; ^2^Department of Biology and Biotechnology Charles Darwin, Sapienza University of Rome, Piazzale Aldo Moro 5, 00185 Rome, Italy

## Abstract

Lactic acid bacteria (LAB) represent the predominant microbiota in fermented foods. Foodborne LAB have received increasing attention as potential reservoir of antibiotic resistance (AR) determinants, which may be horizontally transferred to opportunistic pathogens. We have previously reported isolation of AR LAB from the raw ingredients of a fermented cheese, while AR genes could be detected in the final, marketed product only by PCR amplification, thus pointing at the need for more sensitive microbial isolation techniques. We turned therefore to construction of a metagenomic library containing microbial DNA extracted directly from the food matrix. To maximize yield and purity and to ensure that genomic complexity of the library was representative of the original bacterial population, we defined a suitable protocol for total DNA extraction from cheese which can also be applied to other lipid-rich foods. Functional library screening on different antibiotics allowed recovery of ampicillin and kanamycin resistant clones originating from *Streptococcus salivarius* subsp. *thermophilus* and *Lactobacillus helveticus* genomes. We report molecular characterization of the cloned inserts, which were fully sequenced and shown to confer AR phenotype to recipient bacteria. We also show that metagenomics can be applied to food microbiota to identify underrepresented species carrying specific genes of interest.

## 1. Introduction

Bacterial fermentation products provide specific sensory properties which characterize a wide variety of foods. Foodborne fermenting microorganisms can either be added to sterilized matrices as commercial starter mixtures composed of specific strains [1] or they can originate from the environment as in the case of the raw ingredients employed for artisanal food production. This latter condition is the most frequent in traditional cheese manufacturing, which does not employ selected industrial starters as it relies on the microflora naturally present in raw material, often represented by complex microbial consortia whose species profile reflects local microenvironments. Lactic acid bacteria (LAB) are prevalent microorganisms within the fermenting food microbiota. Complex environmental bacterial communities have been extremely difficult to characterize, mostly due to the limitations imposed by culture-dependent approaches [2]. The proportion of bacteria from natural environments that are not readily culturable was estimated to about 99% [3]. Therefore, the majority of environmental strains have never been described and cannot be exploited for research and for biotechnological applications. Metagenomics represents, at the moment, the most promising culture-independent, DNA-based molecular method to overcome such difficulties [4, 5]. Food microbiology has taken advantage of the application of such innovative strategies, which were applied to study the composition and the evolution, as well as the spatial distribution of fermenting microbial ecosystems [6, 7].

Metagenomic libraries can be constructed from a variety of sources and through several methods, depending on the objective to be pursued. Taxonomic analysis requires comparison of conserved genome stretches, and therefore total DNA extracted from environmental microbiota is mostly PCR-amplified prior to cloning into the appropriate vectors, resulting in gene-specific metagenomic libraries (most frequently 16S-ribosomal DNA libraries) that are easily analyzed using bacterial genome databases and tools. However, the PCR step introduces a bias in DNA complexity, by altering the relative species proportions with respect to their relative abundance within the original microbiota. On the other hand, direct cloning of total DNA extracted from complex microbial communities, although quantitatively more reliable, requires very high cloning efficiencies to avoid selection against the least represented genomes. The choice of methodological approach is therefore strictly dependent on the purpose of the study, although the majority of metagenomic libraries described in the literature employ PCR-amplified DNA as starting material.

Our laboratory has focused on studying antibiotic resistance (AR) genes from microbial food sources and their corresponding genomic context which represents the main driver of horizontal transfer to human opportunistic pathogens [8, 9]. AR genes are widely distributed in several different environments, including food production systems [10]. Recent findings suggest the possibility of horizontal gene transfer among bacteria within food matrices, since fermented and minimally processed foods contain high titers of live microbial cells [11]. We have chosen a specific water buffalo fermented cheese as a model; that is, Mozzarella di Bufala Campana (MBC), which is produced in restricted geographical regions of Italy, is consumed fresh and therefore supplies high titers of live bacteria [12, 13]. Fermentation in this product is performed by specific thermophilic microbial communities provided by natural whey starter cultures (NWSC) [14], which, together with the microbiota of raw milk, contribute a wide variety of uncharacterized, environmental strains to the final cheese ready for consumption. Although PCR amplification with gene-specific primers of total DNA extracted from MBC had shown the presence of several AR genes, when applying culture-dependent approaches to isolate the corresponding AR strains, we were able to identify AR colonies only from the raw materials employed for cheese production, in which microbial titers are higher than in the final product [13]. Previous studies by other laboratories demonstrated the efficacy of culture-independent approaches in the identification of AR clones from oral metagenome libraries [15]. We turned therefore to metagenomics, with the aim of constructing a representative library of the entire cheese microbiome that could allow detection and analysis of AR genes carried by nonculturable or underrepresented species within the microbiota of fermented food products. Our experimental design involved construction of a fosmid metagenomic library containing large fragments of total DNA extracted from MBC, followed by functional screening of recombinant clones on representative antibiotics belonging to different pharmacological classes and employed in the past in animal farming and/or presently used in human therapy, namely, ampicillin, kanamycin, gentamycin, and tetracycline. To best reflect the complexity of the fermenting microbiota, the metagenomic library needed to be quantitatively representative of the different species present in the starting material, and we thus had to confront with several technical aspects representing crucial steps towards our goal. We describe in this paper the choices deriving from such a challenge, which resulted in the construction and screening of a cheese metagenomic library leading to the identification of fosmidborne, LAB derived genes expressing an AR phenotype in the* E. coli* host. To the best of our knowledge, this is the first report of direct, nonamplified metagenomic cloning of microbial genes from a complex fermented food matrix.

## 2. Materials and Methods

### 2.1. Mozzarella Processing and Sampling

Samples of MBC were received on the day of production from four dairy factories located in different provinces of central and southern Italy (Latina-LT; Salerno-SA; Caserta-CE; Foggia-FG). We exclusively selected dairy plants with associated animal farming, which guarantees reproducible sources of milk and associated microbiota profiles for cheese production. Samples were stored at 4°C and processed within 12 h. Pooled or single samples of MBC were homogenized with a BagMixer400 (Interscience, France) in sodium citrate solution (2% w/v) at a concentration of 0.5 g/mL. In order to test the titer of mesophilic cultivable LAB, serial dilutions were made in Quarter Strength Ringer's solution and plated on MRS agar medium (Oxoid Ltd, Basingstoke, Hampshire, England), as previously reported [13]. Plates were incubated at 30°C for 48 h, under aerobic and anaerobic conditions (Anaerocult A, Merck, Germany).

### 2.2. DNA Extraction

Total DNA extraction from MBC was performed by a modified version of a published method [16]. Relevant methodological modifications are described in Section 3. Microscopic observation of sample aliquots during the lysis procedure was carried out to monitor the progressive disappearance of intact microbial cells. The yield of total DNA obtained from MBC samples was about 0.5 *μ*g/g.

### 2.3. Library Construction

Metagenomic library construction was performed using the EpiFos Library Production Kit (Epicentre Technologies, Madison, Wisconsin, USA), following manufacturer's indications with the following modifications: ligation reaction was carried out with ligase enzyme from Stratagene, and incubation time of* E. coli* host cells with phage particles during the infection process was extended to 40 min. Such modifications resulted in increased packaging efficiency as well as in improved titer of packaged fosmid clones by about 4-fold.

### 2.4. Antibiotics and Reagents

Antibiotics (ampicillin, chloramphenicol, erythromycin, gentamycin, kanamycin, tetracycline, and vancomycin) were purchased from Sigma (Italy). Restriction enzymes were provided by Takara (Italy). PCR reagents were obtained from Invitrogen (Italy).

### 2.5. Bacterial Strains and Growth Conditions

*E. coli* EPI100-T1^R^ [F^−^ mcrA Δ(*mrr-hsd*RMS-*mcr*BC) *ϕ*80d*lac*ZΔM15 Δ*lac*X74* rec*A1* end*A1* ara*D139 Δ (*ara*,* leu*)7697* gal*U* gal*K *λ* ^−^ *rps*L* nup*G* ton*A] was grown in LB medium (Difco) overnight at 37°C with shaking. Recombinant libraries were stored at −80°C in LB-Cm (LB medium added with chloramphenicol at a final concentration of 12.5 mg/L) containing glycerol (15% v/v). For screening purposes, libraries were plated on LB-Cm agar plates and a total of 20.000 recombinant* E. coli* clones were picked and stored in 96-multiwell plates containing 10 clones/well. This plates were then replica-plated on LB-agar added with the appropriate antibiotic, with the aid of a metallic replica plater for 96-multiwell (Sigma, Italy), and grown overnight at 37°C.

### 2.6. DNA Amplification and Molecular Analysis

Microbial DNA was amplified by PCR as previously described [9]. Fosmid DNA was isolated with FosmidPrep kit (Epicentre Technologies, Madison, Wisconsin, USA) according to manufacturer's instructions. Primers used are listed in Table 1. Restriction analysis and southern hybridization were performed by standard protocols, using probes labelled with digoxigenin-11-dUTP (Roche Diagnostics, Milan, Italy).

The two-step gene walking method consisted of a walking-PCR (step 1) followed by direct sequencing of the PCR product (step 2) [17]. Walking-PCRs were performed as described [18], with the specific primer Epifos-FW (Epicentre). PCR products were purified using a NucleoSpin Extract II kit according to the manufacturer's instructions and sequenced with T7 primer.

### 2.7. Full Sequencing of Recombinant AR Fosmids

Sequencing was performed at the DNA sequencing facility of GenProbio s.r.l., Italy (http://www.genprobio.com/).

## 3. Results and Discussion

### 3.1. Microbial Representativeness for Metagenomic Analysis

The first step towards library construction concerned sampling of MBC from different sources to ensure metagenome representativeness of the entire microbiota that characterizes this specific food product. To this aim, MBC cheeses were collected from four different dairy plants located in Italian provinces where the majority of producers are present (see Section 2). Some of the selected geographical areas are over 300 Km apart from each other and represent different pedoclimatic microenvironments leading to diverse milk microbial profiles [13]. We reasoned that pooling these samples should lead us to obtain genomic DNA representing the great majority of microbial genera/species entering the human GI tract through MBC consumption. Moreover, the titer of the mesophilic LAB component of the MBC microbiota resulted in about 10^6^ Cfu/g (data not shown), in accordance with our previous findings [13]. The four MBC samples were therefore pooled in equal proportions and microbial DNA for library construction was extracted from the resulting homogenate.

### 3.2. Food-Derived DNA as a Source of Bacterial Genomes

A strategic aspect that we had to confront with in order to achieve representative, nonamplified metagenomic DNA of good quality was the optimization of qualitative/quantitative steps in the DNA extraction procedure. Fat represents a major component in dairy products, and its presence can impair bacterial recovery and lysis, which in turn greatly affects DNA yields. In order to obtain high molecular weight genomic DNA required for fosmid library construction, we therefore modified a previously published protocol [16], improving fat removal and DNA extraction efficiencies by introducing serial washes of dairy homogenates in Na-citrate buffer, followed by a combination of freeze-thaw cycles and mechanical as well as enzymatic lysis.

**Figure 1:**
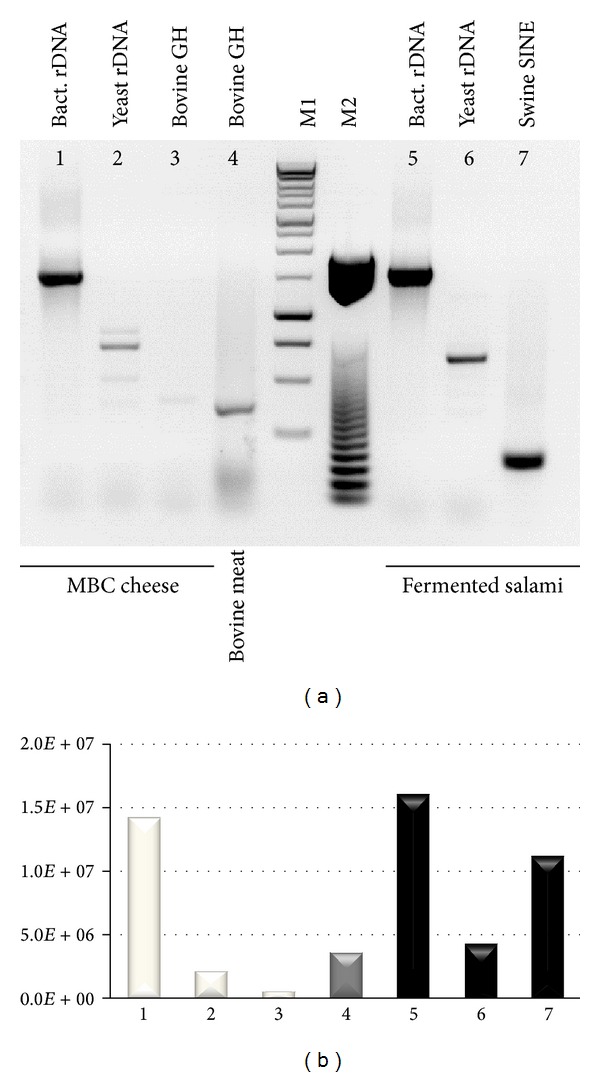
Total DNA extracted from dairy products contains almost exclusively microbial DNA with undetectable contamination from higher eukaryotic cell DNA. (a) PCR amplifications of DNA extracted from cheese (left) or meat (right) matrices using the species-specific primers listed in Table 1. M1: 1 Kb DNA ladder. M2: 50 bp DNA ladder. (b) Amplicon quantification obtained with the freely available ImageJ densitometry software [45]. Numbers indicate the corresponding PCR amplicons in (a).

The presence of contaminating DNA from eukaryotic cells is another crucial aspect affecting representativeness of microbial genomes within the library, which is usually overcome by PCR amplification. Unlike meat fermentation products, dairy foods should contain almost exclusively microbial DNA, with very low contamination from higher eukaryotic cell DNA [8], but this aspect needed to be assayed before proceeding with our approach of direct cloning unselected high molecular weight DNA extracted from food. To this aim, a PCR approach was carried out including DNA extracted from fermented swine meat sausages for comparison, with primers specific for either microbial or eukaryotic species-specific genes, namely, bacterial 16S rDNA, yeast 5.8S rDNA, buffalo growth hormone gene, and swine SINE (short interspersed nuclear element). Primer sequences are reported in Table 1. The results shown in Figure 1 confirm that total DNA extracted from MBC is almost exclusively of microbial origin. Bacterial DNA represented the major component, while yeast DNA accounted for about 10% of the amplicons (Figure 1(b)). On the other hand, eukaryotic DNA was almost undetectable in MBC samples, while representing a great proportion of the total DNA extracted from fermented sausages. These results unequivocally show that microbial genomes constitute the great majority of unamplified DNA extracted from a dairy food matrix such as MBC, which could then be used directly for metagenomic library construction.

### 3.3. MBC Metagenomic Library

The fosmid vector that we chose for library construction is suitable for cloning genomic inserts of approximately 40 kilobases in size. This feature allows us to characterize also the genomic context surrounding specific genes, thus increasing the chances of identifying the bacterial species of origin through sequencing of flanking regions. In the case of AR genes, analysis of the genomic context can also reveal association with mobile elements, indicative of a potentiality for horizontal inter- and intraspecies transfer [19]. The EpiFOS vector was also chosen because it utilizes a novel strategy for cloning randomly sheared, end-repaired DNA, leading to generation of highly random DNA fragments, in contrast to DNA fragmentation by partial restriction digestion that leads to more biased libraries. Fosmid clones containing high molecular weight fragments ranging between 35 and 45 Kb were used to infect the recipient* E. coli* EPI-100T1^R^ strain, resulting in a 4 × 10^6^ CFU/mL library titre. We estimated the minimum required representativeness of the library using the formula *N* = ln⁡(1 − *P*)/ln⁡(1 − *f*), where *P* is the desired probability (expressed as a fraction) of a given sequence being present in the library, *f* is the proportion of the metagenome within a single clone, and *N* is the number of clones required. Metagenomic samples introduce additional constraints, due to the unpredictable number of different species/strains that constitute the original microbiota; thus, only a rough estimate can be derived on the relative abundance of different populations within the complex bacterial community. For example, assuming an average genome size of 4 Mb, a library with 40 kb average inserts would require at least 100 clones to provide coverage of the entire genome, provided all clone inserts contain distinct sequences. If the genome of this reference organism represents about 10% of the total metagenome, screening 1.000 clones would likely provide a reasonable chance of detecting a specific sequence of interest. Basing on these calculations and considering an average fragment length of 25–30 Kbp, we estimated a total number of 20.000 clones to account for a well-represented MBC fermenting microbiome, as the overall size encompasses 1 Gbp which corresponds to approximately 250 times the size of the* E. coli* genome (4 × 10^6^ bp).

**Figure 2:**
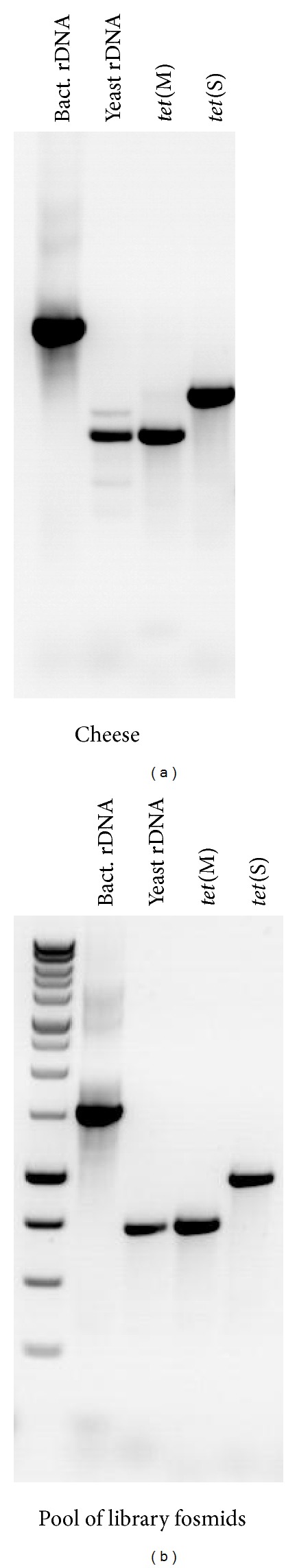
Fosmid cloning of total MBC DNA does not alter complexity PCR amplification of total DNA extracted from pooled MBC samples (a) or from pooled recombinant fosmids following metagenomic library construction (b). Primer pairs: bacterial rDNA, yeast rDNA,* tet*(M), and* tet*(S) (Table 1). M: 1 Kb DNA ladder.

To ensure that the library reflected the original DNA complexity, total DNA extracted from the pooled MBC samples was compared to pooled library DNA through PCR amplification of bacterial 16S rDNA and yeast 18S-28S intergenic sequences. The results in Figure 2 show that the DNA was qualitatively similar before and after library construction, thus proving that our cloning strategy can preserve the DNA complexity of foodborne microbial genomes.

Moreover, sequence analysis of randomly selected clone inserts followed by sequence similarity searches in public genome databases (Blast, http://blast.ncbi.nlm.nih.gov/Blast.cgi) confirmed the presence of both bacterial and yeast genomes in the original proportions within the MBC metagenomic library (data not shown). As a control for the presence of specific AR genes, we also confirmed that* tet*(M) and* tet*(S), which are among the best characterized tetracycline resistance determinants in LAB, are well represented in both total MBC DNA and library clones.

### 3.4. Functional Screening for Antibiotic Resistance Genes

Functional metagenomics requires heterologous expression of exogenous genes, coupled with activity-based assays that can be easily performed on plates to select specific protein functions. This approach is more efficient than other two-step molecular methods based on detection of specific gene sequences and subsequent demonstration of their functionality, but it can be hampered by potential incompatibility between donor and host expression machineries [20–22]. The emergence and spread of antibiotic resistance determinants in the fermenting microbiota from different foods has been increasingly reported and reviewed by several groups worldwide, including ours [23–25], pointing at the need for deeper understanding of the mechanisms for horizontal transfer of AR genes, which are still partially unknown. AR genes can be easily selected on antibiotic containing media and were therefore chosen in this work to test the efficiency of recovery of LAB genes, which are the most represented species in the MBC microbiome under study. Moreover, AR genes for some of the most common antibiotics are not as well characterized in Gram-positives as they are in Gram-negative pathogens, and functional screening could therefore lead to the possible identification of novel proteins conferring AR in LAB. We therefore sought to test the MBC metagenomic library through functional screening with antibiotics belonging to five different pharmacological classes (tetracycline, aminoglycosides, beta-lactams, macrolides, and glycopeptides), which were chosen on the basis of their relevance in animal and human therapy and/or due to their widespread use in the past as growth promoters. Tetracyclines have been widely used in livestock farming and several tetR determinants were later identified in foodborne LAB from different fermented food sources [8, 26–28]. Along with tetracycline, the macrolide antibiotic erythromycin has also been intensively used in the past as growth promoter, and erythromycin-resistance genes represent, together with the TetR genes, the most widespread resistance determinants in foodborne bacteria [8, 27, 29]. Aminoglycosides and beta-lactams, on the other hand, have never been used as growth promoters, but they represent clinically relevant antibiotics whose corresponding resistance genes have also been described in foodborne LAB strains (lactobacilli and lactococci) [23].

As a first step towards functional screening for AR clones within the MBC library and to avoid interference from AR potentially present in the* E. coli* host, minimum inhibitory concentrations (MIC) were determined for the* E. coli* Epi100T1^R^ strain on each antibiotic to be tested. Streptomycin was not considered as the corresponding resistance gene* rpsL* is known to be carried by the Epi100T1^R^ strain. The resulting MIC values are reported in Table 2, showing that the* E. coli* host strain is phenotypically resistant to erythromycin and vancomycin, while displaying susceptibility to tetracycline, kanamycin, gentamycin, and ampicillin, with MIC values of 5, 25, 12, and 25 mg/L, respectively (Table 2). These latter four antibiotics were therefore chosen for functional screening of the MBC library at concentrations corresponding to their respective MIC values for* E. coli*. As positive control, clones were replicated on LB agar containing chloramphenicol, whose resistance determinant represents a selective marker (chloramphenicol acetyl transferase) encoded by the fosmid vector. Functional screening by replica plating of 20.000 independent library clones on antibiotic containing plates led to the selection of 4 TetR, 2 KanR, and 6 AmpR colonies. No colonies were rescued on gentamycin containing plates.

To confirm that phenotypic resistance in the surviving colonies was conferred by resistance determinants encoded by cloned inserts, fosmid DNA was extracted from each AR clone, packaged into phage particles, and used to infect the* E. coli* host Epi100T1^R^. Secondary screening of the resulting clones was performed on LB agar plates containing the appropriate antibiotic. All kanamycin and ampicillin resistant bacteria confirmed their ability to grow on the corresponding antibiotic-containing medium following this secondary screening (Table 2). Unexpectedly, the tetracycline resistant colonies identified by primary screening resulted in false positives. A possible explanation is that they arose by spontaneous mutations in the* E. coli* genome induced by the mutagenic effect of chloramphenicol [30]. Unlike kanamycin and ampicillin resistant clones, TetR colonies had indeed been selected on plates containing both antibiotics (tetracycline and chloramphenicol) in the growth medium to increase the selective pressure. Several antibiotics, among which chloramphenicol, are known to induce mutagenesis and recombination within bacterial genomes, which may facilitate bacterial adaptation to different types of stress, including antibiotic pressure [30]. For this reason chloramphenicol was excluded from screening plates used for selection of ampicillin and kanamycin resistant colonies.

### 3.5. Molecular Characterization of AR Recombinant Clones

**Figure 3:**
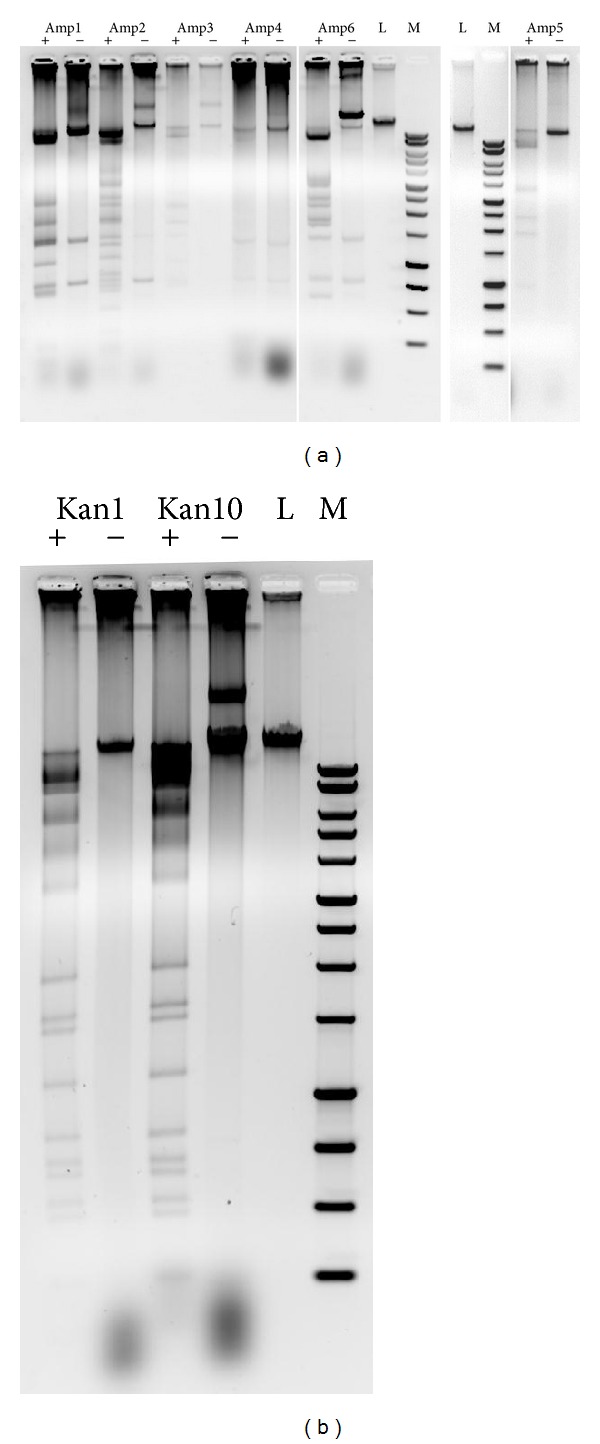
Restriction analysis of AR recombinant fosmids. Fosmid DNA extracted from each clone was digested (+) or not (−) with Hind III and fractionated by agarose gel electrophoresis. (a) AmpR clones. (b) KanR clones. L: undigested phage lambda DNA. M: 1 Kb DNA ladder.

To further characterize the genomic features of the AR clones, fosmid DNA was extracted from both AmpR (clones Amp1-6) and KanR (clones Kan1 and 10) colonies and subjected to restriction analysis with the HindIII endonuclease, which cuts the pEpiFOS-5 vector at a unique site. The results are reported in Figures 3(a) and 3(b) for the AmpR and KanR clones, respectively. With the exception of clone Amp4, which remained undigested by HindIII, the remaining AmpR clones displayed different restriction patterns with almost no overlapping bands, suggesting that the cloned inserts likely originate from distinct genomes within the metagenomic DNA. On the other hand, restriction of the two kanamycin resistant clones yielded fully overlapping restriction bands, indicating identity of the inserts. We therefore considered them as a single resistant clone in our subsequent analysis.

The presence of the AR gene within a large genomic fragment allows species identification even before the full sequence of the cloned fragment is obtained. Preliminary analysis in this direction, performed by two-step gene walking [17], led to associate* Streptococcus salivarius* subsp.* thermophilus* genomic sequences to clones Amp1, 2, 3, and 6 and* Lactobacillus helveticus* genomic sequences to clones Amp4 and 5 (data not shown).

Full sequencing of the clone inserts, performed for 3 AmpR clones and for the KanR clone, confirmed species identification.* S. thermophilus* is expected to be a very abundant species in MBC, especially within the first few days of cheese production, as the last processing step for this specific product includes heating at 95°C for a few minutes. The full sequences, whose deposition in public databases is in progress, are provided as supplementary data (See Supplementary Material available online at http://dx.doi.org/10.1155/2014/290967), while the most relevant features for each clone are summarized in Table 3. Fragment size in the four sequenced clones ranged between 14 and 38 Kbp with correspondingly increasing number of predicted ORFs (14–43). Sequence analysis revealed the presence of two genes encoding penicillin-binding proteins (PBP) in clone Amp3 and of RNA methyltransferase genes in clones Amp3 and Kan10. Synthesis of low-affinity PBPs represents an important mechanism of resistance in some Gram-positive bacteria. Several PBPs have been described in resistant strains, including PBP2a from methicillin resistant* Staphylococcus aureus* (MRSA), PBP2x from penicillin resistant* Streptococcus pneumoniae*, and PBP5fm from drug resistant* Enterococcus faecium* [31, 32]. Blast similarity searches revealed that ORFs PBP1A and PBP2A from clone Amp3 were homologous to* S. thermophilus* penicillin-binding proteins. Two distinct ORFs encode rRNA methyltransferases in clones Amp3 and Kan10, and only the gene present in the Kan10 clone can be specifically identified as 23S rRNA methyltransferase on the basis of sequence similarity searches, while the Amp3 clone cannot be specifically attributed to 16S or 23S. Ribosomal RNA methylation is a frequent mechanism for macrolide and aminoglycoside resistance. RNA methyltransferases were shown to specifically target 16S rRNA in the case of resistance to aminoglycosides such as kanamycin [33]. However, the 23S rRNA methyltransferase encoded by* Cfr* gene of* S. aureus* and* E. coli*, which confers a wide spectrum of resistance to five chemically distinct classes of antimicrobials, was not tested with aminoglycosides [34]. We therefore need to confirm this gene as a possible basis for AR in the Kan10 clone with more detailed genotypic/phenotypic associations. As for the Amp3 clone, it also contains an acetyl transferase sequence belonging to the GCN5-related N-acetyltransferase (GNAT) superfamily of previously characterized gentamicin and kanamycin resistant bacteria [35]. Noteworthy, the remaining two sequenced clones (Amp1 and Amp6) do not appear to contain ORFs encoding protein functions commonly described in AR bacteria. They do, however, contain at least one ORF with the capacity to mediate bacterial antimicrobial resistance (Table 3).

In particular, clone Amp1 encodes a serine protease whose function includes serine beta-lactamase activities, which deactivate beta-lactam antibiotics by hydrolyzing the beta-lactam ring [36, 37]. The Amp6 clone, on the other hand, contains a phosphoglucomutase (PGM) ORF encoding the key enzyme catalyzing interconversion between glucose-1-phosphate (G1P) and glucose-6-phosphate (G6P) [38]. PGM plays a role in the biosynthesis of several bacterial exoproducts. Increased susceptibility to several antimicrobial agents was observed in* pgm* deletion mutants, suggesting a possible role in AR [39, 40]. Noteworthy, almost all clones also contain ORFs annotated as encoding “hypothetical proteins,” whose function might be related to AR. Although each clone contains a variable number of genes that could be related to AR, all of them display identical MIC values, suggesting that no additive effects due to the activity of multiple AR genes should be in place in any of the clones. Functional characterization of the putative AR gene sequences within the cloned fragments requires therefore further investigation. Another important feature deserving deeper analysis is the genomic context, as the sequencing output identifies a variable number of transposase genes within all sequenced inserts, usually clustered at a single site that likely represents an insertional hotspot. Transposases are integral parts of IS elements which mediate insertion/excision events known to promote lateral gene transfer events [41] and are especially important in horizontal gene transfer of AR genes.

## 4. Conclusions

We have reported in this work a novel metagenomic approach to identify AR genes within a complex, foodborne microbiome derived from a traditional fermented dairy product and constituted mainly by environmental strains of commensal bacteria. To increase the probability of identifying genes carried by underrepresented species, as well as to enhance representativeness of the library, we adopted a strategy based on direct cloning of total, unamplified DNA extracted from the food matrix, into a fosmid vector that can bear up to 40 Kbp inserts. Functional screening of the resulting metagenomic library, which we have calculated as representative of the entire microbiome, was carried out on antibiotics belonging to different pharmacological classes allowing recovery of ampicillin and kanamycin resistant clones. AmpR and KanR resistance genes are poorly characterized in LAB, although an important role for these bacterial genera as reservoir of transmissible AR genes is increasingly recognized [42]. Molecular characterization of the cloned inserts identified them as distinct regions of the* S. thermophilus* and* L. helveticus* genomes, hosting several ORFs which could confer AR phenotypes. The presence of several transposase sequences also emerged from full sequencing of the clone inserts, suggesting potential for lateral gene transfer of the surrounding genomic regions. This aspect is of special relevance, as IS mediated lateral gene transfer events represent the mechanistic basis for AR spreading from the reservoir of nonpathogenic, commensal bacteria to opportunistic pathogens [43]. From the food safety viewpoint, gene transfer events are particularly important as they might also occur through consumption of fermented foods and subsequent gene exchanges, which are known to occur between the food and the gut microbiota of the host [8, 44]. However, the low frequency of recovery of AR clones from our metagenomic library likely reflects a correspondingly low occurrence of AR bacteria in the food product, thus indicating its safe use for human consumption. Our results further support the evidence that metagenomic approaches can overcome the limitations of culture-dependent methods, representing an efficient and sensitive tool to detect genes occurring at low frequencies. Noteworthy, sequence analysis of the cloned inserts, which we had shown to retain the specific AR phenotype following transfer to new* E. coli* host cells, highlighted a number of genes whose involvement in AR might be novel. This observation points at the power of a screening strategy employing phenotypic selection, as, unlike primer-based methods that require known sequences as starting point, it can uncover novel genes performing similar functions. This work can therefore be considered a pioneer example of the application of metagenomics to food microbiota, and we hope it will pave the way to extend the strategy to other fermented foods, towards a deeper understanding of bacterial metabolic functions which could be beneficial to human health or of technological interest.

## Supplementary Material

Full-length sequences of the 3 AmpR and 1 KanR clone inserts. Sequence deposition in public databases is in progress.

## Figures and Tables

**Table 1:** Primers used for PCR experiments.

Primer pair	Sequence	Target gene	Reference
P0 P6	GAGAGTTTGATCCTGGCT CTACGGCTACCTTGTTAC	*Bacterial 16S rDNA *	[46]
SINE-F SINE-R	GGATCCGGCATTGCCGTTAG GTCTTTTTTTGCCATTTCTTGG	*Swine short interspersed nuclear elements *	[47]
ITS1 ITS4	TCCGTAGGTGAACCTGCG TCCTCCGCTTATTGATATGC	*Yeast 5.8S rDNA *	[48]
BufGH-F BufGH-R	TTGGGCCCCTGCAGTTC GGTCCGAGGTGCCAAACAC	*Buffalo growth hormone *	[49]

**Table 2:** Summary of screening procedure and resulting AR recombinant library clones.

Pharmacological class	Antibiotic	Target	*E. coli* MIC (mg/L)	Library clones identified	Verified by secondary screening
Tetracyclines	Tetracycline	Ribosome	5	4	0
Aminoglycosides	Kanamycin , gentamycin	Ribosome	25 12	2 0	2 —
Macrolides	Erythromycin	Ribosome	Resistant	—	—
Beta-lactams	Ampicillin	Cell wall	25	6	6
Glycopeptides	Vancomycin	Cell wall	Resistant	—	—

**Table 3:** Summary of insert sequencing results of AR fosmids. ORFs with a possible function in AR, as well as transposase genes, are listed.

	Amp1	Amp3	Amp6	Kan10
Insert length (bp)	14.380	38.386	21.644	33.491
Predicted ORFs (*n*)	14	43	21	35
Species	*S. thermophilus *	*S. thermophilus *	*S. thermophilus *	*S. thermophilus *
Relevant ORFs for AR	Serine endopeptidase	Penicillin-binding protein 2A Penicillin-binding protein 1A RNA methyltransferase GNAT family acetyltransferase	Phosphoglucomutase	23S rRNA methyltransferase
Transposase sequences (*n*)	2	5	10	1
MIC of the corresponding antibiotic (mg/L)	50	50	50	25
