# Supplementary material for: Functional Screening of Antibiotic Resistance Genes from a Representative Metagenomic Library of Food Fermenting Microbiota
Source: Biomed Res Int. 2014 Aug 28;2014:290967. doi: 10.1155/2014/290967 (PMC4163480; doi:10.1155/2014/290967)
Supplement: Supplementary file 1 — Full-length sequences of the 3 AmpR and 1 KanR clone inserts. Sequence deposition in public databases is in progress. [file 290967.f1.pdf]

Amp1 Contig 1

```
LOCUS       1A_1
DEFINITION
FEATURES             Location/Qualifiers
     gene            complement(181..2049)
                     /rbs_motif="AGGA"
                     /uscore="-0.74"
                     /rscore="7.92"
                     /sscore="10.91"
                     /locus_tag="1A_01"
                     /conf="99.99"
                     /ID="1A_01"
                     /cscore="380.2"
                     /partial=0
                     /tscore="3.07"
                     /gc_cont="0.418"
                     /rbs_spacer="5-10bp"
                     /start_type="ATG"
                     /score=391.1
                     /codon_start=1
     CDS             complement(181..2049)
                     /rbs_motif="AGGA"
                     /uscore="-0.74"
                     /rscore="7.92"
                     /sscore="10.91"
                     /locus_tag="1A_01"
                     /conf="99.99"
                     /ID="1A_01"
                     /cscore="380.2"
                     /partial=0
                     /tscore="3.07"
                     /gc_cont="0.418"
                     /rbs_spacer="5-10bp"
                     /product="phosphoribosylformylglycinamidine synthase"
                     /start_type="ATG"
                     /score=391.1
                     /codon_start=1
     gene            complement(2105..2812)
                     /rbs_motif="GGA/GAG/AGG"
                     /uscore="4.23"
                     /rscore="-1.46"
                     /sscore="5.18"
                     /locus_tag="1A_02"
                     /conf="100.0"
                     /ID="1A_02"
                     /cscore="123.87"
                     /partial=0
                     /tscore="3.07"
                     /gc_cont="0.381"
                     /rbs_spacer="11-12bp"
                     /start_type="ATG"
                     /score=129.1
                     /codon_start=1
     CDS             complement(2105..2812)
                     /rbs_motif="GGA/GAG/AGG"
                     /uscore="4.23"
                     /rscore="-1.46"
                     /sscore="5.18"
                     /locus_tag="1A_02"
                     /conf="100.0"
                     /ID="1A_02"
```

```
                     /cscore="123.87"
                     /partial=0
                     /tscore="3.07"
                     /gc_cont="0.381"
                     /rbs_spacer="11-12bp"
                     /product="phosphoribosylaminoimidazole-succinocarboxamide
                     synthase"
                     /start_type="ATG"
                     /score=129.1
                     /codon_start=1
     gene            complement(3097..3342)
                     /rbs_motif="GGAGG"
                     /uscore="0.41"
                     /rscore="14.07"
                     /sscore="18.75"
                     /locus_tag="1A_03"
                     /conf="100.0"
                     /ID="1A_03"
                     /cscore="24.57"
                     /partial=0
                     /tscore="2.98"
                     /gc_cont="0.333"
                     /rbs_spacer="5-10bp"
                     /start_type="ATG"
                     /score=43.3
                     /codon_start=1
     CDS             complement(3097..3342)
                     /rbs_motif="GGAGG"
                     /uscore="0.41"
                     /rscore="14.07"
                     /sscore="18.75"
                     /locus_tag="1A_03"
                     /conf="100.0"
                     /ID="1A_03"
                     /cscore="24.57"
                     /partial=0
                     /tscore="2.98"
                     /gc_cont="0.333"
                     /rbs_spacer="5-10bp"
                     /product="acyl carrier protein"
                     /start_type="ATG"
                     /score=43.3
                     /codon_start=1
     gene            complement(3342..4346)
                     /rbs_motif="None"
                     /uscore="4.68"
                     /rscore="-3.27"
                     /sscore="4.48"
                     /locus_tag="1A_04"
                     /conf="100.0"
                     /ID="1A_04"
                     /cscore="145.95"
                     /partial=0
                     /tscore="3.07"
                     /gc_cont="0.428"
                     /rbs_spacer="None"
                     /start_type="ATG"
                     /score=150.4
                     /codon_start=1
     CDS             complement(3342..4346)
                     /rbs_motif="None"
```

```
                     /uscore="4.68"
                     /rscore="-3.27"
                     /sscore="4.48"
                     /locus_tag="1A_04"
                     /conf="100.0"
                     /ID="1A_04"
                     /cscore="145.95"
                     /partial=0
                     /tscore="3.07"
                     /gc_cont="0.428"
                     /rbs_spacer="None"
                     /product="phosphate acyltransferase"
                     /start_type="ATG"
                     /score=150.4
                     /codon_start=1
     gene            complement(4562..5335)
                     /rbs_motif="GGA/GAG/AGG"
                     /uscore="-2.22"
                     /rscore="0.91"
                     /sscore="2.94"
                     /locus_tag="1A_05"
                     /conf="100.0"
                     /ID="1A_05"
                     /cscore="90.82"
                     /partial=0
                     /tscore="3.07"
                     /gc_cont="0.354"
                     /rbs_spacer="5-10bp"
                     /start_type="ATG"
                     /score=93.8
                     /codon_start=1
     CDS             complement(4562..5335)
                     /rbs_motif="GGA/GAG/AGG"
                     /uscore="-2.22"
                     /rscore="0.91"
                     /sscore="2.94"
                     /locus_tag="1A_05"
                     /conf="100.0"
                     /ID="1A_05"
                     /cscore="90.82"
                     /partial=0
                     /tscore="3.07"
                     /gc_cont="0.354"
                     /rbs_spacer="5-10bp"
                     /product="DNA repair protein RecO"
                     /start_type="ATG"
                     /score=93.8
                     /codon_start=1
     gene            complement(5322..6446)
                     /rbs_motif="None"
                     /uscore="0.03"
                     /rscore="-3.27"
                     /sscore="-0.17"
                     /locus_tag="1A_06"
                     /conf="100.0"
                     /ID="1A_06"
                     /cscore="98.61"
                     /partial=0
                     /tscore="3.07"
                     /gc_cont="0.373"
                     /rbs_spacer="None"
```

```
                     /start_type="ATG"
                     /score=98.4
                     /codon_start=1
     CDS             complement(5322..6446)
                     /rbs_motif="None"
                     /uscore="0.03"
                     /rscore="-3.27"
                     /sscore="-0.17"
                     /locus_tag="1A_06"
                     /conf="100.0"
                     /ID="1A_06"
                     /cscore="98.61"
                     /partial=0
                     /tscore="3.07"
                     /gc_cont="0.373"
                     /rbs_spacer="None"
                     /product="aminotransferase A"
                     /start_type="ATG"
                     /score=98.4
                     /codon_start=1
     gene            complement(7411..7578)
                     /rbs_motif="None"
                     /uscore="-2.35"
                     /rscore="-4.95"
                     /sscore="-3.98"
                     /locus_tag="1A_07"
                     /conf="78.86"
                     /ID="1A_07"
                     /cscore="9.71"
                     /partial=0
                     /tscore="-5.27"
                     /gc_cont="0.357"
                     /rbs_spacer="None"
                     /start_type="GTG"
                     /score=5.7
                     /codon_start=1
     CDS             complement(7411..7578)
                     /rbs_motif="None"
                     /uscore="-2.35"
                     /rscore="-4.95"
                     /sscore="-3.98"
                     /locus_tag="1A_07"
                     /conf="78.86"
                     /ID="1A_07"
                     /cscore="9.71"
                     /partial=0
                     /tscore="-5.27"
                     /gc_cont="0.357"
                     /rbs_spacer="None"
                     /product="CpsL protein"
                     /start_type="GTG"
                     /score=5.7
                     /codon_start=1
     gene            complement(7578..8402)
                     /rbs_motif="None"
                     /uscore="-1.49"
                     /rscore="-4.86"
                     /sscore="-11.77"
                     /locus_tag="1A_08"
                     /conf="50.0"
                     /ID="1A_08"
```

```
                     /cscore="11.42"
                     /partial=0
                     /tscore="-5.42"
                     /gc_cont="0.333"
                     /rbs_spacer="None"
                     /start_type="TTG"
                     /score=-0.4
                     /codon_start=1
     CDS             complement(7578..8402)
                     /rbs_motif="None"
                     /uscore="-1.49"
                     /rscore="-4.86"
                     /sscore="-11.77"
                     /locus_tag="1A_08"
                     /conf="50.0"
                     /ID="1A_08"
                     /cscore="11.42"
                     /partial=0
                     /tscore="-5.42"
                     /gc_cont="0.333"
                     /rbs_spacer="None"
                     /product="DNA polymerase III subunit beta"
                     /start_type="TTG"
                     /score=-0.4
                     /codon_start=1
     gene            complement(8557..9750)
                     /rbs_motif="GGA/GAG/AGG"
                     /uscore="1.54"
                     /rscore="0.91"
                     /sscore="4.86"
                     /locus_tag="1A_09"
                     /conf="99.99"
                     /ID="1A_09"
                     /cscore="175.61"
                     /partial=0
                     /tscore="3.07"
                     /gc_cont="0.371"
                     /rbs_spacer="5-10bp"
                     /start_type="ATG"
                     /score=180.5
                     /codon_start=1
     CDS             complement(8557..9750)
                     /rbs_motif="GGA/GAG/AGG"
                     /uscore="1.54"
                     /rscore="0.91"
                     /sscore="4.86"
                     /locus_tag="1A_09"
                     /conf="99.99"
                     /ID="1A_09"
                     /cscore="175.61"
                     /partial=0
                     /tscore="3.07"
                     /gc_cont="0.371"
                     /rbs_spacer="5-10bp"
                     /product="chromosomal replication initiation protein DnaA"
                     /start_type="ATG"
                     /score=180.5
                     /codon_start=1
     gene            complement(10266..10934)
                     /rbs_motif="AGGA"
                     /uscore="0.6"
```

```
                     /rscore="7.92"
                     /sscore="11.58"
                     /locus_tag="1A_10"
                     /conf="100.0"
                     /ID="1A_10"
                     /cscore="64.37"
                     /partial=0
                     /tscore="3.07"
                     /gc_cont="0.359"
                     /rbs_spacer="5-10bp"
                     /start_type="ATG"
                     /score=75.9
                     /codon_start=1
     CDS             complement(10266..10934)
                     /rbs_motif="AGGA"
                     /uscore="0.6"
                     /rscore="7.92"
                     /sscore="11.58"
                     /locus_tag="1A_10"
                     /conf="100.0"
                     /ID="1A_10"
                     /cscore="64.37"
                     /partial=0
                     /tscore="3.07"
                     /gc_cont="0.359"
                     /rbs_spacer="5-10bp"
                     /product="plasmid partitioning protein ParB"
                     /start_type="ATG"
                     /score=75.9
                     /codon_start=1
     gene            complement(11001..11588)
                     /rbs_motif="None"
                     /uscore="0.0"
                     /rscore="0.0"
                     /sscore="3.22"
                     /locus_tag="1A_11"
                     /conf="100.0"
                     /ID="1A_11"
                     /cscore="77.81"
                     /partial=1
                     /tscore="3.22"
                     /gc_cont="0.389"
                     /rbs_spacer="None"
                     /start_type="Edge"
                     /score=81.0
                     /codon_start=1
     CDS             complement(11001..11588)
                     /rbs_motif="None"
                     /uscore="0.0"
                     /rscore="0.0"
                     /sscore="3.22"
                     /locus_tag="1A_11"
                     /conf="100.0"
                     /ID="1A_11"
                     /cscore="77.81"
                     /partial=1
                     /tscore="3.22"
                     /gc_cont="0.389"
                     /rbs_spacer="None"
                     /product="Serine endopeptidase, partial"
                     /start_type="Edge"
```

```
                     /score=81.0
                     /codon_start=1
BASE COUNT     3562 a   2430 c   1949 g   3648 t
ORIGIN
        1 aatatccccg tggataagtg gataacccca agggaagttt tttcaggcat cgtgtgtaag
       61 cagaatatat aagtgctgtt ccctggtgct tcctcgctca ctcgaggctt cgccctgtcg
      121 ctcgactgcg gcgagtcacg acgttgtaaa acgacggcca gtgaattgta atacgactca
      181 ctatagggcg aattcgagct cggtacccgg ggatcccaca cgcacaccat ttgtatcaag
      241 gaaacgacgt tccaaatcaa caattgtttt accattccag tgcatgacga gatttggttt
      301 ttcagtcacg gttgcaacga caactgcatc aatattttct ttgttacatt cagcaacgaa
      361 gatatctaca tcttctggac gaaccaccac agccatacgt tcttgagatt cagagatggc
      421 aatttcggta ccgttcaaac cttggtattt caatggcact ttgtctagat caatttcaag
      481 accatctgcc aattcaccga tagccacaca gacaccacca gcaccaaagt cattggattt
      541 cttgataaga cgtgttactt caccattgcg gaaaagacgt tgaatcttac gttcttcgat
      601 agcattacct ttttgaacct cagcaccagc tgtttcaact gattcaaccg tttgaacttt
      661 tgaagaacct gtcgcaccac cgataccgtc gcgtccagtc ttaccaccga gcaagataac
      721 cacatctcca gcttcaggtt tttcacggac aacattctcc ttaggagctg caccgacaac
      781 tgcacctagc tccatacgct tggcaacgaa acctgggtgg aagtattcac gaacataagt
      841 tgttgcaaga ccaatttggt taccatatga agaataacca tgagccgctt ttttagaaat
      901 gacttgttgt ggcaatttc cagcacgtgt ttcagcgatt ggtgttgtaa tatcaccggc
      961 acctgaaata cgcatagctt ggtaaacgta tgaacgacct gacaatgggt cacgaatggc
     1021 accaccgata caagtagccg ctccaccaaa tggttcgatt tccgttgggt ggttatgagt
     1081 ttcgttcttg aacataagaa gccatggttc tttgacacca ttgacatcta cttcaatttc
     1141 aactgagcag gcattgattt cgtcagaaac ttccatgtca tccaaacggc cattggcacg
     1201 ctcataacga ccaaaaatag tcgccatatc cattaaggtt tgaggctttt ctgcacgtcc
     1261 caactcatca cgcatggcaa tatatttgtc ataagttgct tgcaattgtt tttggaattt
     1321 agaagctgag aagtcgatgt ttttcaactc agtctcgaaa gttgtgtggc ggcagtggtc
     1381 agaccagtaa gtatccaaaa ccttaagttc agtctcagtt ggcacacgtc caattgactt
     1441 gaagtaatct tgaatgaaga gcaggtcatc cacttccatg gccaatcctt gatctgcctt
     1501 atactgtgca aaatcttctg ctgtataagt ttcaaagaaa tccaagtttg gaatcgtttt
     1561 gtcagactca gagaaatcct gtttagcaat accaacagtg atatctttga aacgagagtc
     1621 cactgggttc aatagataat ttttaacagc ttccaattca ttagcagcaa tgtccttgtt
     1681 aaccaagtac aattgagcag tattaaccgt tacatcactt gagctaccaa gcaaaagcaa
     1741 agcttcttgt gaagatgctg cacgttgatc aaattgacca ggcaagcttt cgatagcaaa
     1801 gaaagcatac ttctcaaggt cagtcttaac tgtagcttca tccaaaacag tatcggtcac
     1861 ttgttcagaa aagatatatt tttccgcacg ggcaaacaaa tcctctgcca aattaaagac
     1921 atcgtaaact tgtacaatac gaagatcatt caaggttttc aactgaatat tatgctgtaa
     1981 ttcttttaat aaagagcgag atttcacacg aaaatcagct tttttctcaa cgaaaatacg
     2041 tttatccatt ttatttcctt tatctatttc cactaaaaga ttgtagtgac gaccaagtaa
     2101 aactttactt caatccttgc aatttttcca atacaacttc atacacatct gtcaagctac
     2161 cgagatcacg acggaaaaca tccttatcca tatggtgccc ttccgcatcc caaagacgac
     2221 agttatctgg tgaaaattca tctgccaaga taatcttgcc atccttatca aaaccaaact
     2281 ccaatttgaa gtcaatcaaa cgaagaccaa tttgctcaaa ccaatctttt agcaattcgt
     2341 tgatacgacg tgtttcctcc ttgatataag caatttgctc atcattagca atatccaaaa
     2401 acttcacatg ctcatcattg ataaatggat catccaaatc atcgtttttg tagtaaaatt
     2461 caacaattgg agttttcaag tccagacctt cttccacacc gaaacgtttt gagaaagaac
     2521 ccgcagtcac gttacgaagc acaacctcca aaggaatgat tgtaaccttc ttgttaagtt
     2581 gttctgtatc agagatacgt tcgataaaat gagtagccac acccgcagcg ttcaattttt
     2641 caaaaataag agacgaaatc tgattattaa gcacgccttt ccctttaatg ttctccttac
     2701 gagcaccatt aagcatggtt gcctggtcct tatagaccga cttaatcaca ttttcgtctt
     2761 ctgtactata aatatcttta gctttcccag tataaatcag ttggtttgac atagaatcgt
     2821 tcgcctttca agtttgttca tcttaattat atcacacaaa aatccaaaaa caaacatata
     2881 tcaaactttt ttgttttatt aaacaaataa cgttcggatt tttagtattg gttagagttt
     2941 tttaataaaa aagtatagaa aaaaggagtg agatagtgct aaatcaatgt aacttttagc
     3001 tttttatctc actccaataa aatgaaaaca tgcaacaaca tatagttgct tcattattat
     3061 attcctctag aatctaacaa ctagagtcta gttttcttat ttgttattca attgctcaga
     3121 caaatagtct aacaaatctg ataccttatc catatgatca atggtattat cagaaatctc
     3181 tagggaaaac tcatcctcca aattaataat gaactccata aactctattg aatctacacc
     3241 caagtcattt aatttggtag cttcggttaa aacaatgtcc tctctatcca attgctcttg
     3301 aataacgagc tgcatttcct gaagaatttc tactctactc atcagttaac ctcctctttc
     3361 gggtcgaaag cgtcaactag ttgctccacc accttagtat cgagcatctt acgaacctgc
```

3421 ttaatggtat agtagactgc tttagcatca ctagatccat gacattttac tacaggagcc
3481 ttgagcccaa aaagaacagc tccaccggca cttgagtaat ccatcgtctc tttcatacct
3541 ttaagtctat ccttcaatag gaaggcacct aacttagctt tgagaccacc attcattatg
3601 gctgacttaa gagtcttcat tgtgccaagt ccagttcctt caattgcctt aagaaccgca
3661 tttcctgtga aaccatcagc cactaccaca tcagcagctc cagacatgat ttcacgcgcc
3721 tcgacattac caatgaagtt aatacttgct tccttggata aaagctcaaa agcctcctta
3781 cgaagactat cccccttgt tttctcagtc ccattattaa ggaggcctac acgaggggtg
3841 gcaattccac gtacattttt agcatagaaa gagcctaaaa tagcatattg atgcaagtga
3901 ttagctgtat tttcagcatt agctcctaga tcaagcatat caaagggttg accagtaaaa
3961 cttggcatcg ttgacataag acctggtctt tcaactccct taatacgtcc aacaacaaaa
4021 agaccggcgg ccaaaagagc acctgtattc cctgcagaga ttacagcatc cgctgctttc
4081 tccttaacag cttgtgcacc caaaaccatc gaagctttct ttttctttcg aattgctttg
4141 acgggctcgt catccgaatt tatcttttca tcagtatgta caatgctcac acgttcattc
4201 gctatcaaat agtttttgat tttagcttcg tcaccataaa gctggatttc aatatctttg
4261 aactctgcta aagcttggtt aactccctct acgatcgctt gtggcgcatt atcgccaccc
4321 atagcatcca ctgcaattac aggcatagca ttctccctct catcctctgc tcttaaacaa
4381 tagactcaaa ttctattaat aaatcactct gtatcaaaga tagacatccc tcggtcacta
4441 tccccagcag agtctttcag cttattgtcc tcttattata gcacgaactg acctgcattt
4501 taacccctga gactgaaaac ctctacattc ctgtttcacg tgaaacaaaa gaagacaatg
4561 cttaatcagc attatcttct ttactcataa tatgacccca agaatttaga ttatcaataa
4621 acttcttgct tttaagatga atcccaacat aattatcata aagctcatca ataaattgtc
4681 tcaactttcg tttcatttca tccttaacag atatgcttct caattcttca aacgaaagac
4741 cctggaaacg atccaaaaga taaggcacat tagggtccaa atgactacgc ctttcatctt
4801 cttcgtaatg attgggacaa agtaaacctg agaacttata cgagaaatca aaaggaagac
4861 caacacgatg acaaaagaca cattcatgaa aattcaatcg tacgccaaaa cggtccaaaa
4921 cttgaatctc aaagatatta gtcaatattt cataatctaa gccctcttcc attaacacaa
4981 gggtttctc taaaaatgca aataattgtg catctaccac accgtcagca atagcagcat
5041 cagtcaaggc agctaaatag gtcgcgtaag acagtttaaa aatatcactt gtaatttcct
5101 taaaagactc tgcctgacta taatcctcta tgaaagataa accttcatca tttacagtca
5161 tcataaattc agcgatgttt aaaggttgaa tcacagcact caatttacct gacccaccac
5221 ctctaacaaa aaacatccgt ttaccagctt gtttagtaaa aatcttaacc agcttatcat
5281 tctcacgata attacgattg aagaggataa accccctact ctcaagcttc tgcatgttct
5341 tccataaact ctttcaaacg agtcatagct gtttcaatag tgtccatgct agctgcatac
5401 gacaatcgaa cataaccttc gccatatttc ccaaaagcaa cgccaggaat aatagcaata
5461 gctttttcac gagcaaagtc ttgacagaac ttaaaggagt cttgattata tccttctgga
5521 atcttagcaa agatatagaa ggcaccatca ggtttaataa ttttgaaacc aagttcagtc
5581 atttttttcaa gaatataatc ccgacgtttc aaatactcaa ctttcattgg ttgtgcatca
5641 tccttaccag cagataatgc ttcgatagct gcaaattgtg ccatagtagc tgcagccgtc
5701 accaaatact ggtgactctt aatcaactga gccgtcaaac tttctggtgc aaaaataaaa
5761 ccaatacgcc atcctgtcat cgcatgagat ttcgacaagc cgttgattaa aatagtttgc
5821 tcaggcaaat attcagcaat agaaacatgt tgatctacat aagttagttc tgaatatact
5881 tcatcactaa caacaaaaat atcatatttt ttcagaactt cagcaatatc ctcaatctgt
5941 tggcgtgaat aagtcacacc tgttgggttg gttgggtagt taagtaaaac agccttaagc
6001 ttatcacctt gctctaaaat tgccttttct aacatctccg gtgtcaaaac aaaatcatta
6061 gctgttgtat caatttcaac aattttagcc ccaactagat ttgcaatcgg ctcatatcct
6121 ggataggctg gtgcaggcaa aagaatcgta tcacctggct ccaaaatagc tgttaaagac
6181 gcagaaagag cttctgtggc cccaatagtt accaaaattt cattatcggg attataagtt
6241 agattgtatt tttccttaac aaaattcgca gcagcttggc gcaaatcagg aagtccagcc
6301 atacctgtat agtgtgactg gttcgcatca atcgcagttt tagctgcttc tttcacatga
6361 tctggagtcg taaaatcagg ttctccaagc gtcaactttt taatacctgg aacatctgaa
6421 attgattggt caaactgacg aatcattgag acttcaatct tatctaaatt ttttattaaa
6481 acgttgtgtt aaatccatca cagtcacctc tttgcaatta ttcctaacat tatatcaaaa
6541 actcaaacgc atttaaacac caaaaaactg tcctaggcta gctttaatgg ctctataatt
6601 tctgtagggg gtaaaaacca ctgtagagat tatggagctt tttaaatgca gacaaaaagt
6661 tccataagaa ctataatgaa aagcgactaa accatcatta gcaagaactc atgagatcta
6721 ttaagaatac cacagagtta aatcccttaa catcattact tctcttgttt tcgaaaaaga
6781 tacctacatc gaggttcaag caaaactgga actccaacac tacgacgcct gaagaaacat
6841 cgcttacagt gtaaagattg tagaaaagtc acggttgctg agacattaat cgttgagaaa
6901 aaccaccaaa tctccaatct tatccgacaa aaggtaaggt ttatcacgat ggacatgtca
6961 ggagcttata ttccactagt tcgaagactt tcctaaatgc ccaaatcatt attgatcgtt
7021 tccacatcat ccagcagctt gatcaagcct ttttaaagac aagaattgcc attatgaacc

7081 agttcaataa gaagccacta ccttatcgat ctttaaaaat cactggagac tcgccgtaag
7141 ttatcttgta aatcatttca ctcgaagacc ttttgtcaaa ccttaaaccc acatgaagtc
7201 gttgagaaga cactgaattt ctcagaagag atcgctaatt attacaacct ctatcaacct
7261 ttgctttttc actttcagga taagagggtg aatgagtttt ttcaccccat agaggagaaa
7321 ataagcaagg tcaattatta cttcaaaaca gtctttagga cctttcttag acacaaacga
7381 tacatcaaaa atgcaccaaa aacacagtcc tcacatgcaa aattggaggg gaccaataag
7441 cttatcaaag atattaaacg tctgggcttc ggttttagaa atttattaa ctttaggaag
7501 catgttttca tcactcttaa catacaaaaa gagaagacct atctgatcct ctctagatgt
7561 tagcttttcg tcacccacta cagttgacaa aaagccgatg tttcaaacac aacctctgtt
7621 tcaaatgaat ctgacaagag acgattagta tctggataat ttccttctag taaacgtgtg
7681 taaaaagaga ttgtctcact acggaataga atttgatttg cagagaagaa aatttccaaa
7741 ttttcaatac catcactaaa gactgctgat aactctttaa gtgatcggct tgggattact
7801 acatcaaagt tatctgcaga atgttccaaa acaagttgac gttggctcat acgatgagaa
7861 tcagtggcaa cagctttaag atctttgtga tttgaaagaa caaagtgaac tccagttaaa
7921 attggacgac tttcttgcgt gcttgctgca aatgacgttt cagcaatgat agattttaac
7981 attttagtgt ccataattag tggtgtcaag ctgtcaactt cttgaatacg aggatattgc
8041 tcagcatcct ttcctttag agtaatctct gatttagcac tctttagtaa tacttggtat
8101 tgttctattt cttcaaatgt aagagtaaca tcaggtaagc ttgaaacaac gttgatgaaa
8161 aaattcgctt ctaaaaggat actacctggt tgtgtaatta ataaacctgc atttcttct
8221 gaagcagaga tgctattctc tatcgagatt tgtccatttg agccagttaa atagatacca
8281 tcactagtaa cttcgatttt tatagttgat agaatgggaa tagcattttt tgaagaaata
8341 gcacgtcgag ttgtatgaag ggcttgtagg aaataagctt tattaatggc aaattgaatc
8401 atgttgctcc tttattttat tagtattatg ttagtagtag tagtaggtct tgtggatatt
8461 gtgcataaat atctaaatca cagttttatc aagattttaa acttgtgcac atcttgtgga
8521 aaactgtgta taagttttag tagttatcca caagtgttat tttatcttgt ttttaatggt
8581 atcaatttca atgcgtaaac tgtcatcttg agcaagcata tttttgattt tattgtaagc
8641 atgtagaaca gttgagtgat ctcttcctcc aaattctttt ccaattttg gaagggagtt
8701 atcagtcatt tcacgcgcaa gatacatggc aacctgacgc gctaagacaa tattttgtgt
8761 tcgtttagtt gccttaatct ctttaaccgt gacattataa aatttcccaa cctcactttg
8821 gatgtcttca atcggaatga gagttaactt agggccatct attttacgtg cacgaatagc
8881 ttcggcagct acttcaactg tgatcgtatc taactggcgg acatttgcaa ctaagctaat
8941 atctttcaaa gctccctcta agtcgcggac atttgagtca aattggccag ctaaatactc
9001 aattgtttca ggtggaaaat ggtaatcata gtccataatc ttattggtaa gaatagcaac
9061 tcgggtttca aagtcaggcg gtgtaatatt tacagtcaag ccccatttga agcgcgtgac
9121 aagacgctct tccatttcat tcaattgatc tggatttcgg tcgctagtca agacaatttg
9181 tttattatta tcgtgaagga cattgaaagt attgaaaaat tcctcttgag tggcttgcgt
9241 agacttttta gccaacgatt gtatatcatc aattaaaagc acgtctagat ggcggtattt
9301 tagctttagt tcatccattt tatctaaacg gatatgtaaa acaaactcat taataaaatt
9361 ttcggcagtt atgtatttga ttcgtgcttg gggattatca tgtaagacct tattaccaat
9421 ggcattgagc aagtgagttt ttcctagacc aggtccccca taaatgaata aagggttata
9481 agttgctcct ggtgaatcag ctacagctag agatgctgct acagaccagc ggttttcatc
9541 tccttgaata aaattatcaa aggtatactt actatttaag tctgagttca aagtgggaag
9601 atcaggtgtt tgttgttccc ttttattgct gaagtcattg tttgctaaag ttggttttgt
9661 ttcgctaacg agagcctcgt caaagacata ttcaactttt atttcactat tgtagacttc
9721 aaatccagtc atcaaaatga aagattgcat gttcttttcc cagaatagtt tcttgacttc
9781 ttcaagcaga attgtagcaa cgttatcttg aattttgatg agcttggtat ctaggacata
9841 ataatcaaaa gtggcttgtg ttagttcttc tttggccagc tctaagagct tattccaaaa
9901 aaaaatgctc tttttcggtc attttatcaa ttctccatct ttattcttgt cctctgattc
9961 tatcataaaa cgaaaaagtt ttccacaggt cagaaagaga aaagttttga ggttatccac
10021 aaccataaaa aaaggttatt cacactgtga ataaaagttt tccacagcct gttgataata
10081 aagaaaccct ttaaaataaa gggtagacac tatttttacg atgtggataa gtctgattta
10141 ccttttttcta caattaacag cttagataag cttgttgata attctgttaa aatcttcttc
10201 tgtcgaaaag tgtatttgaa tcgttcctga gtgatttttg tgataacgta gagttatggg
10261 gagacctagt gatctttcaa gtttcttttt tctttatctt ttacatgggt atcttttttta
10321 gatttttttgt tttctaggaa gttcctgaag ttttttttttt ctagagtacg aacagaccat
10381 ctttcagcca caacctgctg aaagatggtt aactgctttc gagaatcctc aatagctaag
10441 agtgctctag catgtccctg actcagactt ccgttttcaa tactcgtttg tatctctcgc
10501 ggaagttgta gtagtctaag agcgttacta atatatggtc tagattttcc catagcttta
10561 gcgagctcct cgtgactata agttaactca tcaattaaat tacgataggc ctttgcttct
10621 tcaataggat tgagatttga tcgttgtaga ttttcaataa tagcttgttt acgactctct
10681 tgatctgtaa cttcctttac aactgcaggg atttctgtca tccctaaacg tttacaggct

10741 cgtaaacgtc tttctccagc aattagttca taaccaataa tgtcagattt tcgtacaata
10801 attggctgta ttaaaccatt ttctgatatt gattgactta gttctactaa ctcttcatcg
10861 gagaatttta gacgaggttg aaaaggattg gggtaaattt cactaataga taaagttttg
10921 agttgttctg acatggtatt cctttcccaa aaaaagacct ttacattatt gtaaaggtct
10981 tttgacagat ttgtcaataa ttaatcagat atgtcttctg tagattttgt taatttaaga
11041 tctacagttt tcttatcttt accacgatag aaggttacct tgatagtatc tccgatacta
11101 tggctgtaaa gattggtttg aatatcagtt tttgagctga ttttcttacc atcaatctca
11161 gtgataacat catattcttg aagtttaccg tcagctggca tacctttctg aacactaccg
11221 acaacaacac cggatttgac ctcatctggt aaatctaatt tagaagttgc gctgctagaa
11281 aggctattaa gatctgctat tgagatccct agtgctggtc gtgtaacttt accatctttt
11341 tctaattgat tgatgatttc aacaacatcg tttgatggaa tggcaaaccc cataccttca
11401 acagcactac cagcgactgc agacgttgat gaaattttac ttgagttaat accgataact
11461 tgtccttcaa tattgactag ggcaccacca gagttaccag tgggatcctc tagagtcgac
11521 ctgcaggcat gcaagcttga gtattctata gtctcaccta aatagcttgg cgtaatcatg
11581 gtcatagct
//

Amp1 contig 2

```
LOCUS       1A_2
DEFINITION
FEATURES             Location/Qualifiers
     gene            1..255
                     /rbs_motif="None"
                     /uscore="0.0"
                     /rscore="0.0"
                     /sscore="3.22"
                     /locus_tag="1A_12"
                     /conf="99.29"
                     /ID="1A_12"
                     /cscore="18.26"
                     /partial=10
                     /tscore="3.22"
                     /gc_cont="0.412"
                     /rbs_spacer="None"
                     /start_type="Edge"
                     /score=21.5
                     /codon_start=1
     CDS             1..255
                     /rbs_motif="None"
                     /uscore="0.0"
                     /rscore="0.0"
                     /sscore="3.22"
                     /locus_tag="1A_12"
                     /conf="99.29"
                     /ID="1A_12"
                     /cscore="18.26"
                     /partial=10
                     /tscore="3.22"
                     /gc_cont="0.412"
                     /rbs_spacer="None"
                     /product="hypothetical protein"
                     /start_type="Edge"
                     /score=21.5
                     /codon_start=1
     gene            complement(430..1758)
                     /rbs_motif="GGAG/GAGG"
                     /uscore="4.62"
                     /rscore="6.9"
                     /sscore="7.88"
                     /locus_tag="1A_13"
                     /conf="100.0"
                     /ID="1A_13"
                     /cscore="166.07"
                     /partial=0
                     /tscore="-3.65"
                     /gc_cont="0.414"
                     /rbs_spacer="5-10bp"
                     /start_type="TTG"
                     /score=174.0
                     /codon_start=1
     CDS             complement(430..1758)
                     /rbs_motif="GGAG/GAGG"
                     /uscore="4.62"
                     /rscore="6.9"
                     /sscore="7.88"
                     /locus_tag="1A_13"
                     /conf="100.0"
                     /ID="1A_13"
```

```
                     /cscore="166.07"
                     /partial=0
                     /tscore="-3.65"
                     /gc_cont="0.414"
                     /rbs_spacer="5-10bp"
                     /product="transposase"
                     /start_type="TTG"
                     /score=174.0
                     /codon_start=1
     gene            complement(1851..2789)
                     /rbs_motif="None"
                     /uscore="-2.87"
                     /rscore="-4.19"
                     /sscore="-10.51"
                     /locus_tag="1A_14"
                     /conf="82.35"
                     /ID="1A_14"
                     /cscore="17.21"
                     /partial=0
                     /tscore="-4.67"
                     /gc_cont="0.374"
                     /rbs_spacer="None"
                     /start_type="TTG"
                     /score=6.7
                     /codon_start=1
     CDS             complement(1851..2789)
                     /rbs_motif="None"
                     /uscore="-2.87"
                     /rscore="-4.19"
                     /sscore="-10.51"
                     /locus_tag="1A_14"
                     /conf="82.35"
                     /ID="1A_14"
                     /cscore="17.21"
                     /partial=0
                     /tscore="-4.67"
                     /gc_cont="0.374"
                     /rbs_spacer="None"
                     /product="transposase, partial"
                     /start_type="TTG"
                     /score=6.7
                     /codon_start=1
BASE COUNT      723 a    514 c    632 g    918 t
ORIGIN
        1 cattttttccg cactccttac acttgaaacg gcgctttttc aggcgaatga gagttcggta
       61 gccagcacac tccagatacg ggattttaga ggctttctgg aagtcgtatt tgcccatctg
      121 tcccttgcag gcaggacatt taggagactc gtaatccaag taaccgtgaa gttctttgtg
      181 cattcccata tcgtattcat tagtgatgat aatatttttg tcttttattc caagaaaatt
      241 tgtgataaga tttagttgtt ccatatgagt ctttctaaaa tgatggttta gtcgcttttc
      301 attataggtc atatgggact tttttctac aatcaaaaag gctccataat ctccatagag
      361 gatttaccca ctacagaaat tatagagcct gananggctc tttgtcaact gtagtgggta
      421 gatgaaaagc taaattcttg agaggaccag tttggtcttc tcttttttaa tatttactgc
      481 catcaaaatc cgtttcttaa agttttcaaa gttcctgaag ccaaaggcat ttcgcttaat
      541 gaccttgatg agattgttcg ttgcttccag ttttgcgttt gaatacggca gttcgagggc
      601 gttgataatc ttgtctttat ctttcaaaaa agttctaaaa actgtctgaa agatagggtg
      661 tacgctggaa ctcgtctctt caatgaggtc aaagaaatgg tccgagttct tctcttggaa
      721 atggaaaagt aaaagttggt agagttcata atgttctctc agttcttgag agtatgacag
      781 tagcttttct aggatttcct tgttggttaa atgcgcacga aaagtcggac gataaaagcg
      841 tttatcgttg agtttgcgac tatcttgttg gatcagtttc cagtagcgtt tcagggcacg
      901 atattcctgt gactgacggt ccaaatggtt catgatttga atgcggacgc ggttcatagc
      961 acgactaagg tgttgcacaa tgtgaaagcg atcaaggaca atcttagcgt tagggaataa
```

1021 ttttctggcg atgtcgtaat aagggctaaa catgtccata gtgatgactt taacgcgatt
1081 tctatcctgt ctggaatagc gtaggaagtg gtttctaatc gttgcttgcg tgcgtccgtc
1141 gaggattgtg atgactttta atgagtcgaa atcctgagcg atgaaactca tttttccctt
1201 cttgaagccg tactcatccc agctcatatt ctcaggcagc caattccagt cggttttaaa
1261 ttggaactca tttagcttgc gactgacggt tgaggtagag acggcaaggc gcttggcaat
1321 gtcggtcata gactgttttt caatgagcaa ttgagcgatt ttctgataga cgacggttgc
1381 gatttggtga ttcttcttga ccagagaagt ttccgcgaca gccatttttc cgcactcctt
1441 acacttgaaa cggcgctttt tcaggcgaat gagagttcgg tagccagcac actccagata
1501 cgggatttta gaggctttct ggaagtcgta tttgcccatc tgtcccttgc aggcaggaca
1561 tttaggagac tcgtaatcca agtaaccgtg aagttctttg tgcattccca tatcgtattc
1621 attagtgatg ataatatttt tgtcttttat tccaagaaaa tttgtgataa gatttagttg
1681 ttccatatga gtctttctaa aatgatggtt tagtcgcttt tcattatagg tcatatggga
1741 cttttttttct acaatcaaaa aggctccata atctccatag aggatttacc cactacagaa
1801 attatagagc cnnaaaggct ctttgtcaac tgtagtgggt agatgaaaag ctaaattctt
1861 gagaggacca gtttggtctt ctcttttta atatttactg ccatcaaaat ccgtttctta
1921 aagttttcaa agttcctgaa gccaaaggca tttcgcttaa tgaccttgat gagattgttc
1981 gttgcttcca gttttgcgtt tgaatacggc agttcgaggg cgttgataat cttgtcttta
2041 tctttcaaaa aagttctaaa aactgtctga aagatagggt gtacgctgga actcgtctct
2101 tcaatgaggt caaagaaatg gtccgagttc ttctcttgga aatggaaaag taaaagttgg
2161 tagagttcat aatgttctct cagttcttga gagtatgaca gtagcttttc taggatttcc
2221 ttgttggtta aatgcgcacg aaaagtcgga cgataaaagc gtttatcgtt gagtttgcga
2281 ctatcttgtt ggatcagttt ccagtagcgt ttcagggcac gatattcctg tgactgacgg
2341 tccaaatggt tcatgatttg aatgcggacg cggttcatag cacgactaag gtgttgcaca
2401 atgtgaaagc gatcaaggac aatcttagcg ttagggaata attttctggc gatgtcgtaa
2461 taagggctaa acatgtccat agtgatgact ttaacgcgat ttctatcctg tctggaatag
2521 cgtaggaagt ggtttctaat cgttgcttgc gtgcgtccgt cgaggattgt gatgactttt
2581 aatgagtcga aatcctgagc gatgaaactc atttttccct tcttgaagcc gtactcatcc
2641 cagctcatat tctcaggcag ccaattccag tcggttttaa attggaactc atttagcttg
2701 cgactgacgg ttgaggtaga gacggcaagg cgcttggcaa tgtcggtcat agactgtttt
2761 tcaatgagca attgagcgat tttctgatag a
//

Amp3

```
LOCUS       3A
DEFINITION
FEATURES             Location/Qualifiers
     gene            284..1903
                     /rbs_motif="AGGAG"
                     /uscore="6.97"
                     /rscore="11.88"
                     /sscore="21.91"
                     /locus_tag="3A_01"
                     /conf="99.99"
                     /ID="3A_01"
                     /cscore="406.56"
                     /partial=0
                     /tscore="3.07"
                     /gc_cont="0.423"
                     /rbs_spacer="5-10bp"
                     /start_type="ATG"
                     /score=428.5
                     /codon_start=1
     CDS             284..1903
                     /rbs_motif="AGGAG"
                     /uscore="6.97"
                     /rscore="11.88"
                     /sscore="21.91"
                     /locus_tag="3A_01"
                     /conf="99.99"
                     /ID="3A_01"
                     /cscore="406.56"
                     /partial=0
                     /tscore="3.07"
                     /gc_cont="0.423"
                     /rbs_spacer="5-10bp"
                     /product="molecular chaperone GroEL"
                     /start_type="ATG"
                     /score=428.5
                     /codon_start=1
     gene            1996..2262
                     /rbs_motif="None"
                     /uscore="1.38"
                     /rscore="-3.27"
                     /sscore="1.18"
                     /locus_tag="3A_02"
                     /conf="99.54"
                     /ID="3A_02"
                     /cscore="22.17"
                     /partial=0
                     /tscore="3.07"
                     /gc_cont="0.322"
                     /rbs_spacer="None"
                     /start_type="ATG"
                     /score=23.4
                     /codon_start=1
     CDS             1996..2262
                     /rbs_motif="None"
                     /uscore="1.38"
                     /rscore="-3.27"
                     /sscore="1.18"
                     /locus_tag="3A_02"
                     /conf="99.54"
                     /ID="3A_02"
```

```
                     /cscore="22.17"
                     /partial=0
                     /tscore="3.07"
                     /gc_cont="0.322"
                     /rbs_spacer="None"
                     /product="transposase"
                     /start_type="ATG"
                     /score=23.4
                     /codon_start=1
     gene            2344..2511
                     /rbs_motif="GGAG/GAGG"
                     /uscore="0.66"
                     /rscore="4.56"
                     /sscore="7.24"
                     /locus_tag="3A_03"
                     /conf="97.63"
                     /ID="3A_03"
                     /cscore="8.93"
                     /partial=0
                     /tscore="2.02"
                     /gc_cont="0.405"
                     /rbs_spacer="5-10bp"
                     /start_type="ATG"
                     /score=16.2
                     /codon_start=1
     CDS             2344..2511
                     /rbs_motif="GGAG/GAGG"
                     /uscore="0.66"
                     /rscore="4.56"
                     /sscore="7.24"
                     /locus_tag="3A_03"
                     /conf="97.63"
                     /ID="3A_03"
                     /cscore="8.93"
                     /partial=0
                     /tscore="2.02"
                     /gc_cont="0.405"
                     /rbs_spacer="5-10bp"
                     /product="transposase"
                     /start_type="ATG"
                     /score=16.2
                     /codon_start=1
     gene            2622..2840
                     /rbs_motif="None"
                     /uscore="1.25"
                     /rscore="-3.78"
                     /sscore="-6.56"
                     /locus_tag="3A_04"
                     /conf="95.09"
                     /ID="3A_04"
                     /cscore="19.45"
                     /partial=0
                     /tscore="-4.02"
                     /gc_cont="0.411"
                     /rbs_spacer="None"
                     /start_type="GTG"
                     /score=12.9
                     /codon_start=1
     CDS             2622..2840
                     /rbs_motif="None"
                     /uscore="1.25"
```

```
                     /rscore="-3.78"
                     /sscore="-6.56"
                     /locus_tag="3A_04"
                     /conf="95.09"
                     /ID="3A_04"
                     /cscore="19.45"
                     /partial=0
                     /tscore="-4.02"
                     /gc_cont="0.411"
                     /rbs_spacer="None"
                     /product="transposase, partial"
                     /start_type="GTG"
                     /score=12.9
                     /codon_start=1
     gene            2898..3125
                     /rbs_motif="GGA/GAG/AGG"
                     /uscore="3.62"
                     /rscore="-1.62"
                     /sscore="5.41"
                     /locus_tag="3A_05"
                     /conf="99.59"
                     /ID="3A_05"
                     /cscore="18.52"
                     /partial=0
                     /tscore="2.76"
                     /gc_cont="0.342"
                     /rbs_spacer="11-12bp"
                     /start_type="ATG"
                     /score=23.9
                     /codon_start=1
     CDS             2898..3125
                     /rbs_motif="GGA/GAG/AGG"
                     /uscore="3.62"
                     /rscore="-1.62"
                     /sscore="5.41"
                     /locus_tag="3A_05"
                     /conf="99.59"
                     /ID="3A_05"
                     /cscore="18.52"
                     /partial=0
                     /tscore="2.76"
                     /gc_cont="0.342"
                     /rbs_spacer="11-12bp"
                     /product="transposase"
                     /start_type="ATG"
                     /score=23.9
                     /codon_start=1
     gene            complement(3313..3621)
                     /rbs_motif="None"
                     /uscore="0.15"
                     /rscore="-3.27"
                     /sscore="-0.05"
                     /locus_tag="3A_06"
                     /conf="97.49"
                     /ID="3A_06"
                     /cscore="15.96"
                     /partial=0
                     /tscore="3.07"
                     /gc_cont="0.388"
                     /rbs_spacer="None"
                     /start_type="ATG"
```

```
                     /score=15.9
                     /codon_start=1
     CDS             complement(3313..3621)
                     /rbs_motif="None"
                     /uscore="0.15"
                     /rscore="-3.27"
                     /sscore="-0.05"
                     /locus_tag="3A_06"
                     /conf="97.49"
                     /ID="3A_06"
                     /cscore="15.96"
                     /partial=0
                     /tscore="3.07"
                     /gc_cont="0.388"
                     /rbs_spacer="None"
                     /product="hypothetical protein Y021_01175"
                     /start_type="ATG"
                     /score=15.9
                     /codon_start=1
     gene            complement(3587..3943)
                     /rbs_motif="GGAG/GAGG"
                     /uscore="-1.98"
                     /rscore="6.9"
                     /sscore="2.53"
                     /locus_tag="3A_07"
                     /conf="100.0"
                     /ID="3A_07"
                     /cscore="41.29"
                     /partial=0
                     /tscore="-3.65"
                     /gc_cont="0.367"
                     /rbs_spacer="5-10bp"
                     /start_type="TTG"
                     /score=43.8
                     /codon_start=1
     CDS             complement(3587..3943)
                     /rbs_motif="GGAG/GAGG"
                     /uscore="-1.98"
                     /rscore="6.9"
                     /sscore="2.53"
                     /locus_tag="3A_07"
                     /conf="100.0"
                     /ID="3A_07"
                     /cscore="41.29"
                     /partial=0
                     /tscore="-3.65"
                     /gc_cont="0.367"
                     /rbs_spacer="5-10bp"
                     /product="hypothetical protein"
                     /start_type="TTG"
                     /score=43.8
                     /codon_start=1
     gene            complement(3936..4037)
                     /rbs_motif="GGAG/GAGG"
                     /uscore="1.38"
                     /rscore="2.73"
                     /sscore="5.33"
                     /locus_tag="3A_08"
                     /conf="90.9"
                     /ID="3A_08"
                     /cscore="4.68"
```

```
                     /partial=0
                     /tscore="1.21"
                     /gc_cont="0.422"
                     /rbs_spacer="5-10bp"
                     /start_type="ATG"
                     /score=10.0
                     /codon_start=1
     CDS             complement(3936..4037)
                     /rbs_motif="GGAG/GAGG"
                     /uscore="1.38"
                     /rscore="2.73"
                     /sscore="5.33"
                     /locus_tag="3A_08"
                     /conf="90.9"
                     /ID="3A_08"
                     /cscore="4.68"
                     /partial=0
                     /tscore="1.21"
                     /gc_cont="0.422"
                     /rbs_spacer="5-10bp"
                     /product="hypothetical protein"
                     /start_type="ATG"
                     /score=10.0
                     /codon_start=1
     gene            complement(4132..4368)
                     /rbs_motif="None"
                     /uscore="-3.5"
                     /rscore="-3.49"
                     /sscore="-4.12"
                     /locus_tag="3A_09"
                     /conf="79.71"
                     /ID="3A_09"
                     /cscore="10.07"
                     /partial=0
                     /tscore="2.87"
                     /gc_cont="0.405"
                     /rbs_spacer="None"
                     /start_type="ATG"
                     /score=6.0
                     /codon_start=1
     CDS             complement(4132..4368)
                     /rbs_motif="None"
                     /uscore="-3.5"
                     /rscore="-3.49"
                     /sscore="-4.12"
                     /locus_tag="3A_09"
                     /conf="79.71"
                     /ID="3A_09"
                     /cscore="10.07"
                     /partial=0
                     /tscore="2.87"
                     /gc_cont="0.405"
                     /rbs_spacer="None"
                     /product="putative uncharacterized protein"
                     /start_type="ATG"
                     /score=6.0
                     /codon_start=1
     gene            complement(4438..4770)
                     /rbs_motif="AGGAG"
                     /uscore="5.99"
                     /rscore="11.88"
```

```
                     /sscore="20.28"
                     /locus_tag="3A_10"
                     /conf="100.0"
                     /ID="3A_10"
                     /cscore="44.62"
                     /partial=0
                     /tscore="3.07"
                     /gc_cont="0.429"
                     /rbs_spacer="5-10bp"
                     /start_type="ATG"
                     /score=64.9
                     /codon_start=1
     CDS             complement(4438..4770)
                     /rbs_motif="AGGAG"
                     /uscore="5.99"
                     /rscore="11.88"
                     /sscore="20.28"
                     /locus_tag="3A_10"
                     /conf="100.0"
                     /ID="3A_10"
                     /cscore="44.62"
                     /partial=0
                     /tscore="3.07"
                     /gc_cont="0.429"
                     /rbs_spacer="5-10bp"
                     /product="hypothetical protein X839_01085"
                     /start_type="ATG"
                     /score=64.9
                     /codon_start=1
     gene            complement(4951..5823)
                     /rbs_motif="None"
                     /uscore="-3.3"
                     /rscore="-3.27"
                     /sscore="-4.15"
                     /locus_tag="3A_11"
                     /conf="100.0"
                     /ID="3A_11"
                     /cscore="84.66"
                     /partial=0
                     /tscore="3.07"
                     /gc_cont="0.431"
                     /rbs_spacer="None"
                     /start_type="ATG"
                     /score=80.5
                     /codon_start=1
     CDS             complement(4951..5823)
                     /rbs_motif="None"
                     /uscore="-3.3"
                     /rscore="-3.27"
                     /sscore="-4.15"
                     /locus_tag="3A_11"
                     /conf="100.0"
                     /ID="3A_11"
                     /cscore="84.66"
                     /partial=0
                     /tscore="3.07"
                     /gc_cont="0.431"
                     /rbs_spacer="None"
                     /product="RNA pseudouridylate synthase"
                     /start_type="ATG"
                     /score=80.5
```

```
                     /codon_start=1
     gene            5889..8216
                     /rbs_motif="None"
                     /uscore="3.14"
                     /rscore="-3.27"
                     /sscore="2.94"
                     /locus_tag="3A_12"
                     /conf="99.99"
                     /ID="3A_12"
                     /cscore="394.79"
                     /partial=0
                     /tscore="3.07"
                     /gc_cont="0.427"
                     /rbs_spacer="None"
                     /start_type="ATG"
                     /score=397.7
                     /codon_start=1
     CDS             5889..8216
                     /rbs_motif="None"
                     /uscore="3.14"
                     /rscore="-3.27"
                     /sscore="2.94"
                     /locus_tag="3A_12"
                     /conf="99.99"
                     /ID="3A_12"
                     /cscore="394.79"
                     /partial=0
                     /tscore="3.07"
                     /gc_cont="0.427"
                     /rbs_spacer="None"
                     /product="penicillin-binding protein 2A"
                     /start_type="ATG"
                     /score=397.7
                     /codon_start=1
     gene            8426..8602
                     /rbs_motif="AGGAG"
                     /uscore="0.01"
                     /rscore="8.27"
                     /sscore="2.63"
                     /locus_tag="3A_13"
                     /conf="69.09"
                     /ID="3A_13"
                     /cscore="0.87"
                     /partial=0
                     /tscore="-4.99"
                     /gc_cont="0.401"
                     /rbs_spacer="5-10bp"
                     /start_type="GTG"
                     /score=3.5
                     /codon_start=1
     CDS             8426..8602
                     /rbs_motif="AGGAG"
                     /uscore="0.01"
                     /rscore="8.27"
                     /sscore="2.63"
                     /locus_tag="3A_13"
                     /conf="69.09"
                     /ID="3A_13"
                     /cscore="0.87"
                     /partial=0
                     /tscore="-4.99"
```

```
                     /gc_cont="0.401"
                     /rbs_spacer="5-10bp"
                     /product="preprotein translocase subunit SecE"
                     /start_type="GTG"
                     /score=3.5
                     /codon_start=1
     gene            8799..9338
                     /rbs_motif="AGGA"
                     /uscore="3.08"
                     /rscore="7.92"
                     /sscore="13.41"
                     /locus_tag="3A_14"
                     /conf="100.0"
                     /ID="3A_14"
                     /cscore="92.22"
                     /partial=0
                     /tscore="3.07"
                     /gc_cont="0.391"
                     /rbs_spacer="5-10bp"
                     /start_type="ATG"
                     /score=105.6
                     /codon_start=1
     CDS             8799..9338
                     /rbs_motif="AGGA"
                     /uscore="3.08"
                     /rscore="7.92"
                     /sscore="13.41"
                     /locus_tag="3A_14"
                     /conf="100.0"
                     /ID="3A_14"
                     /cscore="92.22"
                     /partial=0
                     /tscore="3.07"
                     /gc_cont="0.391"
                     /rbs_spacer="5-10bp"
                     /product="transcription antitermination protein NusG"
                     /start_type="ATG"
                     /score=105.6
                     /codon_start=1
     gene            9637..9948
                     /rbs_motif="None"
                     /uscore="-3.71"
                     /rscore="-3.27"
                     /sscore="-4.56"
                     /locus_tag="3A_15"
                     /conf="95.19"
                     /ID="3A_15"
                     /cscore="17.54"
                     /partial=0
                     /tscore="3.07"
                     /gc_cont="0.442"
                     /rbs_spacer="None"
                     /start_type="ATG"
                     /score=13.0
                     /codon_start=1
     CDS             9637..9948
                     /rbs_motif="None"
                     /uscore="-3.71"
                     /rscore="-3.27"
                     /sscore="-4.56"
                     /locus_tag="3A_15"
```

```
                     /conf="95.19"
                     /ID="3A_15"
                     /cscore="17.54"
                     /partial=0
                     /tscore="3.07"
                     /gc_cont="0.442"
                     /rbs_spacer="None"
                     /product="phosphoesterase"
                     /start_type="ATG"
                     /score=13.0
                     /codon_start=1
     gene            complement(10080..10376)
                     /rbs_motif="None"
                     /uscore="-5.55"
                     /rscore="-3.27"
                     /sscore="-5.75"
                     /locus_tag="3A_16"
                     /conf="99.93"
                     /ID="3A_16"
                     /cscore="37.09"
                     /partial=0
                     /tscore="3.07"
                     /gc_cont="0.407"
                     /rbs_spacer="None"
                     /start_type="ATG"
                     /score=31.3
                     /codon_start=1
     CDS             complement(10080..10376)
                     /rbs_motif="None"
                     /uscore="-5.55"
                     /rscore="-3.27"
                     /sscore="-5.75"
                     /locus_tag="3A_16"
                     /conf="99.93"
                     /ID="3A_16"
                     /cscore="37.09"
                     /partial=0
                     /tscore="3.07"
                     /gc_cont="0.407"
                     /rbs_spacer="None"
                     /product="haloacid dehalogenase"
                     /start_type="ATG"
                     /score=31.3
                     /codon_start=1
     gene            complement(10336..10575)
                     /rbs_motif="None"
                     /uscore="2.17"
                     /rscore="-3.45"
                     /sscore="-4.94"
                     /locus_tag="3A_17"
                     /conf="77.97"
                     /ID="3A_17"
                     /cscore="10.44"
                     /partial=0
                     /tscore="-3.67"
                     /gc_cont="0.408"
                     /rbs_spacer="None"
                     /start_type="GTG"
                     /score=5.5
                     /codon_start=1
     CDS             complement(10336..10575)
```

```
                     /rbs_motif="None"
                     /uscore="2.17"
                     /rscore="-3.45"
                     /sscore="-4.94"
                     /locus_tag="3A_17"
                     /conf="77.97"
                     /ID="3A_17"
                     /cscore="10.44"
                     /partial=0
                     /tscore="-3.67"
                     /gc_cont="0.408"
                     /rbs_spacer="None"
                     /product="hypothetical protein Y018_01260"
                     /start_type="GTG"
                     /score=5.5
                     /codon_start=1
     gene            complement(10674..10910)
                     /rbs_motif="None"
                     /uscore="1.15"
                     /rscore="-3.49"
                     /sscore="0.53"
                     /locus_tag="3A_18"
                     /conf="98.04"
                     /ID="3A_18"
                     /cscore="16.5"
                     /partial=0
                     /tscore="2.87"
                     /gc_cont="0.401"
                     /rbs_spacer="None"
                     /start_type="ATG"
                     /score=17.0
                     /codon_start=1
     CDS             complement(10674..10910)
                     /rbs_motif="None"
                     /uscore="1.15"
                     /rscore="-3.49"
                     /sscore="0.53"
                     /locus_tag="3A_18"
                     /conf="98.04"
                     /ID="3A_18"
                     /cscore="16.5"
                     /partial=0
                     /tscore="2.87"
                     /gc_cont="0.401"
                     /rbs_spacer="None"
                     /product="haloacid dehalogenase"
                     /start_type="ATG"
                     /score=17.0
                     /codon_start=1
     gene            complement(10885..11343)
                     /rbs_motif="None"
                     /uscore="-2.29"
                     /rscore="-3.27"
                     /sscore="4.35"
                     /locus_tag="3A_19"
                     /conf="99.99"
                     /ID="3A_19"
                     /cscore="34.83"
                     /partial=0
                     /tscore="3.07"
                     /gc_cont="0.379"
```

```
                     /rbs_spacer="None"
                     /start_type="ATG"
                     /score=39.2
                     /codon_start=1
     CDS             complement(10885..11343)
                     /rbs_motif="None"
                     /uscore="-2.29"
                     /rscore="-3.27"
                     /sscore="4.35"
                     /locus_tag="3A_19"
                     /conf="99.99"
                     /ID="3A_19"
                     /cscore="34.83"
                     /partial=0
                     /tscore="3.07"
                     /gc_cont="0.379"
                     /rbs_spacer="None"
                     /product="cytochrome C"
                     /start_type="ATG"
                     /score=39.2
                     /codon_start=1
     gene            complement(11343..11651)
                     /rbs_motif="AGxAGG/AGGxGG"
                     /uscore="4.39"
                     /rscore="3.21"
                     /sscore="9.52"
                     /locus_tag="3A_20"
                     /conf="84.17"
                     /ID="3A_20"
                     /cscore="-2.25"
                     /partial=0
                     /tscore="3.07"
                     /gc_cont="0.398"
                     /rbs_spacer="5-10bp"
                     /start_type="ATG"
                     /score=7.3
                     /codon_start=1
     CDS             complement(11343..11651)
                     /rbs_motif="AGxAGG/AGGxGG"
                     /uscore="4.39"
                     /rscore="3.21"
                     /sscore="9.52"
                     /locus_tag="3A_20"
                     /conf="84.17"
                     /ID="3A_20"
                     /cscore="-2.25"
                     /partial=0
                     /tscore="3.07"
                     /gc_cont="0.398"
                     /rbs_spacer="5-10bp"
                     /product="hypothetical protein Y018_01275"
                     /start_type="ATG"
                     /score=7.3
                     /codon_start=1
     gene            11825..14326
                     /rbs_motif="GGAG/GAGG"
                     /uscore="6.25"
                     /rscore="6.9"
                     /sscore="16.22"
                     /locus_tag="3A_21"
                     /conf="99.99"
```

```
                     /ID="3A_21"
                     /cscore="477.58"
                     /partial=0
                     /tscore="3.07"
                     /gc_cont="0.457"
                     /rbs_spacer="5-10bp"
                     /start_type="ATG"
                     /score=493.8
                     /codon_start=1
     CDS             11825..14326
                     /rbs_motif="GGAG/GAGG"
                     /uscore="6.25"
                     /rscore="6.9"
                     /sscore="16.22"
                     /locus_tag="3A_21"
                     /conf="99.99"
                     /ID="3A_21"
                     /cscore="477.58"
                     /partial=0
                     /tscore="3.07"
                     /gc_cont="0.457"
                     /rbs_spacer="5-10bp"
                     /product="leucyl-tRNA synthase"
                     /start_type="ATG"
                     /score=493.8
                     /codon_start=1
     gene            14668..14895
                     /rbs_motif="GGA/GAG/AGG"
                     /uscore="0.79"
                     /rscore="0.82"
                     /sscore="-2.91"
                     /locus_tag="3A_22"
                     /conf="91.12"
                     /ID="3A_22"
                     /cscore="13.04"
                     /partial=0
                     /tscore="-3.86"
                     /gc_cont="0.311"
                     /rbs_spacer="5-10bp"
                     /start_type="GTG"
                     /score=10.1
                     /codon_start=1
     CDS             14668..14895
                     /rbs_motif="GGA/GAG/AGG"
                     /uscore="0.79"
                     /rscore="0.82"
                     /sscore="-2.91"
                     /locus_tag="3A_22"
                     /conf="91.12"
                     /ID="3A_22"
                     /cscore="13.04"
                     /partial=0
                     /tscore="-3.86"
                     /gc_cont="0.311"
                     /rbs_spacer="5-10bp"
                     /product="transcriptional regulator"
                     /start_type="GTG"
                     /score=10.1
                     /codon_start=1
     gene            15025..15117
                     /rbs_motif="GGAG/GAGG"
```

```
                     /uscore="0.37"
                     /rscore="2.49"
                     /sscore="3.96"
                     /locus_tag="3A_23"
                     /conf="89.21"
                     /ID="3A_23"
                     /cscore="5.22"
                     /partial=0
                     /tscore="1.1"
                     /gc_cont="0.301"
                     /rbs_spacer="5-10bp"
                     /start_type="ATG"
                     /score=9.2
                     /codon_start=1
     CDS             15025..15117
                     /rbs_motif="GGAG/GAGG"
                     /uscore="0.37"
                     /rscore="2.49"
                     /sscore="3.96"
                     /locus_tag="3A_23"
                     /conf="89.21"
                     /ID="3A_23"
                     /cscore="5.22"
                     /partial=0
                     /tscore="1.1"
                     /gc_cont="0.301"
                     /rbs_spacer="5-10bp"
                     /product="hypothetical protein Y018_01295"
                     /start_type="ATG"
                     /score=9.2
                     /codon_start=1
     gene            15127..15549
                     /rbs_motif="GGA/GAG/AGG"
                     /uscore="1.07"
                     /rscore="0.91"
                     /sscore="6.23"
                     /locus_tag="3A_24"
                     /conf="99.56"
                     /ID="3A_24"
                     /cscore="17.31"
                     /partial=0
                     /tscore="3.07"
                     /gc_cont="0.338"
                     /rbs_spacer="5-10bp"
                     /start_type="ATG"
                     /score=23.5
                     /codon_start=1
     CDS             15127..15549
                     /rbs_motif="GGA/GAG/AGG"
                     /uscore="1.07"
                     /rscore="0.91"
                     /sscore="6.23"
                     /locus_tag="3A_24"
                     /conf="99.56"
                     /ID="3A_24"
                     /cscore="17.31"
                     /partial=0
                     /tscore="3.07"
                     /gc_cont="0.338"
                     /rbs_spacer="5-10bp"
                     /product="acyl-CoA thioesterase"
```

```
                     /start_type="ATG"
                     /score=23.5
                     /codon_start=1
     gene            15500..16123
                     /rbs_motif="GGAG/GAGG"
                     /uscore="-0.87"
                     /rscore="6.9"
                     /sscore="9.1"
                     /locus_tag="3A_25"
                     /conf="99.91"
                     /ID="3A_25"
                     /cscore="21.4"
                     /partial=0
                     /tscore="3.07"
                     /gc_cont="0.362"
                     /rbs_spacer="5-10bp"
                     /start_type="ATG"
                     /score=30.5
                     /codon_start=1
     CDS             15500..16123
                     /rbs_motif="GGAG/GAGG"
                     /uscore="-0.87"
                     /rscore="6.9"
                     /sscore="9.1"
                     /locus_tag="3A_25"
                     /conf="99.91"
                     /ID="3A_25"
                     /cscore="21.4"
                     /partial=0
                     /tscore="3.07"
                     /gc_cont="0.362"
                     /rbs_spacer="5-10bp"
                     /product="acyl-CoA thioesterase"
                     /start_type="ATG"
                     /score=30.5
                     /codon_start=1
     gene            16553..18007
                     /rbs_motif="GGAGG"
                     /uscore="4.71"
                     /rscore="14.48"
                     /sscore="14.89"
                     /locus_tag="3A_26"
                     /conf="99.99"
                     /ID="3A_26"
                     /cscore="260.46"
                     /partial=0
                     /tscore="-3.65"
                     /gc_cont="0.424"
                     /rbs_spacer="5-10bp"
                     /start_type="TTG"
                     /score=275.4
                     /codon_start=1
     CDS             16553..18007
                     /rbs_motif="GGAGG"
                     /uscore="4.71"
                     /rscore="14.48"
                     /sscore="14.89"
                     /locus_tag="3A_26"
                     /conf="99.99"
                     /ID="3A_26"
                     /cscore="260.46"
```

```
                     /partial=0
                     /tscore="-3.65"
                     /gc_cont="0.424"
                     /rbs_spacer="5-10bp"
                     /product="nicotinate phosphoribosyltransferase"
                     /start_type="TTG"
                     /score=275.4
                     /codon_start=1
     gene            18019..18840
                     /rbs_motif="AGGAG"
                     /uscore="-0.2"
                     /rscore="11.88"
                     /sscore="15.89"
                     /locus_tag="3A_27"
                     /conf="100.0"
                     /ID="3A_27"
                     /cscore="152.94"
                     /partial=0
                     /tscore="3.07"
                     /gc_cont="0.428"
                     /rbs_spacer="5-10bp"
                     /start_type="ATG"
                     /score=168.8
                     /codon_start=1
     CDS             18019..18840
                     /rbs_motif="AGGAG"
                     /uscore="-0.2"
                     /rscore="11.88"
                     /sscore="15.89"
                     /locus_tag="3A_27"
                     /conf="100.0"
                     /ID="3A_27"
                     /cscore="152.94"
                     /partial=0
                     /tscore="3.07"
                     /gc_cont="0.428"
                     /rbs_spacer="5-10bp"
                     /product="NAD synthetase"
                     /start_type="ATG"
                     /score=168.8
                     /codon_start=1
     gene            18856..19296
                     /rbs_motif="GGA/GAG/AGG"
                     /uscore="4.04"
                     /rscore="0.91"
                     /sscore="9.13"
                     /locus_tag="3A_28"
                     /conf="99.99"
                     /ID="3A_28"
                     /cscore="32.27"
                     /partial=0
                     /tscore="3.07"
                     /gc_cont="0.365"
                     /rbs_spacer="5-10bp"
                     /start_type="ATG"
                     /score=41.4
                     /codon_start=1
     CDS             18856..19296
                     /rbs_motif="GGA/GAG/AGG"
                     /uscore="4.04"
                     /rscore="0.91"
```

```
                     /sscore="9.13"
                     /locus_tag="3A_28"
                     /conf="99.99"
                     /ID="3A_28"
                     /cscore="32.27"
                     /partial=0
                     /tscore="3.07"
                     /gc_cont="0.365"
                     /rbs_spacer="5-10bp"
                     /product="membrane protein"
                     /start_type="ATG"
                     /score=41.4
                     /codon_start=1
     gene            19398..20735
                     /rbs_motif="GGAGG"
                     /uscore="0.57"
                     /rscore="14.48"
                     /sscore="18.11"
                     /locus_tag="3A_29"
                     /conf="99.99"
                     /ID="3A_29"
                     /cscore="240.78"
                     /partial=0
                     /tscore="3.07"
                     /gc_cont="0.407"
                     /rbs_spacer="5-10bp"
                     /start_type="ATG"
                     /score=258.9
                     /codon_start=1
     CDS             19398..20735
                     /rbs_motif="GGAGG"
                     /uscore="0.57"
                     /rscore="14.48"
                     /sscore="18.11"
                     /locus_tag="3A_29"
                     /conf="99.99"
                     /ID="3A_29"
                     /cscore="240.78"
                     /partial=0
                     /tscore="3.07"
                     /gc_cont="0.407"
                     /rbs_spacer="5-10bp"
                     /product="aminopeptidase C"
                     /start_type="ATG"
                     /score=258.9
                     /codon_start=1
     gene            20865..22193
                     /rbs_motif="GGAG/GAGG"
                     /uscore="4.62"
                     /rscore="6.9"
                     /sscore="7.88"
                     /locus_tag="3A_30"
                     /conf="100.0"
                     /ID="3A_30"
                     /cscore="166.14"
                     /partial=0
                     /tscore="-3.65"
                     /gc_cont="0.413"
                     /rbs_spacer="5-10bp"
                     /start_type="TTG"
                     /score=174.0
```

```
                     /codon_start=1
     CDS             20865..22193
                     /rbs_motif="GGAG/GAGG"
                     /uscore="4.62"
                     /rscore="6.9"
                     /sscore="7.88"
                     /locus_tag="3A_30"
                     /conf="100.0"
                     /ID="3A_30"
                     /cscore="166.14"
                     /partial=0
                     /tscore="-3.65"
                     /gc_cont="0.413"
                     /rbs_spacer="5-10bp"
                     /product="transposase"
                     /start_type="TTG"
                     /score=174.0
                     /codon_start=1
     gene            complement(22269..24599)
                     /rbs_motif="AGxAGG/AGGxGG"
                     /uscore="2.45"
                     /rscore="3.21"
                     /sscore="10.0"
                     /locus_tag="3A_31"
                     /conf="99.99"
                     /ID="3A_31"
                     /cscore="400.21"
                     /partial=0
                     /tscore="3.07"
                     /gc_cont="0.395"
                     /rbs_spacer="5-10bp"
                     /start_type="ATG"
                     /score=410.2
                     /codon_start=1
     CDS             complement(22269..24599)
                     /rbs_motif="AGxAGG/AGGxGG"
                     /uscore="2.45"
                     /rscore="3.21"
                     /sscore="10.0"
                     /locus_tag="3A_31"
                     /conf="99.99"
                     /ID="3A_31"
                     /cscore="400.21"
                     /partial=0
                     /tscore="3.07"
                     /gc_cont="0.395"
                     /rbs_spacer="5-10bp"
                     /product="penicillin-binding protein 1A"
                     /start_type="ATG"
                     /score=410.2
                     /codon_start=1
     gene            complement(24599..25108)
                     /rbs_motif="None"
                     /uscore="0.57"
                     /rscore="-3.27"
                     /sscore="-0.28"
                     /locus_tag="3A_32"
                     /conf="100.0"
                     /ID="3A_32"
                     /cscore="52.95"
                     /partial=0
```

```
                     /tscore="3.07"
                     /gc_cont="0.38"
                     /rbs_spacer="None"
                     /start_type="ATG"
                     /score=52.7
                     /codon_start=1
     CDS             complement(24599..25108)
                     /rbs_motif="None"
                     /uscore="0.57"
                     /rscore="-3.27"
                     /sscore="-0.28"
                     /locus_tag="3A_32"
                     /conf="100.0"
                     /ID="3A_32"
                     /cscore="52.95"
                     /partial=0
                     /tscore="3.07"
                     /gc_cont="0.38"
                     /rbs_spacer="None"
                     /product="Holliday junction resolvase"
                     /start_type="ATG"
                     /score=52.7
                     /codon_start=1
     gene            25273..25395
                     /rbs_motif="GGAG/GAGG"
                     /uscore="-3.72"
                     /rscore="3.31"
                     /sscore="1.07"
                     /locus_tag="3A_33"
                     /conf="86.94"
                     /ID="3A_33"
                     /cscore="7.18"
                     /partial=0
                     /tscore="1.47"
                     /gc_cont="0.333"
                     /rbs_spacer="5-10bp"
                     /start_type="ATG"
                     /score=8.2
                     /codon_start=1
     CDS             25273..25395
                     /rbs_motif="GGAG/GAGG"
                     /uscore="-3.72"
                     /rscore="3.31"
                     /sscore="1.07"
                     /locus_tag="3A_33"
                     /conf="86.94"
                     /ID="3A_33"
                     /cscore="7.18"
                     /partial=0
                     /tscore="1.47"
                     /gc_cont="0.333"
                     /rbs_spacer="5-10bp"
                     /product="hypothetical protein"
                     /start_type="ATG"
                     /score=8.2
                     /codon_start=1
     gene            25409..25789
                     /rbs_motif="GGA/GAG/AGG"
                     /uscore="-2.94"
                     /rscore="0.91"
                     /sscore="2.17"
```

```
                     /locus_tag="3A_34"
                     /conf="99.85"
                     /ID="3A_34"
                     /cscore="26.04"
                     /partial=0
                     /tscore="3.07"
                     /gc_cont="0.352"
                     /rbs_spacer="5-10bp"
                     /start_type="ATG"
                     /score=28.2
                     /codon_start=1
     CDS             25409..25789
                     /rbs_motif="GGA/GAG/AGG"
                     /uscore="-2.94"
                     /rscore="0.91"
                     /sscore="2.17"
                     /locus_tag="3A_34"
                     /conf="99.85"
                     /ID="3A_34"
                     /cscore="26.04"
                     /partial=0
                     /tscore="3.07"
                     /gc_cont="0.352"
                     /rbs_spacer="5-10bp"
                     /product="hypothetical protein"
                     /start_type="ATG"
                     /score=28.2
                     /codon_start=1
     gene            25898..26230
                     /rbs_motif="GGAG/GAGG"
                     /uscore="6.37"
                     /rscore="6.9"
                     /sscore="16.34"
                     /locus_tag="3A_35"
                     /conf="100.0"
                     /ID="3A_35"
                     /cscore="49.4"
                     /partial=0
                     /tscore="3.07"
                     /gc_cont="0.384"
                     /rbs_spacer="5-10bp"
                     /start_type="ATG"
                     /score=65.7
                     /codon_start=1
     CDS             25898..26230
                     /rbs_motif="GGAG/GAGG"
                     /uscore="6.37"
                     /rscore="6.9"
                     /sscore="16.34"
                     /locus_tag="3A_35"
                     /conf="100.0"
                     /ID="3A_35"
                     /cscore="49.4"
                     /partial=0
                     /tscore="3.07"
                     /gc_cont="0.384"
                     /rbs_spacer="5-10bp"
                     /product="cell division protein GpsB"
                     /start_type="ATG"
                     /score=65.7
                     /codon_start=1
```

```
gene            26763..27929
                /rbs_motif="AGGA"
                /uscore="3.86"
                /rscore="7.92"
                /sscore="14.19"
                /locus_tag="3A_36"
                /conf="99.99"
                /ID="3A_36"
                /cscore="197.17"
                /partial=0
                /tscore="3.07"
                /gc_cont="0.404"
                /rbs_spacer="5-10bp"
                /start_type="ATG"
                /score=211.4
                /codon_start=1
CDS             26763..27929
                /rbs_motif="AGGA"
                /uscore="3.86"
                /rscore="7.92"
                /sscore="14.19"
                /locus_tag="3A_36"
                /conf="99.99"
                /ID="3A_36"
                /cscore="197.17"
                /partial=0
                /tscore="3.07"
                /gc_cont="0.404"
                /rbs_spacer="5-10bp"
                /product="RNA methyltransferase"
                /start_type="ATG"
                /score=211.4
                /codon_start=1
gene            27932..29794
                /rbs_motif="GGA/GAG/AGG"
                /uscore="-2.12"
                /rscore="0.91"
                /sscore="-3.43"
                /locus_tag="3A_37"
                /conf="99.99"
                /ID="3A_37"
                /cscore="358.16"
                /partial=0
                /tscore="-3.48"
                /gc_cont="0.404"
                /rbs_spacer="5-10bp"
                /start_type="GTG"
                /score=354.7
                /codon_start=1
CDS             27932..29794
                /rbs_motif="GGA/GAG/AGG"
                /uscore="-2.12"
                /rscore="0.91"
                /sscore="-3.43"
                /locus_tag="3A_37"
                /conf="99.99"
                /ID="3A_37"
                /cscore="358.16"
                /partial=0
                /tscore="-3.48"
                /gc_cont="0.404"
```

```
                     /rbs_spacer="5-10bp"
                     /product="hypothetical protein Y018_01370"
                     /start_type="GTG"
                     /score=354.7
                     /codon_start=1
     gene            29896..33000
                     /rbs_motif="AGxAGG/AGGxGG"
                     /uscore="3.06"
                     /rscore="8.45"
                     /sscore="8.03"
                     /locus_tag="3A_38"
                     /conf="100.0"
                     /ID="3A_38"
                     /cscore="102.54"
                     /partial=0
                     /tscore="-3.48"
                     /gc_cont="0.409"
                     /rbs_spacer="11-12bp"
                     /start_type="GTG"
                     /score=110.6
                     /codon_start=1
     CDS             29896..33000
                     /rbs_motif="AGxAGG/AGGxGG"
                     /uscore="3.06"
                     /rscore="8.45"
                     /sscore="8.03"
                     /locus_tag="3A_38"
                     /conf="100.0"
                     /ID="3A_38"
                     /cscore="102.54"
                     /partial=0
                     /tscore="-3.48"
                     /gc_cont="0.409"
                     /rbs_spacer="11-12bp"
                     /product="RNA helicase"
                     /start_type="GTG"
                     /score=110.6
                     /codon_start=1
     gene            33156..33872
                     /rbs_motif="AGGA"
                     /uscore="4.93"
                     /rscore="7.92"
                     /sscore="15.92"
                     /locus_tag="3A_39"
                     /conf="100.0"
                     /ID="3A_39"
                     /cscore="107.82"
                     /partial=0
                     /tscore="3.07"
                     /gc_cont="0.407"
                     /rbs_spacer="5-10bp"
                     /start_type="ATG"
                     /score=123.7
                     /codon_start=1
     CDS             33156..33872
                     /rbs_motif="AGGA"
                     /uscore="4.93"
                     /rscore="7.92"
                     /sscore="15.92"
                     /locus_tag="3A_39"
                     /conf="100.0"
```

```
                     /ID="3A_39"
                     /cscore="107.82"
                     /partial=0
                     /tscore="3.07"
                     /gc_cont="0.407"
                     /rbs_spacer="5-10bp"
                     /product="hypothetical protein"
                     /start_type="ATG"
                     /score=123.7
                     /codon_start=1
     gene            33899..35227
                     /rbs_motif="AGGAG"
                     /uscore="2.98"
                     /rscore="11.88"
                     /sscore="18.92"
                     /locus_tag="3A_40"
                     /conf="99.99"
                     /ID="3A_40"
                     /cscore="240.43"
                     /partial=0
                     /tscore="3.07"
                     /gc_cont="0.4"
                     /rbs_spacer="5-10bp"
                     /start_type="ATG"
                     /score=259.4
                     /codon_start=1
     CDS             33899..35227
                     /rbs_motif="AGGAG"
                     /uscore="2.98"
                     /rscore="11.88"
                     /sscore="18.92"
                     /locus_tag="3A_40"
                     /conf="99.99"
                     /ID="3A_40"
                     /cscore="240.43"
                     /partial=0
                     /tscore="3.07"
                     /gc_cont="0.4"
                     /rbs_spacer="5-10bp"
                     /product="UDP-N-acetylmuramate--alanine ligase"
                     /start_type="ATG"
                     /score=259.4
                     /codon_start=1
     gene            35313..35780
                     /rbs_motif="None"
                     /uscore="0.05"
                     /rscore="-3.27"
                     /sscore="-0.15"
                     /locus_tag="3A_41"
                     /conf="100.0"
                     /ID="3A_41"
                     /cscore="49.47"
                     /partial=0
                     /tscore="3.07"
                     /gc_cont="0.404"
                     /rbs_spacer="None"
                     /start_type="ATG"
                     /score=49.3
                     /codon_start=1
     CDS             35313..35780
                     /rbs_motif="None"
```

```
                     /uscore="0.05"
                     /rscore="-3.27"
                     /sscore="-0.15"
                     /locus_tag="3A_41"
                     /conf="100.0"
                     /ID="3A_41"
                     /cscore="49.47"
                     /partial=0
                     /tscore="3.07"
                     /gc_cont="0.404"
                     /rbs_spacer="None"
                     /product="GNAT family acetyltransferase"
                     /start_type="ATG"
                     /score=49.3
                     /codon_start=1
     gene            35880..37856
                     /rbs_motif="GGA/GAG/AGG"
                     /uscore="-2.51"
                     /rscore="-1.46"
                     /sscore="0.31"
                     /locus_tag="3A_42"
                     /conf="99.99"
                     /ID="3A_42"
                     /cscore="247.34"
                     /partial=0
                     /tscore="3.07"
                     /gc_cont="0.375"
                     /rbs_spacer="11-12bp"
                     /start_type="ATG"
                     /score=247.7
                     /codon_start=1
     CDS             35880..37856
                     /rbs_motif="GGA/GAG/AGG"
                     /uscore="-2.51"
                     /rscore="-1.46"
                     /sscore="0.31"
                     /locus_tag="3A_42"
                     /conf="99.99"
                     /ID="3A_42"
                     /cscore="247.34"
                     /partial=0
                     /tscore="3.07"
                     /gc_cont="0.375"
                     /rbs_spacer="11-12bp"
                     /product="aminodeoxychorismate lyase"
                     /start_type="ATG"
                     /score=247.7
                     /codon_start=1
     gene            38000..38386
                     /rbs_motif="None"
                     /uscore="-0.38"
                     /rscore="-3.27"
                     /sscore="-7.29"
                     /locus_tag="3A_43"
                     /conf="100.0"
                     /ID="3A_43"
                     /cscore="77.3"
                     /partial=1
                     /tscore="-3.65"
                     /gc_cont="0.442"
                     /rbs_spacer="None"
```

```
                     /start_type="TTG"
                     /score=70.0
                     /codon_start=1
     CDS             38000..38386
                     /rbs_motif="None"
                     /uscore="-0.38"
                     /rscore="-3.27"
                     /sscore="-7.29"
                     /locus_tag="3A_43"
                     /conf="100.0"
                     /ID="3A_43"
                     /cscore="77.3"
                     /partial=1
                     /tscore="-3.65"
                     /gc_cont="0.442"
                     /rbs_spacer="None"
                     /product="transcription elongation factor GreA"
                     /start_type="TTG"
                     /score=70.0
                     /codon_start=1
BASE COUNT    11792 a   7062 c   8213 g  11319 t
ORIGIN
        1 gaaacagcta tgaccatgat tacgccaagc tatttaggtg agactataga atactcaagc
       61 ttgcatgcct gcaggtcgac tctagaggat cccacttact ggggaattga ttgcacctag
      121 tgttgcagta ggcgataagg tccttgttga aaatggtaca ggtgtcagtg ttaaagacgg
      181 agaagattcc gtttcaatta tccgtgaagc agatatttta gcagtcctag cttaaattct
      241 tgctaatcag atataaatta agataaagaa ggagtgagta attatggcaa aagatattaa
      301 attttcatca gatgcacgtg cagcaatggt tcgtggtgtt gatactttag ctgataccgt
      361 taaggtaacg cttggcccta aaggtcgtaa tgttgttttg gaaaaggctt ttggttcacc
      421 acttatcact aatgatggtg tgacaattgc taaagaaatt gaacttgaag atcattttga
      481 aaatatgggt gccaagctcg tcagcgaagt ggcttcaaaa actaatgata ttgccggtga
      541 cggaacaact acagccactg ttttgacaca agctattgtt cgtgaggggc taaaaaatgt
      601 aacagctggt gctaatccaa tcggtatccg tcgtgggatt gaagcagctg tagctgcagc
      661 tgtggaagag cttaaagtca ttgctcaacc agttgctaat aaggaagcta ttgctcaagt
      721 tgctgcggtt tcatctcgct ctgaaaaagt tggtgaatac atttcagaag caatggaacg
      781 cgttggtaat gatggtgtta tcactattga agagtctcgt ggtatggaga cagaactcga
      841 agtggttgaa ggtatgcaat ttgaccgtgg atatctttct cagtacatgg ttactgataa
      901 tgaaaaaatg gtcgcagatc ttgaaaatcc atatattctt gtgactgaca agaaaatttc
      961 aaatatccaa gatattcttc cactcttgga agaagtgctt aagaccagtc gtccactttt
     1021 gattattgct gacgacgtgg ctggtgaagc tcttccaaca cttgttctta acaaaattcg
     1081 tggaaccttc aatgtcgttg ctgttaaggc gcctggcttt ggtgaccgtc gtaaagcgat
     1141 gttggaagac attgctgtat tgactggagc aactgttatt actgaggatc ttggtcttga
     1201 acttaaggat gcaactatgg aatcacttgg tcaggcttcg aaagtaactg tggataagga
     1261 tagtacagtt atcgttgaag gagcaggaag cgcagaagca attgctaacc gtgttaacct
     1321 cattaaatct caattggaaa caacaacatc tgaatttgac cgtgaaaaac ttcaagagcg
     1381 tttggctaaa ttgtcaggtg gtgttgcagt tattaaggtt ggtgcagcca cagaaacagc
     1441 tcttaaagaa atgaaacttc gtatcgaaga tgctctcaat gctactcgtg ccgctgttga
     1501 agaaggtata gtagctggtg gtggtacagc cctagttaac gtcattgcta aggttgctga
     1561 acttgatctt gaaggtgacg atgctacagg acgtaatatt gtcctccgtg ccttggaaga
     1621 acctgttcgt caaattgctt ataatgccgg ttatgaaggt tcagttatca ttgataaatt
     1681 gaagaatagc ccagttggta caggatttaa cgctgccaat ggcgaatggg tagatatggt
     1741 tgaatcagga atcattgacc cagttaaggt aacacgttca gcccttcaaa atgctgcttc
     1801 agtagctagt cttatcttga caacagaagc agtcgttgcc gacaaacctg aacaaaaggc
     1861 tccagcagcg ccagctatgg atccaggaat gatgggttac taagatttaa gaaagaaaaa
     1921 aacctaggag aagtcgagaa ataaactgca ccccaaaagt tagacaaaaa aatctacttt
     1981 tggggtgttt ttattatgaa actaacttat gaagataaag ttcaaatata tgaattgaga
     2041 aatcaaggtc aaaccttcaa ccaactttca aaacgatttg gtgtggatgc ttctggatta
     2101 agatatatga cgagattgat tgaacgttac ggaatagaaa ttatcaagaa aggtaagaat
     2161 cgttactatt caccagaatt aaaacaagaa atgattgata aagtgctact tgaagattgt
     2221 tctcaacaaa gtgtttgcct ggactatggc ctctcaaatt aagggttgct taacaattag
     2281 atagcacaat acaagaaaaa tgggtatact tttcttgaga aaagaagagg gagagtacct
```

2341 aaaatgggga gtaagccaaa gaagaaacct gaagagatga cagagttaga acgtcttcaa
2401 gcagaaaatg agtacctgag agtggagaat gctattctaa aaaagttgag agaactccga
2461 ttgagggacg aaagggagca agaagaaaaa cagaaattgt tcaaggatta gtaacagagt
2521 ttcctttaga tatccttcta aacattatca aacttgctcg ttcaacctat tattagcact
2581 taaagaaact gaatcaagta gataaaaatc aatctatcaa agtggaaata caagccattt
2641 atgatgaaca taggggaaat tacggatatc gtcgaataac tcttgaattg aggaatcgtg
2701 gttttgtagt gaaccagaag aaggtccaac gtctgatgaa gctccttggt ttaagttccc
2761 aaattcgtcg taaacgcaag tattcttcat accaaggaga agttggcaag aaagcagatg
2821 accttagtga ccaaggttga caataccaac atcagtatta tcatcaattt ttagaggata
2881 aagggaccca accgtccatg tcacgtaagg ggaaaaatag tccagataat ggtatgatgg
2941 agtccttctt tggcattctt aagtctgaga tattttatgg ttacgagaag acgtttcact
3001 cacttgagca attggaacaa gctattgtag actatattga ttattacaac aacaaacgca
3061 ttaaagtaaa attaaaagga cttagtcctg tgcagtacag aactaaatcc ttttggataa
3121 attagttgtc taactttttg ggtcagtac aaaacttttt ctgaattttt ttttattagt
3181 tattgttaat taatgaaaag ggttaaacat atcagaactt ccttgtttac ctgacatctg
3241 catcataatt tgttgctatc tagtgcgttc tgcttcctta ttcgtattga tagaaataat
3301 aagcaagatg atctatccaa tgaaagtcca gcttaacagt attgagaaaa gaaagattag
3361 ccatttcttt tctgttttgt ttagaagtgc tggtaggatg tcaataatga ttgctggtag
3421 acccacaatc aaagtccaaa atgtcaaacc taatcccatg ctagaatcgt ctccaataac
3481 tccaagtaag aaccatccaa gagtccaaag gataactggt atggtagcga tggctatcat
3541 gatgaggttt gtttgagatt tcttttctgg cttcttccaa gcctcattaa gaagtgctgt
3601 ttgtttgtag gaagcttgca tgagcttgtt cggcaaagga atttaaaggc ttattagtag
3661 tgtcttgtat tggatttatt tgttcttgaa agttggctgt tggagcttct tgacctatgt
3721 taggagtttg gaaagtttgt ctttcatcga tactagaatc atccaaggat tcttcagtga
3781 catcataatt gtcaacatca tttttgacag tttctacagt ctctgctgat ggttcaggtt
3841 ctgtatgttc aacagtttct gagttttgcg tcttttctac agtctctttt tgtgagagtt
3901 tttttgttg ctttgacagt ttgtttggga gtttctcaga caaactcgcc tcgattggct
3961 gcttcagcca tttcttggat acttggatta cgtccgtgga ctgcttggaa aaaatcaatc
4021 caatcttgtt gtttcatata gttaaactcc atttgtttcg attataaatt ttgatgctta
4081 caattatagc aatagtgtaa gggatatgca atgggagttg gataggacag tttatttctc
4141 tataatcttt ttgtcactaa agatggaaa gagaaagaaa taggttaaga tatagatcac
4201 gagaggatat atcagtggat tatagtccca ggctccttgc caatctaggt gtaagagtct
4261 aacactagca cgtgtcagac ctgcgctcca ccccttgagt ccaaatggtt ttaggataag
4321 gttgagtggt gttgggagat gtaaaaggta gactccataa attgccatta aaactaagag
4381 atataatcta aaaaaaacgt tggtggtagg cccgcaagcg tgtgaaatag tttttcatta
4441 cccaataaca gcaaaaagtt tttgagcgtt tgattggcga gtagcatcca tgatgagaaa
4501 ccagtcaacg attgaccaga taccacaacc accacatgtc aaaagtttac cgataccaag
4561 accaacttgt cccaacataa agcggtcgat tccaagagaa ccaccaaaga gtgagatgac
4621 aagcatcatt gtaggatctt ggaagctgat tgagtaaagc agtgaaagtt gttgttcagg
4681 aagtgcctca aggcgttggc ggatgtactg cacttgttct gttgggaaat acttagcgtt
4741 aattgctagc cattgatcga ccggattcat aattaaatac tcctaaaatt ttaaattata
4801 cgtaaaggat aacataatgt aaaggagatt tcaaggtagt caaggctttc gaaatcaata
4861 aaagcaactt atttgcaagg ttccgctgtc ttataacgag aagatcatta ggataatcct
4921 ttcgcactat gtttgagagt gagtttgctt ttatgatttg tgatttctca acacttgttc
4981 aaaactttct gatttaacct taacagtgac tttttcgagt gttaaggggt gagtgaagct
5041 aagggagtgg gcgtgtagca tgagtctaga tgctggctga tgactgtaaa gtgggtcccc
5101 cagaatagca tgaccgtgat gggacagatg cacacgaatt tggtgagttc ggccggtttt
5161 tagttggcag ttgaccagac tggctccttc aaaggttttg agacgtgtga cggttgtata
5221 cgcactctgc ccttttcgag gatccaccac ccgttttcta cgatcgtggc ggtcgcgtcc
5281 aatcttgtcc ttataaacag ttttcttggt ctgaaattta ccttcagtca atgcccagta
5341 ttgacgctga atcatcttat cttctaaaag ccggttaaga attggcagga taaacggatt
5401 tttagcgaaa agcacagctc cactagtttc catatctagt cgatgaacga cgtaacaggt
5461 ttgcccacaa taagccgaaa catggttaag aagagcgatt tctgtgggtt tattgccatg
5521 cgtcttcatt ccttcaggct tattgaccac gatgatatgc gaatcctcat agagaggctt
5581 taccaaattg gcatctccca tagggatagt tttattagga tagtcctctg tgtcaaaggt
5641 caggctaatc ctatcacctt ctttaacagg actctgccaa ttaatagttt ctccattgat
5701 aaggacatgt tttttcgtgc gtaggaaatg acgaatctta cgaggaatga ggagtttggt
5761 ttctagtaat tctttgacag ttgttgcagg gtagggattg ataatttcta cgtgtaaagt
5821 catagtttca ttatatcatg aacgtttaga agaggacaga acctgtaaaa acttggtata
5881 ataggtttat gacacttttt gataatctaa aaaacaaact ttttaaagaa gaaactagcg
5941 ggaagggtaa ggaatcccag cctgctggaa aatcgaagga tttacaagat aggataaaag

6001 gtctttttgc caagaagcct gatgaagttg aacaagccgt tgaagaactt gacatgtcac
6061 gagaaagcca agagacttca cgttaccaac gttctaagca aaagaaacct attgacacga
6121 ccaaaccctt aggtaaggtt cgggctttct tgagcaagtt cactctttcg ccaagaaatc
6181 ctattcgccg tttctggcgt cgctatcata ttggtaaaat tctctttatc ttggtagccg
6241 tgttggttct gacagtaggc tcctatctct tctatatcgc taagacgacc aatgtctcag
6301 atcttcagga tgccttgaag gctactacgg ttatctatga caaagaaggc aatcaagctg
6361 gttcactgtc agggcaaaaa gggacctatg tggagctaga tgctattagt gatagcttag
6421 aaaatgccgt cattgcaacg gaggatcgta cctttttacaa aaatagtggg attaacgtca
6481 aacgtttcct cctagccatt gtttctatgg gccgatttgg tggtggttcg accatcacac
6541 aacagttggc taaaaatgcc tttttgactc aggaacagac agtaactcgt aaggccaagg
6601 aattctttct ttctttggaa ctaaccaaga aatacagcaa gcaagaaatc cttaccatgt
6661 atctcaataa tgcctacttc ggaaacggtg tttggggtgt tgaagacgcc agccaaaaat
6721 actttgggac aagtgctgcc aatctaacgg tggatgaggc agcgacactt gcaggtatgc
6781 ttaagggacc tgaaatttac aatccgattg ataatatcca aaatgcaacc aaccgccgaa
6841 acactgtcct tgccaatatg gtagatgatg agaagttgag tcaagctgat gctgatagtg
6901 cagctggagt tgatatggct agtcgcttgg ttgatactta tcaagggact ggtgatgact
6961 ataggtatcc gtcttacttt gatgctgtca ttgaagaagc tactaagact tatggcttga
7021 gtgaggatga aattgttaaa aatggctaca aaatctatac agagatggat gccaactcac
7081 aggctaatat gcagcagact tatgaaaata gttatctctt tcccacgtca gaaagtgatg
7141 gtagcacagc ccagtcagct agtgtagcac ttgatccatc aactggagcg gttcgtggtt
7201 tggtaggtcg tgtaggtggt acaggtgata caaccttccg taatttcaac tatgcaaccc
7261 aagggaagcg tagtcctggt tcgacaatca aacctttggt ggtttatgct ccagctctgg
7321 cttcagggtg gagcatcaat aaggacctac ccaatacgcc aattgattac aatggctata
7381 ccccaaccaa ctatggtggt attgagactg atgatgtgcc catgtatcaa gctttggcca
7441 actcttacaa tattccggca gtttatctct ttaaccaaat cggtatacaa aaagggatta
7501 gttacggtca gaaatttggt cttaactttg ataatgttcc agaagaatta gggattgcac
7561 tcggtggtgg tgtgacagct agtcctctac aaatggctca ggcttatgcg acctttacca
7621 atggtgggga gatgaataca gcttatttta tcactaaaat tgaaaatgct agtggtgata
7681 tcattgctac ccatagtaag aagtctaaac gtattatcag ccagtctgta gctaaccaga
7741 tgactagcat gatgctcggt accttctcaa atggttccgc tgttaatgcc aactacacgg
7801 gttacactat ggcaggtaag acaggtactg ttcaggccga gtttaacaaa gatttgacca
7861 gtgaccagtg ggtaattggc tatactcctg atgtggttat aacgacttgg attggttttg
7921 ataagactga cgagaatcac tacttaacag gagcaagctc aggaacggct tcaactattt
7981 tcagctatat cgctgcagat gttttgccaa atacaccagg taatgagttt accgttgaaa
8041 atgcctatgc ggctgatggt caaaccttag attatacagc aaatccaaac gattcacgta
8101 atagcaataa taagtcatgg acagacaagg cttcagacgt tgtcaatgat gtaaaagatc
8161 aagcaagcag cctatgggat aagctctcag actccttctc aggtctattc agatagcttg
8221 tcaattaagt ttaaaagtgg taagataaaa agaacggagg cgttatggca cagaaaaaag
8281 caagcctagc gtgtgcagat tgtgggaacc gaaactactc gattagtgtt agtagcactc
8341 ccaaaccaac acgactagaa gtgaacaagt tttgcaaaaa ttgtaaaaaa tataccttgc
8401 ataaagaaac aagataggag acaccgtgag acaaaccgga agtattttca aagtcttgaa
8461 agacacgact tggccagagc gtaaacaacg ctggcacgat tttagttcag ttcttgagta
8521 tactgcattc ttcactgtca tcatctatgc gtttgatgaa ttgttgagtc gcactatgtc
8581 agcactgatt aactttttct aaagaaaaca taaaaagagt gggcatgctt gctctttttt
8641 gttataataa atgcagataa aagcgagaac aagggcctcc aggctttttt attacgcttt
8701 gaattaaaac ataggcattc atttacattt aacgcttgcg cctcgaatat tgatggtcat
8761 gtttaaatgt catttagaac aaagaaagga atcctaaaat gctagattca ttcgacaagg
8821 gctggttcgt ccttcaaacg tattcaggat acgaaaataa agttaaagaa aatcttttgc
8881 aacgagctca aacttataac atgcttgaaa atatcttgcg tgttgaaatc ccaactcaaa
8941 ctgtcaatgt tgagaaaaac ggtaaagtta aagaagtgga agaaaaccgc ttccctggtt
9001 acgttcttgt tgagatggta atgacagacg aagcatggtt tgttgttcgt aatactccaa
9061 acgttactgg tttcgttggt tcacacggaa accgttctaa accaacacca cttttggaag
9121 aagaaatccg tcaaatcttg atttcaatgg gtcaaacagt tgatgtcttt gatacgaata
9181 tcaaggttgg tgacgtggtt caaatcatcg atggtgcctt tatggggcaa gaaggccgtg
9241 ttgtcgaaat tgaaaacaac aaggttaaaa tcatgattaa catgtttggt tcagaaacag
9301 ctgctgagct tgaactctac caaatcgctg aactttaaaa tagtcaaaaa ggttggaacc
9361 aggagcttca acctcgtaag actatctcat taaacatggg ggagagacta gaatctagat
9421 ttttaaattt agttctgtgt cactcccttt ttctaacgga tgatgaatta taaacaattt
9481 tacgataaga ttagcggtcc attgcgtcct tatgctaagg tcttgggagg attgaacaag
9541 gctataacaa ggacttttta cctgcttttt cctatatttc ttattttggt ttggctgagg
9601 catggctggt ttgttctttt tacgacagtg cttattatgg gagaaggctt ttttctgctt

9661 tctcttggtc gtagccttta taatcgccca cgcccttatc agacctggga cattcagcct
9721 cttattaaaa aggatagtct tggcaagtca tttcctagcc gtcatgtttt ttcagcaacg
9781 accattgcca tgtttacctt attgcttaat ccgtggctag gtgggactat gctttttctc
9841 gcagcaattt tggctatctt gcgcgtgcta ggtggtgttc attatcctag tgatgttttg
9901 gcaggctatg tcattggaat tcttgttggc ctccttcttt acctttagaa atttttggtc
9961 ttcttcgaga gaggagggct tttttcattg aaaaaagaaa aggacccttt ggggaccttt
10021 tctcttgttc taaaattcga gttggcgatt aaaggttgtt cccaccattc gatattggct
10081 tagtttaaaa catgctctat gatagcttgt gttactccat gttgatcatt tgaggcagtc
10141 actagatttt cagacttctt gactccctca ggtgcattga ccatagcaac tcctaggcta
10201 gccagagcga gcataggact atcgttgaaa ttatcaccaa tagtcataat ctgttcaaga
10261 ggaatgtcat aatatttggc aacctcaagg agagcatgtt ctttagaaac gtgcttggca
10321 gtgacttcta agtagttatc gttggataag tagaaggctg tctgtggaaa atccatgctt
10381 aggagttttt ggtggaggtc ctggataaac ttcaggctta tcaatcaaca atagcttgtg
10441 gatagaaggc tttgaatctg agacagtgtc ttggagttcc ttaataatag gagaatcacc
10501 cgtgatactt gcttccaact gagaccattt gtttagttgg ttggtaatcc aatccttgcc
10561 agagtagata ttcacagaaa cacttggata gtttgtggat aagtagttaa aaaattttcg
10621 agtttcagaa ttatccacag gatgttctat gatagtacga tcaccctcaa gtactaggcc
10681 ccgttgtagc aggcgatagg attgtcatga agtcctagtt tatgagcaat tggttgcatt
10741 cctagtggag aacgggcaga agctaaaaca aaaggtatct tcgccttttc aagctctagc
10801 atgacttcaa tcagttgctc atctacctgg tggttactag tgaaaatagt cccatcaata
10861 tcgctgataa tcaaacgaat tttattaata atcgctatcc tttccatcat ataaaattat
10921 tactttatct ttaagtgttt caactatatc agactcaggc tccttatctg taatccagta
10981 ttcaaagtcc tcaagtttac ccaagatata tttggaatgc ttttcatatt tgtcagattc
11041 agcaataagt atcttagttc tagtagtttg caaggctagc tgtttgacgg caacgtcttc
11101 ttcatcttca aagctaacca atccttgact gagactagcc gctccaaaga aggctaagtc
11161 aaattgtaga tgttgcaaga tctgtgcttc gtgaatcgag tagtagaagc ggtttttagt
11221 gtagaatttg cctcccaaga gctgaaaatc aatcccttcc ttaccactta ggaccaaggc
11281 attatccaag gagtgggagt aaacagtgac gttcttagtt aatttctgtg aaacttcaag
11341 catcaaggta gaaacatcga aaaaaagtag agattgttct ttgatgaagc taagcgccag
11401 gtctgccatt tttttcttat cttctgtgag tcctttctga cgttcttata agccaagaac
11461 actggtgttt ttaactggaa gaataccgca ccatgggtac gttgagccag attcctctgg
11521 ctgagacaat caaaatctct tctgacggta tatttggaaa ctcctaattc atcaaccatt
11581 tctttgacag ataggtttcc tcgctcttcc aagagcatca aaattctctc aaatcgttgt
11641 tcctgataca tgagtcacct gctttctgat attagtatag tatcttttta cgtttttttgt
11701 atataaaaat gcaaaaaaac gcaaactaag ccaggttcaa gttacctcgt aaacttctct
11761 tactatcttt aaaaatcctt caaaaatgct ataatagaag cattacgaat attggagaga
11821 aaccatgagt ttttacaatc ataaagaaat cgagcctaag tggcaaaaat actgggctga
11881 ccatcacaca tttaagacag gaacagatgc ttcaaaacct aagttttatg cgcttgacat
11941 gttcccttac ccatctggag cgggtcttca cgtaggacac ccagaaggct acacagcgac
12001 agatatcctt agccgtttca agcgtgccca aggctataac gtccttcacc caatgggatg
12061 ggatgctttt ggtttgcctg cagagcaata tgctatggat actggtaatg atccagctga
12121 ctttacagcg gaaaatattg ccaacttcaa acgtcaaatc aacgcgcttg gcttctctta
12181 cgactgggat cgtgaaatca acacaacaga tccaaactac tataagtgga cgcagtggat
12241 tttcaccaag ctttacgaaa aaggtttggc ttatgaggcg gaagtaccag taaactgggt
12301 tgaggaacta ggaacagcta tcgccaatga agaagtcctt ccggacggaa cttctgagcg
12361 tggtggctat ccagttgtcc gtaaaccaat gcgccaatgg atgcttaaaa tcactgctta
12421 tgcagagcga ttgctcaatg acttggatga gttggattgg ccagagtcta tcaaggacat
12481 gcaacgcaac tggattggta aatcaactgg tgccaatgtc accttcaaag tgaaaggtac
12541 tgacaaggaa ttcaccgtct ttacgacacg tccggatacc cttttcggtg cgaccttcac
12601 tgttttggcg cctgagcatg acttagtaga tgctatcaca agtcctgaac aagctgaggc
12661 tgtagctaac tacaaacacc aagctagcct taaatcagac ttggctcgta ccgaccttgc
12721 caaggaaaaa acagggggttt ggactggagc ttatgctata aaccctgtta acggtagaga
12781 aattccaatc tggattgcgg actacgttct cgctagctac ggaacaggtg cagttatggc
12841 cgtacctgct cacgatgagc gtgactggga atttgctaaa cagtttggtc ttccaatcgt
12901 agaagtactt gaaggtggca acgttgaaga agcagcttac acagaagacg gtcctcatgt
12961 caactctgac ttcttgaacg ggcttaacaa agaagaagct attgctaaaa ttgtagcttg
13021 gttggaagaa aaaggctttg gtcaagaaaa aatcacttac cgtctccgcg actggctctt
13081 tagccgtcaa cgttactggg gtgaaccaat cccaatcatt cattgggaag atggcacttc
13141 aacagccgtt ccagaaagcg aattgccact tgtcttgcca gtaaccaagg acatccgtcc
13201 ttcaggtact ggtgaaagtc cattggctaa cttgacagac tggcttgaag tgactcgtga
13261 agatggggtc aaaggtcgtc gtgaaaccaa caccatgcca caatgggcag gttcaagctg

13321 gtactacctt cgctatatcg acccacataa cacagaaaaa ttggcagatg aggacctcct
13381 caaacaatgg ttgccagtcg atatctacgt gggtggtgca gaacacgccg ttcttcactt
13441 gctttacgct cgtttctggc acaaattcct ctatgacctc ggtgtcgttc caactaaaga
13501 accattccaa aaactcttta accaaggaat gatcttggga acaagctacc gtgaccaccg
13561 tggtgctctt gtggcgactg acaaggttga aaaacgtgat ggttccttct tccatgtgga
13621 aacaggagaa gagttggagc aagcaccagc caagatgtct aaatccctca agaacgtggt
13681 taacccagac gatgtggtgg aacaatatgg tgctgatacc cttcgtgtct atgaaatgtt
13741 catgggacca ctcgatgctt ctatcgcttg gtcagaagaa ggcttggagg gaagtcgtaa
13801 attccttgat cgtgtttacc gtttgattac aagtaaagaa atcgttgcgg aaaataatgg
13861 tggtcttgac aaagtttata acgaaaccgt taaatctgtc actgagcaaa ttgagttgat
13921 gaaattcaac acagccattg ctcaactcat ggtctttgtt aacgcggcta acaaggaaga
13981 caaactctat gtggattatg ccaaaggctt tgtccaattg attgccccat ttgcgcctca
14041 cttggcagaa gaactctggc aaactcttac agcaacaggt gagtcaatct cttatgtggc
14101 ttggccaact tgggacgaaa gcaaattagt tgaagacgaa atcgaaatcg tcgtccaaat
14161 caaaggaaaa gtccgtgcta aactcatggt cgctaaagac ctttcacgcg aagaattgca
14221 agaagtggct ctggctgacg aaaaagtcaa agcagagatt gatggcaagg aaatcgtgaa
14281 agtaattgcg gtaccgaaca agcttgtgaa cattgttgtg aaataaccaa tttgtttgct
14341 gatttgcaac cttaaacagt tccccaaact gtttgagcta actaaactcc atttatattg
14401 ttgtgggata agaaatgaat cctctagagt tagactctgg aggatttttt actcttcttg
14461 acaagaaaac gatattttgt aataccatta acaaaaagaa ggaaactatg aataagaaag
14521 aactgcataa gtttaataaa tgttttaata gctttgcttt agcctttgaa cggttgaaaa
14581 aggaccagca taggaataag tattgataaa tcaatccaca ctattaatga agtccgtttg
14641 attgacctaa taggtcggaa tcagcctgtg aatctagtga aattatctgg gttacttgaa
14701 gtatcaagga gtgccgtcac tcagagtgtt agacgtttaa tcaagaaaaa cctcgtttct
14761 tttgaatttg cacaggataa tgaaaaaaat aaatatctga tattatctaa aaaagggtt
14821 gaggttttta acatccataa agagcagcaa gaacatattg aaaaagccat ttttttcaat
14881 tttaagaaac tatagtgagg aggaccttca aatggttatg aaactgctgg atgatgttga
14941 acaggtgtgg cgagaattac cgtggaaaaa atcacggtaa ttttaaaacc atattgttaa
15001 tcatattaac aaaaagagga agatatgaat atgcaaattg aacttatctc agcgtctgag
15061 ttagcagatg aatcttttaa tattgtcatc aatgacttaa aaccaataga aacatagcga
15121 gtcgaaatgt ttctatctga ttattattgt atcaatcctc ccatgcgttt agctcatgat
15181 gttttatgga aatcaacagc gacttttgta tcagatacga atggtattat taatatctca
15241 caaaccccat cttgttctgg ttcgtatgaa ggtatttcaa caatgggact attttttaat
15301 gccaagccct tgaccaataa gagaaagaaa cttccaaact ctctttctaa aattccctta
15361 ttagatcatt tttttgtggg aatcaaaatt atgcaaggaa atactgtcat tgcagagaga
15421 actttcacaa gacattatat gagttcgcaa attagtcatc aagatattta cgggaaacat
15481 tttcaaggga gactctttta tgataaaaag gcaatcaaaa caccaagcat tgattattgt
15541 gagcggtagt gaaggaagga ttgaaaaggc acagaatata gctcagcttc tctcttctag
15601 aggttatatt tgtttagcaa ttgcctactt tggtttagag aggttgccaa aacatttgaa
15661 acgtattcct ttagagtgcc ttgtggaggc aaagagttat ctatgtcagt accctcaagt
15721 agatagtgaa agaattggaa tctatggacg gtctaaagga gcagagttag ttttagctga
15781 ggagagcatt tttaatgacg ttcaatgctt ggtactaaat tccccctctg atgtggtgta
15841 tgaaggaata atagggaaat ggaactctca tacttcttat tggacgcatt tgcaaaaaga
15901 acttccctat caaaagtttc gatttagaga ttatttgttc agtaaacttt ttagaaaaga
15961 tttccctaaa gattgctctg ctaggataga tattggtcaa atgcattcac caatcttgtt
16021 gctcgaaagt accgttgatg aaatatggga tgcttcgtca gcaattgatg atattgtctc
16081 acattataaa ggacactaca ttacatttaa aaaaatatca tgaaacaggt cacatgttga
16141 cggtggctta tcaaccaaat catcgttatc gaaaagattg gcgtttactg atgaaagaga
16201 gtgaggattt ttggttggct acgattcatt tttttgatag gcatttgaaa aagcaataaa
16261 agaagattat aaatatgcta taatagtccc aactaagata aaaacacagt tctggttggt
16321 aagtccatac acgtagacga gcctatcagt aacctaagga tatgaataga aatgaagagt
16381 agtccatcaa ggctactctt tttcgtcaaa agaaccatta gaaaaatcgg aaaatccaaa
16441 caatatttaa ctttctgaaa tgtggagaca attgctcaaa aatgctataa tagtcagcat
16501 aaacaagcta ttaataacaa aaaataagta cctattcgat tggaggaaat ccttgtataa
16561 agatgatagt ttaaccttgc acacggactt gtatcaaatc aatatgatgc aggtctactt
16621 caaccaaggt attcacaata aaaaggccgt ttttgaagtt tatttccgtc aacttccgtt
16681 taaaaatggc tttgctgtgt tcgcaggtct ggagcatatt gtcaactatc ttgaaaatct
16741 gactttttca gaaactgata ttgcttatct gaaggattta ggctatccga aggattttct
16801 ggactatctg gccaatctaa aactcgagtt gactattaat tcagcccttg agggtgattt
16861 ggtatttgct aatgaaccga tttttcaagt ggaaggtccc ttggatcagt gtcagttagt
16921 agagactgcc ctactgaata tccttaatta ccagattctt attgcgacta aggcagctcg

16981 tattcgttct gttattgggg atgctcctct gttggaattt gggacacgtc gtgcccaaga
17041 gatggatgca gcaatatggg ggacgcgtgc agccgtgatt ggtggtgctg acgcaacttc
17101 aaatgtacgt gcaggtaaga ttttcggtat tcctgtttca ggtactcatg cccatgccct
17161 tgttcaagct tatggaaacg attatgatgc ctttaaagcc tatgcatcta ctcataaaga
17221 ctgcatattt cttgtggata cctatgatac ccttaagatt ggtgttccaa atgctatccg
17281 tgtggctaaa gagctaggtg ataagatcaa cttcttgggt gttcgtcttg attcaggtga
17341 cttggcttat ctgtctaagc aggtccgtaa gcaactagat gcggctggtt tccctgatgc
17401 caagatttac gcttcaaatg accttgatga aaataccatt cttaacttga aaatgcagaa
17461 ggccaagatt gatgtttggg gtgttggtac taatcttatc acagcctatg atcaaccagc
17521 cttgggtgcg gtctacaaaa ttgtctcaat cgagaatgat cagggagtca tgcaggatac
17581 catcaagttg tccaacaacg ctgagaaggt ttcgacacca ggtaagaagc aagtgtggcg
17641 tattacgagt cgtgctaagg ggaaatcaga aggtgactat atcaccttcg ctgacacgga
17701 tgttaatgct ttagaagaaa ttaacatgtt ccacccgact tacacctaca ttaacaagac
17761 tgtccgcgat tttgatgcgg tgccactttt ggtcccaatc tacgacaagg ggcaactaat
17821 ctatgatttg ccaagtcttg atgaaatcaa gaactatgcg actaagaaat tggatgagct
17881 ttggaatgag tacaagcgcg ttcttaaccc ccaagattat ccagttgact tggccaaaga
17941 tgtctgggat cacaagatga ccttgattga taatatgcgt aagaaagccc atgacttgtc
18001 agagtaaaag gagaaaatat gactttgcaa gaaactatta ttgctcaact aggagttaaa
18061 cctagcatta atccgaagga agagattcga aaatccgttg atttttgaa ggcctacatg
18121 atcaaacatc cattccttaa aacatatgtt ttagggattt caggaggtca ggattctaca
18181 ttggctggtc gtcttgcgca acttgcagtt gaagaacttc gtgcagagac agaaaaagac
18241 tatcagttta tcgctattcg tttaccatat ggagttcaag cagatgaaga cgatgcccaa
18301 cgtgccctat cttttatcaa gccagatgtg agtcttacga ttaacattaa agaagctgtt
18361 gatggtcagg ttgctgaact ggccaaggca ggtgtaaacg tatctgactt taataaggga
18421 aatatcaagg cccgccaacg tatgataacc caatatgcgg ttgctggtga aaatagtgga
18481 gcagttattg ggacagacca cgccgctgaa aaccttacag ggttcttcac aaaatttggt
18541 gatggtggtg cggatatcct gccactcttt cgtctaaaca agcgtcaagg cgctgccctt
18601 cttgcagagc ttggtgctga taaagccctt tatgaaaaag taccaacagc tgacctggaa
18661 gaagataaac caggaattgc cgacgaaatg gcattgggag tgacctatca tgagattgat
18721 gattaccttg aaggcaagga agtatcagcc aaagctcagg aaacgattga gacttggtgg
18781 cgtaaaggtc aacacaaacg tcacttgcca atcaccattt ttgatgattt ttggaaataa
18841 taagagaaat gatttatgat taaagcttat attgattttt ggaagcgtgc ctttgatttt
18901 agtggacgtt cgacacgccc agactactgg tgggtttact tgattaatgt catcattagt
18961 actatccttg tagtaggttt tgttctcatt cctttcttca gtaatgtgtc tcataaccca
19021 gcgatattaa atgactcaac tgaaatacaa aaaatgattt tgaaatatat gtcaccaata
19081 atgatttttg gattgatcat attaattcca caactgtccc ttcaatttcg tcgtttgcga
19141 gatgcaggta ttcatccggc ttgggggctt ctaagttgta tagggcttgt accaatcttg
19201 actttcttct cattcattgg aagtattgta ctcattgtca tgtcatgtca gccaactaaa
19261 ccagcacctg aaacgctttt tgataaagtt gaataagatt gtcgtacccc taggtttaac
19321 ctaggggtat ttttaaattg aattttcaga aattagtgat aaaatagata gtaataagtc
19381 ttaaaaggag gaatcttatg acttcactat caacagattt cacagaaaaa ctatttgcag
19441 attataaggc caatgccaag tatggcgcta ttgaaaatgc agtaacccat aacggcttgc
19501 ttaaatctat cgagacacgt caatcagaag tggaaaatga ttttgttttc tcaattgatt
19561 tgactaaaga tgaggtgtct aaccaaaaag cttcaggtcg ttgctggatg tttgcggccc
19621 ttaatacttt ccgccataaa ttgatttcag acttcaaatt ggaaagtttc gagctttctc
19681 aagcccacac atttttctgg gataaatatg aaaaatcaaa ctggttcttg gaacaaatta
19741 ttgctacagc agatcaagaa attggtagtc gtaaggttaa attcctttg gataccctc
19801 aacaagatgg tggacaatgg gatatggtcg tatcactctt tgaaaaatat ggtgttgtac
19861 caaaatcagt atatccagaa tctgtagctt caagcaatag tcgtgagctt aatcaatacc
19921 ttaataaact ccttcgtcaa gatgcccaaa tcttgcgcga tttgattgcc agtggtgctg
19981 atcaggctgc tgttcaagct aaaaaggagg agttccttca agaaatcttc aattatcttg
20041 ccatgaccct tggcttgcca ccacgtcagt ttgattttgc ctatcgagat aaagatgata
20101 actatcgatc tgagaagggt atcacgccac gggccttctt tgaaaaatat gttggcctta
20161 aactcagcga ctatgtatca gttatcaacg ctccaacagc tgataaacct tacggaaaat
20221 cttacacggt tgagatgttg ggaaatgttg taggtgcgcc aagtgttcgt tatattaacc
20281 tgccaatgga ccgattcaaa gagcttgcta ttgcacagat gaaggcagga gagagtgtat
20341 ggtttggttc agatgttggt caggtttcgg accgtcaaaa aggtattctt gcaactaatg
20401 tttacgactt tacagctagc atggatatta actggactca agacaaggct ggacgtttag
20461 actacagtga atctctcatg acccatgcca tggtattgac aggtgttgat ctggatgctg
20521 atggtaaacc aattaaatgg aagattgaaa actcttgggg agataaggtt ggacaaaagg
20581 ggtactttgt tgcttctgat gcttggatgg atgaatatac atatcaaatc gttgttcgca

20641 aagatttcct tacagctgaa gagttggctg cctatgaagc agatccacaa gtgcttgcgc
20701 cttgggaccc aatgggatct cttgcttcaa aataagagga tatttcaaaa acactggata
20761 gaattccagt gtttttagtt gacttcatta aaatttatgg ttgtaaaaag aggctctata
20821 atttctgtag tgggtaaatc ctctatggag attatggagc ctttttgatt gtagaaaaaa
20881 agtcccatat gacctataat gaaaagcgac taaaccatca ttttagaaag actcatatgg
20941 aacaactaaa tcttatcaca aattttcttg gaataaaaga caaaaatatt atcatcacta
21001 atgaatacga tatgggaatg cacaaagaac ttcacggtta cttggattac gagtctccta
21061 aatgtcctgc ctgcaaggga cagatgggca aatacgactt ccagaaagcc tctaaaatcc
21121 cgtatctgga gtgtgctggc taccgaactc tcattcgcct gaaaaagcgc cgtttcaagt
21181 gtaaggagtg cggaaaaatg gctgtcgcgg aaacttctct ggtcaagaag aatcaccaaa
21241 tcgcaaccgt cgtctatcag aaaatcgctc aattgctcat tgaaaaacag tctatgaccg
21301 acattgccaa gcgccttgcc gtctctacct caaccgtcag tcgcaagcta aatgagttcc
21361 aatttaaaac cgactggaat tggctgcctg agaatatgag ctgggatgag tacggtttca
21421 agaagggaaa aatgagtttc atcgctcagg atttcgactc attaaaagtc atcacaatcc
21481 tcgacggacg cacgcaagca acgattagaa accacttcct acgctattcc agacaggata
21541 gaaatcgcgt taaagtcatc actatggaca tgtttagccc ttattacgac atcgccagaa
21601 aattattccc taacgctaag attgtccttg atcgctttca cattgtgcaa caccttagtc
21661 gtgctatgaa ccgcgtccgc attcaaatca tgaaccattt ggaccgtcag tcacaggaat
21721 atcgtgccct gaaacgctac tggaaactga tccaacaaga tagtcgcaaa ctcaacgata
21781 aacgctttta tcgtccgact tttcgtgcgc atttaaccaa caaggaaatc ctagaaaagc
21841 tactgtcata ctctcaagaa ctgagagaac attatgaact ctaccaactt ttacttttcc
21901 atttccaaga gaagaactcg gaccatttct ttgacctcat tgaagagacg agttccagcg
21961 tacaccctat ctttcagaca gtttttagaa ctttttttgaa agataaagac aagattatca
22021 acgccctcga actgccgtat tcaaacgcaa aactggaagc aacgaacaat ctcatcaagg
22081 tcattaagcg aaatgccttt ggcttcagga actttgaaaa ctttaagaaa cggattttga
22141 tggcagtaaa tattaaaaaa gagaagacca aactggtcct ctcaagaatt tagcttttca
22201 tctacccact acagttgaca aagagcctaa aaagagctat ctaatggata actcttttca
22261 attccttttt actcatctac tgtagaagta gaagtgtttg aatttgaatt ctcagcaccg
22321 ttagaacccg atgttgaagg atttgttgat gcatctgggc catactgttg actatcttgt
22381 gaacttgatg agtcactact tttaccataa tccgaacttg atgagtcact acttttacca
22441 taatccgaac ttgatgatga gctagatgtt cctagtgatc cgttataaac atttgacgtt
22501 gacgaaccag ttaggaagac gtaactacca ttgttgtaaa caccactagg catatcgaaa
22561 tctttcgaac ctgcaccata catctggttg aggtatctac ttgttagacc gtagatttgt
22621 ttagcgatat ttaggtctga gccataaagt ggagttttac ggttttcata acctgtccaa
22681 atggccattg catatttagt attataacca acgaacgttt cgtcaggaac aataagatca
22741 gcgtagacgc cactttcttt ttgtacttga tagtactcat cgtcactgta gttagatgta
22801 cccgttttac cggcattgat cgtgccaggt acataagctt cagtcgctgt accacctgtc
22861 aaaacctgtt tcaacatgct attcatcata taagctgttt gtggcgacat ggcctcgaca
22921 cctttagaat cgtatgattt tgttgaacca tcttcaaatt taattgattt aatgtaggaa
22981 ggtttatagt aagtaccacc attggcaaag gctgcatagg cagccgccat cttctcactt
23041 gaagcaccat atttaggatc tgatgatgtt gttgaagatg agatagcgtt tgaatagaac
23101 atctctgggt actcaatacc taaatctttc aaaaaggatt gagcataatc caagccagtc
23161 gcttgaagcg ctttaaccgc agtaacgtta cgtgattgtt gaatcgcata agtcagagag
23221 atacttccgt aatacttgcg gtcccagttg taaactggag ttgaagtacc tgggaaatta
23281 taaggggtat cgtaaacggt agtaccagtg ttagtatata cccttttttc aatagcaggt
23341 gcgtaatcgg tgataggttt cattgtagac ccccagtcac gatcagtttg aacagcttgg
23401 tttgtaccaa gggcaatgtt ttcgtcttgg tgacgtgatc ccaattgagc aacaacacgt
23461 ccattggtaa catcaatgat agtagaagct acttgaaatt tgtcatcagg ataagcaata
23521 taatcatttg agttgtagac attccaaatg tatttttgaa tatcaggatc aagatttgta
23581 tagacatcga gtccagctga gtagatatca gcattggctt cggtagagac ttgttcaata
23641 acttgtttga tgtagttatc taggtatggt tcgtagctag ctttattcgt aagtggaaga
23701 agaccatctg atacatccgt tgctttagct tgctcgtact cctctttaga aatatttttg
23761 tcttcataca tttcattaag aacagtgttg cgacgttctt tagcagcatc cggattagca
23821 taaggattat attgtgttgg tgcttgtggg ataccagcaa gaagagctgc ttgggcaact
23881 gagaggtcac ctaattcttt accaaaataa gatttagctg cggtcttcat accgtagtaa
23941 ccattaccca tataaacctt attaacgtag aaggtcaaga tttcttgttt agtatactga
24001 cgctccattt ggagagataa ccatatttct tgtgacttac gtttaagggt ttggtctgag
24061 gtattagttg agaagtaggc taacttaatc aactgttgat caagtgtaga tccccccttga
24121 gtgctgttat ggcgaaggtt gttaatagcg gctcccataa tacgataaac gtcaatgccg
24181 cgatgggtaa agaaacgctt atcctctata gaggtaattg cattgactag gattatagga
24241 atctcatctg ttgatgcact ttcacgtttt tcggatccca agtcagcaat aagaccacca

24301 ttaccatcgt aaatcctact tgagtttgtt gattgcaact tgttctcggt aagtttagga
24361 acactgctaa catagtaagc aaagagtcca ccaccagcga tgacacagat tacaaagaaa
24421 gtgagtagtc caataaaagc gtattttgca atacgccatc ctaaatttg atctgaagca
24481 gctgagcgtt ttcctttgct atagcgagaa gaatctgaac gtttaacctg tgtgttttta
24541 gcacgtttag ttgaaccgtt aagtgattct ttaaagttgc tgaaattaaa tttagccatt
24601 atgtgttacc acctaataat ttttcaatga tctctaaata aggaacttga ggataagctg
24661 attctgcgat tgcataacct ctttttttga tataatcaag aggcattgat tttgtcccct
24721 tatcaatttg ataaaaagcg attaagtctt tggcaggcag aaggtaggtt tccttaagcg
24781 ttgaaaagtg gagtagcacg aagcagattc catcttgatt gatgacctga gacatgtgct
24841 taatttgatg cgcatgaaag tttttcatag gcatagatgt cttttgccgt gtctctttgg
24901 cttcaaaatc aatatagtat cctttgtaaa cgccagagta gtcagtagtt gaggcttgcc
24961 taaagtaagc ttctacaatc tttgctcgtg agcgttttgg ataatcgact ttgacgatct
25021 gaaccggtgt aggtttctta tgaataacgg ctaagccacg cgacaaatag taatcattag
25081 tcgcattaat agcagattca aagttcatcc cccgattggc aaagtcaact ctattagatt
25141 tttttagtac gtacttgtgc tatcttacga gatatctgat ggggatagtt taccatttct
25201 ttcccttctt atttataaga tatgcataaa gactatttca ttataccata aaatgagcga
25261 ggaaaacaat aaatgacatc actactcata acgggctata agagctttga acttggtgtt
25321 tttaaagaca aggatcctaa ggtaaacatc attaagaaag caatcaaacg tgatcttaaa
25381 cgttttttta gatgaaggtg tggattggat gatttttact gggaaccttg gttttgaatt
25441 ttgggcgcta gaagtggcta aggaacttca gaaagactac cctttgaggt tagcgacgtt
25501 atttccattt gaaacacacg gtcagaattg gagtgaagct aaccaagaaa aattggcagc
25561 ctttaaacaa gttgatttcg taaagtatag ttttccagct tatcaatcgc cagctcagtt
25621 caaacaattt aatcagtttc tcatcgataa tacagaccaa gcttatttat tttatgagcc
25681 tgaaaatgaa acaaatttga aatattttta caatatgata atagcagcaa gtgattatcc
25741 cttatttcga ttgacttttg atgacctcaa tgaagtcatg tcggaatagg atataattac
25801 gttttaaact tgcattttta tattttttc tgtatcataa aaacaataga ctgtaaagta
25861 ttagtaagtt agataagaca agtattggag aagaataatg gctaagatta atttaacgcc
25921 caagaaaatc tacgaacaag aatttaaaac aagcatacgt ggatacgata aaacagaagt
25981 tgatgagttt ctagatgatg tgatcaaaga ttacactgtc tacattgctc tagttaagga
26041 attgcaagaa gaaaatgcaa aactgaaggc taaggcaaca tcggctcctg caagccgtcc
26101 tgcatatgca agtgcgacaa gtgaaccatc acatgccact gccaatattg cttcggcttc
26161 aaactatgat atcttaaaac gtatcagtcg tttggaaaaa gaggtctttg gaaaacaaat
26221 cgtcgaataa aactggtttt gtgcaatttt cggataatcg catggttgca agtcttccat
26281 gaggaaagtc catgctagca ctggctgtga tgccagtagt gtttgtgcta ggcgaatcaa
26341 taagcctagg gacttgattt tcaagttacg gcgagcgaac tggctaagtc tgcggatagg
26401 ttttagtagc tctgaaagtg ccacagtgac ggagttttta gggaaaccta aaaagtggaa
26461 cgcggtaaac ccctcaagct agcaacccaa actttggtag gggcatggga tggccggaaa
26521 cgaacggacc attctgactg ctatgcagta gacagatgat tatcgaagga agtggtacct
26581 agtcacttct ggaacagaac atggcttata gaaaattgca tataggtgag gctgggacac
26641 atgtctcagc cttcttttag caagaatcgt ctcttttgcg tctaccatct aagggcaaga
26701 gtgaataata agctatagct tagggaaatt tgtactcgta ataattaggt aaaaggatga
26761 caatgaaaga aacatttaca ttagtagcaa ctgctgcagc cggacttgaa gctgtggttg
26821 gacgtgaact acgtgagttg gggtatgaga cccaggttga aaatggtaag gtgcgttttc
26881 agggtgatgt tcgtgcaatt gctgaaacta acctttggtt gagagctgca gaccgtatca
26941 agattgtggt gggtgagttt ccagcacgta cttttgagga gctttttcag ggagtttttg
27001 ctttggactg ggaaaattat cttcccttag gtgcaaagtt cccaatttca aaaactaaat
27061 gtgttaaatc aaagcttcat aatgagccgt ctgttcaggc gatttctaaa aaagctgtcg
27121 ttaaaaaact acagaagtat ttccaccgtc cggaaggagt gcctcttcaa gaaacaggtg
27181 cggaatttaa gattgaagtt tctatcttaa aggataaggc tactatttta attgatacta
27241 caggcgctag tctttttaag cgtggttatc gtactgataa aggtggggca cctattaaag
27301 aaaatatggc agctgctatt cttgaactta gcaactggta tcctgacaaa cctttgattg
27361 acccaacttg tggttcgggg accttctgta ttgaagctgc tatgattgga atgaatattg
27421 caccaggttt taatcgtgat tttgcttttg aggcatggaa ttgggtgggt aaagatttag
27481 tacagtcagt acgagatgaa gctgatagca aggcaaatta cgatgttgca ttagacatta
27541 tggggtgtga tattgatagc cgtatggttg agattgctaa ggctaatgct cgcgaagctg
27601 gtcttgaaga tgttgttaaa ttcaaacaaa tgcgtttaca ggacttgaaa acagataaaa
27661 taaacggtgt cattatttca aatcctcctt atggggagcg cttgttggat gacaaggccg
27721 tggatatctt gtataatgaa atgggtcaaa cttttgcgcc gcttaaaacg tggagcaagt
27781 ttatcttgac cagtgatgaa caatttgaaa gtaaatacgg catgaaagct gataaaaaac
27841 gtaagcttta taacggaaca cttcgcgttg atttgtatca atattttgga gagcgtgttc
27901 gccgttctga ggctagaaag gtaaattaaa cgtgtcagaa gataaaacac aaaatggcta

27961 tgaagaaagc caagagttag atttccaaga tgccaaggaa atgacagtcg gtgaggctgt
28021 ccgcaaagaa gctgagatta atgctggtgt aaccgaaaca gatagtattt tggataagta
28081 tattaagcaa catcgtgaag aagttacctc acaaaaattt tcaaagaaaa ttgaagcaga
28141 tggcgataca tcacccttgg atgccttcat tcaaaagcaa cgtcaggaat ttgctgattc
28201 aggtttgatt ggtcaaacat tggctaatga gtcaactaac tcaacgactg ctgacgaaac
28261 agtaacacct attacatctg gttttggcac aactgaaact aaggcagatg atactcaagc
28321 tcctgttgac tctgaaatca ctgtgaaacc agaaagtgaa agctctgaaa ccattataac
28381 atcaactaat gctgaccgtt ttatcacgtc agaagcagaa aaatttgatc ttggtgaagc
28441 ccttgctgcg acgactacct caactaatca acaagttgat aacactgcta tggtagacaa
28501 tgttgatgat ccggaaccag aaagtacttt acctgctgat gacaagtctt ctgaaccaca
28561 agcctctaaa cctctgttgg aagatgttgg agtctcagaa acaatcgact cagacgctat
28621 tgccacaaca gttgctggtg taacaggagt taaagctgat gaagcaaaag cgacaccaaa
28681 agtgtctatg acagttagtc gtccatcagc agaagataaa atctcttcag ggtctatttc
28741 tccatctcat aaaggtgtct ttgatggtga tattccagtt tatcgccgta agggtgttgt
28801 tatcggtgct ctcacggttc ttgctctagc tataattggt gggagctatg ctctttataa
28861 agtaacacat agccaatctg cgaaaactac aagcactgct tcttcagcag tgatatcatc
28921 atcaagcaag ggcgcatcag cttctgataa taaagccttc gaagacatgt ataagaattt
28981 cttcacagat gatgagcaaa caaaactagc taatgatcaa tttggtaagc tatcagactt
29041 ggaaaaacta cttaaaaaat tggaaaaaac caagtattat gatgcagcta agaccaaata
29101 cgataacttg aagaaacaaa tcgaagctat tgaaaaagtc aatagtaagt atgaatcaga
29161 tgctcttgta gacggtagct ataatgcctc tatctcagtt aaatctgatg caaactttaa
29221 tgatctttca gaacgtgtaa caaatactgg caatgctagt cttgatagca ttatccaaga
29281 agtcattaag ggcggtaaaa cacagcttga agaaaagggt aaggctgcaa gtgcaacgtc
29341 tgcagtgaca gcttctgaaa gtgttaatac cgctactcct tcgggagaca acacatctgg
29401 agcagctaat tcaggtgtaa caggtgcggc tggtggagtt tctagtggta cagctgcaac
29461 ttcaacagtt gtgacacgtg gcatcatgaa ttacaatcca tctatccttc aacgtgatcg
29521 ctctcgtgtg ccttataatg caaacgtagt agcggataca agtaatccag cttggacttg
29581 ggctgatggt gttcttgata agatcattgc aacatctcac tcacgtgggt acttctcagg
29641 agataacttt atccttgagc cagtcaacat catcaacgga aatggttatt acaacatgta
29701 ccttccagat ggaacttatc tctttagtat caactgtaag acaggttact atgtaggcaa
29761 tggttcaggt cactcagata aacttgatta ttaataaaaa tcgacgacac tccctaggga
29821 gtgttttttt tgttttatct ttgtgaagtc cgaatttata agagttttgt gctataatag
29881 ggggataaga taggggtgaa tactatggcg aaattgattc cgggtagagt ccgtaatgag
29941 ggaattaaac tttttgaaaa gggtttaatt gccattagtc aggtctcaga aactcagttg
30001 gacacgacgg ttggtcaaca tcatttaatt tatgcactcg atgatcctga aatcatgtgt
30061 gactgtgatt ttttcgctca aaaaggctat tgctcccatt tggcagcggt tgagtactat
30121 ctaaaaaaatg ctaaggaggg ccagcgtctc ttagcaaaat tagaggagaa acaagagtcc
30181 gcccaggacc aagagcgggg gcgttctttt ggtggtttat ttttagagag cctttctctg
30241 aatgagaatg atacagttag atatagcctc acggtagagg gagaggaatc tacctttggt
30301 tcagagattt ggtggagttt acgtttacgt cgcttaccgg acgaacgttc ttacgttgtt
30361 cgagatatcc cagccttcct caagcttata gagactgaag gctattatca gataggtaag
30421 aattactatg aacctctatc actcattcag tttgaccagg cttcacaagc atttttagac
30481 tttttaggac ggatgattcc agatgaagcc aaaacaaatt taacctttat tttaccgaat
30541 aatgcacgac accttagtct cccctatggt ttttttgagg agggattgag gttgatgcaa
30601 gatttggatg gcttcaggtt tgaatgggaa ggtattgaat atcgcagctt tttggtagag
30661 gacttgactg ctgaagccaa tcttttctcg tttgatattt gtgtcgagcc caaaatgatt
30721 gaattgactg ttgctgagaa aaatagccaa acatttttta ataatcgtat tatcttttat
30781 cagggtgttt tctatcgtct taatagaaaa cagcaaaaaa ttttgcttgg cttacgttct
30841 ctccctatcg gaagcgattt gaataaacat gtcagtttta atctagaaga acaagctatc
30901 ttagctgcta gtctttctga ttttaagact atgggaccgg ttaaagcccc aaaagcgttt
30961 aatatcaaag atttcacccc gagatttcgc tttgatttga aaggtgaacg cgaggttgtg
31021 ctcacattgg cttttgattt tgatggatat gttgtggata atcgttatga gttaagtcat
31081 ttaggcttta ctagtaacta tcgtaatgaa caggctattt ttcgtttgat ggttaaacat
31141 ggatttaccc ctgattttca gtcatctaaa cgcttaaata gtaatcaaga gctctatgat
31201 ttctttataa atactctacc agcttttgaa aatgcagggc cagttcttat tggacaggag
31261 ttgcgtgact tacgtgttga acagagtcca caaattcagg tagagcgtca aggaaatcta
31321 ttagagattt cgtttgattt ttcaagtctc gatgatgagg atgtggatca tgctttagaa
31381 gctttgatgg acagagcgcc ctactttgtt aatcgtagtg gtcagttgat tgtctttgat
31441 gaggggacac aacgtatcag tgagagtcta agaactttga gggcccgtta ttcgggggaa
31501 gggcatctag aactccatca attagccgcc tatcaattga tggactctct ttcagaaaat
31561 gattctgttc gtttttcaga agattttcaa cagctagcgc aacatttgag aaaaccggaa

31621 acattcgact tgccatccta tcatgtagaa gttgaccttc gtccttacca agaaagagga
31681 gttcaatggc tatcaatgtt gcatcactat ggttttggtg gtattttagc cgacgatatg
31741 gggttgggga aaactttaca gaccattgct tatctttctg cacatttaga ggatggacaa
31801 cgagtcttga ttctatcgcc atcgagttta atttacaatt ggcaggatga gtttaaaaaa
31861 tttgctcctc agttagatgt agcagtttct tatggactta agccaaaacg tgatgagatc
31921 attgcagaag accatcagat tattattact agctatgctt cattccgtca ggattttgat
31981 gtttataagg ctggtcagta tgattatctc attctagatg aggcacaggt catgaagaat
32041 actcagacga agatttctca atatttacgt gatttttctg ttccgcattg cctagctcta
32101 tctggaacgc ctattgaaaa tcatttgttg gagatttggt ctattttcca gattgtttta
32161 ccagggcttt taccagctaa gaagactttt cttaagctaa cacctatgga agtggcccgg
32221 tacattactc catttattct tagacgtaaa aaagatgagg ttcttcctga tttaccggac
32281 ctgattgaaa ttacacacca aagtgaattg tcggatagtc aaaaggctat ttatttagca
32341 caattgcaac agatgcaaca aggtttaatt tcagcaagtg atcaggaaat caatcgtcgt
32401 aaggtggaaa tcttgtcagg aattacccgc ttacgccaaa tctgtgacac gccagccctc
32461 tttatggatt acgctggtga tagtgggaaa ttagatagtc ttagagacct acttagtcaa
32521 attaaggaat cagaccatcg tgtccttatt ttttcacaat ttcgtgggat gttagatatt
32581 acagaggaac ttttacaaga gttaggtatt tcctcttata agctaacagg gtcgacccca
32641 tctggtagtc gtcaggagat gaccagagcc ttcaatcagg ggagtcgaga tgcctttttg
32701 atttctttaa aagcaggggg ggtagggctc aacttgactg gtgctgatac agttattctg
32761 attgaccttt ggtggaatcc agcggtggaa atgcaggcta ttagtcgtgc tcatcgtata
32821 ggtcagaaac aaaatgtcga agttttccgt ttgattacaa gaggtactat cgaagagaag
32881 attcttgagc ttcaagaagg taagaaaaat ctcgttacaa cagtattaga tggcaatgaa
32941 agctgtgcta gcatgacagt tgaggatatt aaagaaattc ttggtattgc aatatcttaa
33001 tctgttatac ttaatattgg aatactaaga tataaaaatg acactaggca aatcttttga
33061 attaggatat aatagaatat tgcaagacgt atctccccat tcccgattgt ttatattaag
33121 atacgtatca tctcgaaaac gaaaggaagt cttttatgca tcgccaaaga cgacaatttc
33181 cgttgattcc ggacggagaa tcttgcttgc aagaacctat ctctatgcgt ttatacgaaa
33241 atgaagattt aatcactaat atccgtggtc cttatcagga taaggactat aacgatttct
33301 ttctgaacca tgatttcttg tcagctaagc ctcataaacg taagcaggct ccagaccgag
33361 ctgaaacaca agaggatgga atgacttatg ctgaagaagc ccgtgaaaag gcgaagcgtg
33421 acgtacgtga taaacgccaa aaatacctac gccaagaagc tcaatcaact aaatctacga
33481 atatccgttc actagctgct aagcctaagg tagaaaataa gctaagcttt gaggctactg
33541 ttgaggcagt tgctgtaacg acagataagg agccagtgat gacgtctatc ttgggagctc
33601 ctgtaagtgc tattaagaga acattagcac caaatggtaa acactcaaaa atccatcact
33661 tggcaaatcg tctgaaacaa gatacttata tcttggcaga agtggctcca acttatcaac
33721 aacctagtaa tccatctcgt aagaacgtca agaaaaatag ctatgacttc ttgaaacgta
33781 gccaggttta taattacaca gaacgtcaaa ttcaccgtga acaccgaatt gctcaagaac
33841 taaatttgac gcatttagaa gatgcaaatt aatcgcagtt tgattgcaag gagaaactat
33901 gtcaaaaact tatcatttta ttggaattaa aggatctggt atgtcggctt tggctttgat
33961 gctccatcaa atgggacaca aggttcaagg ttccgatgtg gaaaaatact acttcactca
34021 acgtggtctt gagcaagcgg gcattccaat ccttcctttc gatgaaaaaa acatcactga
34081 agatgttgaa ttgattgctg ggaatgcttt tcgtgaagat aacaacgttg aaattgctta
34141 tgccattaaa aatggttata agttcaagcg ttaccatgaa tttttgggtc atttcatgaa
34201 tcagttcact agctttggtg tggctggtgc acatggtaag acctctacga caggactctt
34261 gtcacactct atgaaaaaca tcacagatac gtcatacctt atcggtgatg gaacaggacg
34321 tggttctgcc aatgcgcaat actttgtctt tgaagcagat gaatatgaac gtcatttcat
34381 gccgtaccat cctgcttact caattatcac taacattgac tttgaccatc cagattactt
34441 tacaagtatt gatgatgttt tctcagcatt cgatgattat gcaaagcaag tacaaaaagg
34501 gctctttgtt tacggtgagg attctaacct ccgtaaaatt acatccaatg caccaatcta
34561 ttattatgga tttgaagaaa atgatgactt catggcaaca gatattattc gtacaaccac
34621 ggggtctaac ttcaaggtga agcatgatgg acaaatcata ggggaattcc atgtaccagc
34681 ttatggtcgt cacaatatct tgaatgcaac tgcggttatt gctaacttgt atgtagctgg
34741 ttttgacatg gccttagttg ctgaacatct aaaaactttc tcaggggtta aacgtcgttt
34801 cacagagaaa attattagtg atactgttat catcgatgac tttgctcacc acccaactga
34861 aattattgca acactagatg cagcacgtca aaagtaccca tctaaagaga ttgttgctat
34921 tttccaacca catactttta cccgtaccat tgctcttctt gatgactttg ctgaggcttt
34981 aaatgaagcg gatgctgtct atctggcaca aatttatggt tctgcgcgtg aagttgacca
35041 cggtgatgta aaggttgaag atcttgccgc taagatcaat aaacctgcca aagtggttac
35101 agttgagaat gtttcacctc tccttgacca tgacaatgct gtctatgtct ttatgggagc
35161 tggggacatc caactttatg aacgttcatt tgaagagctc ctagcaagct tgcaaaccca
35221 catgtaatat attaagggct gtagcgattt cgctcggctc tttctcttgg atagaaagga

35281 atcttagatg attattcgac acgctagaac gaatgattgg actgatgttg ttcgtatcga
35341 acaagataaa tttttcgcct gaagaggctg ctacaccaga ggctcttgca gagcgtttag
35401 agacaatttg cgatactttc ctaattgctg aaattgatgg cactgtagct gattatatcg
35461 aaggacctgt gatttcggaa cgatatctta cagatgactt attctacaag gtaactccta
35521 atgctagtca aggaggctat atcgcggtta ctagtttatc tatctccaaa gactttaaaa
35581 ggcaaggtgt tggaacatcc ttgattgcag tttttaaaga cctggctatt gctcaaaaac
35641 gtcaagggat tagcttgact tgtcatgatt acctgattac ctactatgag atgaatggtt
35701 tcattgacga aggtgagtcc aaatcaaacc atggcgatag tatttggtat aatatggtct
35761 gggagaaccc agaaggctag taataccagt gttacgttac tgtgacaaag aaccaattga
35821 tttgcattaa ctggtttttt aagatataat ggcgtttgtt actatagagg aggtatgcct
35881 tgacggacaa gcataatgaa tactcggaat ctgctgaaga tagcctaagc tttaaaaatc
35941 agattcttcg agatttacaa gaagcgacac ggctccgttc tcttcgtgag gaggagcata
36001 aaaaaagtgc agctgttccc gagacaccac tttcaatgtc agctgatagc catgcaatag
36061 attctggatc agcgtcaaat aaagtaagta accaaaattt gagttctcat tctgttgctc
36121 atagtgctat agctaaaact atgacttctg agacggttcg agattttcaa tatagtgttg
36181 acttgtcttc taattctcaa gctgattctt cagcccctga tgtccattca aacgtaatat
36241 caagtcctaa agagcaagtt gataaggaga aaaatttaag tattcctcag gagactgaaa
36301 tattgaaacg tcgttttaaa cgtccagttt gggaaactag tcttaacgcg gaatcagagg
36361 aagaatatat tcctgcagat gttgctaagg agcttattga agctaaggct aaagaagcta
36421 tctacacgaa tcctaaagaa ttggtgtcca agatgacatc agaacgtcaa aaagaagcat
36481 ttttgcggga acatcataca gtaagtaatc actctgaacc agtaagagtt cagtcagatc
36541 tagtaactga tggcaatgag ggtgatgaga gtattgcttt agcagcagcc tctgaaaaat
36601 ctaacaaaaa gaaacgtaag aaagtgaaga agaaaaagtc ttcaccaaaa gatgttaaac
36661 aggacggaat catcaacgaa gaaccgattt catgctccaa ccgaaatcaa aaactcaata
36721 aaaataatcg tcgtgcagga cgagttgcaa gaaacatcat tgtcttcttg attcttatct
36781 tatcattagc tggattcttt ggttatcgat atgttagtga tgcagttggt gcaaaagatg
36841 tcaagtcgac taagtttatt agtgttgaaa ttcctgaaaa ttcaggtagc agttacatcg
36901 gtcagttgtt ggaatcagct ggtgtcatta aatctggtaa agtctttaat tactatacca
36961 agtttaagaa catttcaaat ctaaaaagtg gttactataa ccttcagcct agtatgacta
37021 tggatgaaat tattgaggca cttcagaaaa aaggaagtga taagcctcag gaaccatctc
37081 tgggtacagt cttggttaaa gaaggataca ccattgagaa aatcgccaag gcggttgaag
37141 ttaattcatc agctaaaaaa ggtaaacatt catctactgg acttaaggaa aaagacttct
37201 tgaaacttat gaaggatgat gctttcatta ctaagatgaa agcgaagtac ccaaccttgt
37261 tggcaaacct tccgaattct acagatgcta agtatgtcct agaggggtat ctgtttccag
37321 cgacttataa tattcatgac gatacgacgg tggaatcttt ggtagaggaa atgctatcta
37381 cgatggatac ccacctatca ccatactatg caacaatttt atcaagtaac cacaacgtta
37441 atgaaatctt gactctagct tctcttgttg aaaaagaggg agcaactgat gatgaccgta
37501 aaaacattgc tagcgttttc tataatcgtc ttaattcaga catggccctg caaagtaata
37561 tcgcggttct ttatgttctt ggaaaacttg gtcaagaaac gaccttgaaa gaagatacaa
37621 cgattgatac gaatattgac tccccttata atgattatgt tcataaaggg ttgatgcctg
37681 gtcctgtaga tagtcctagt ctgtcggcaa tagaagcggt tatcaatcca tcttctacca
37741 agtacatgta ctttgtggcg gatgtatcaa ctggtaatgt ctattttgct gagagctatg
37801 aagagcacca gcataatgtt gaaacttata ttaatagcaa actaaaagat aaatagtctt
37861 attggttaat taaaattcag tcactgacta ctgagtttga tttcaaacca actggctaaa
37921 aaataggaaa gagaaaaaaa aatggcagaa aaaacatatg taatgaccct agcagaaaaa
37981 aaaacaactt gaagcagaat tggaggagta caaactcgta cgtcgcccag aagttgtaga
38041 acgcatcaag attgcccgtt cttatggtga cctttcagaa aactctgagt atgaggcggc
38101 taaagatgag caagccttta ttgaaggtca aattcaaatt cttgaaacaa agattcgtta
38161 cgcagagatc gttgatagtg atgctgttgc caatgacgaa gttgcgattg gtaaaacagt
38221 tgttgtgcaa gaagttggta caagcgacaa agatacttac catatcgttg gtgcagccgg
38281 tgctgatatt ttctcaggta aaatttcaaa tgaaagccca agtgggatcc ccgggtaccg
38341 agctcgaatt cgccctatag tgagtcgtat tacaattcac tggccg

//

Amp6

```
LOCUS       6A
DEFINITION
FEATURES             Location/Qualifiers
     gene            87..656
                     /rbs_motif="GGA/GAG/AGG"
                     /uscore="-6.43"
                     /rscore="0.91"
                     /sscore="-2.45"
                     /locus_tag="6A_01"
                     /conf="100.0"
                     /ID="6A_01"
                     /cscore="84.82"
                     /partial=0
                     /tscore="3.07"
                     /gc_cont="0.36"
                     /rbs_spacer="5-10bp"
                     /start_type="ATG"
                     /score=82.4
                     /codon_start=1
     CDS             87..656
                     /rbs_motif="GGA/GAG/AGG"
                     /uscore="-6.43"
                     /rscore="0.91"
                     /sscore="-2.45"
                     /locus_tag="6A_01"
                     /conf="100.0"
                     /ID="6A_01"
                     /cscore="84.82"
                     /partial=0
                     /tscore="3.07"
                     /gc_cont="0.36"
                     /rbs_spacer="5-10bp"
                     /product="cell division protein SepF"
                     /start_type="ATG"
                     /score=82.4
                     /codon_start=1
     gene            660..917
                     /rbs_motif="GGA/GAG/AGG"
                     /uscore="0.91"
                     /rscore="0.91"
                     /sscore="6.13"
                     /locus_tag="6A_02"
                     /conf="99.32"
                     /ID="6A_02"
                     /cscore="15.55"
                     /partial=0
                     /tscore="3.07"
                     /gc_cont="0.333"
                     /rbs_spacer="5-10bp"
                     /start_type="ATG"
                     /score=21.7
                     /codon_start=1
     CDS             660..917
                     /rbs_motif="GGA/GAG/AGG"
                     /uscore="0.91"
                     /rscore="0.91"
                     /sscore="6.13"
                     /locus_tag="6A_02"
                     /conf="99.32"
                     /ID="6A_02"
```

```
                     /cscore="15.55"
                     /partial=0
                     /tscore="3.07"
                     /gc_cont="0.333"
                     /rbs_spacer="5-10bp"
                     /product="membrane protein"
                     /start_type="ATG"
                     /score=21.7
                     /codon_start=1
     gene            917..1717
                     /rbs_motif="None"
                     /uscore="-2.97"
                     /rscore="-3.27"
                     /sscore="4.35"
                     /locus_tag="6A_03"
                     /conf="100.0"
                     /ID="6A_03"
                     /cscore="86.53"
                     /partial=0
                     /tscore="3.07"
                     /gc_cont="0.331"
                     /rbs_spacer="None"
                     /start_type="ATG"
                     /score=90.9
                     /codon_start=1
     CDS             917..1717
                     /rbs_motif="None"
                     /uscore="-2.97"
                     /rscore="-3.27"
                     /sscore="4.35"
                     /locus_tag="6A_03"
                     /conf="100.0"
                     /ID="6A_03"
                     /cscore="86.53"
                     /partial=0
                     /tscore="3.07"
                     /gc_cont="0.331"
                     /rbs_spacer="None"
                     /product="cell division protein"
                     /start_type="ATG"
                     /score=90.9
                     /codon_start=1
     gene            1961..2836
                     /rbs_motif="GGAGG"
                     /uscore="4.99"
                     /rscore="14.48"
                     /sscore="21.88"
                     /locus_tag="6A_04"
                     /conf="99.99"
                     /ID="6A_04"
                     /cscore="188.21"
                     /partial=0
                     /tscore="3.07"
                     /gc_cont="0.361"
                     /rbs_spacer="5-10bp"
                     /start_type="ATG"
                     /score=210.1
                     /codon_start=1
     CDS             1961..2836
                     /rbs_motif="GGAGG"
                     /uscore="4.99"
```

```
                     /rscore="14.48"
                     /sscore="21.88"
                     /locus_tag="6A_04"
                     /conf="99.99"
                     /ID="6A_04"
                     /cscore="188.21"
                     /partial=0
                     /tscore="3.07"
                     /gc_cont="0.361"
                     /rbs_spacer="5-10bp"
                     /product="cell division protein DivIVA"
                     /start_type="ATG"
                     /score=210.1
                     /codon_start=1
     gene            3085..5886
                     /rbs_motif="AGGAG"
                     /uscore="4.11"
                     /rscore="11.88"
                     /sscore="18.4"
                     /locus_tag="6A_05"
                     /conf="99.99"
                     /ID="6A_05"
                     /cscore="588.14"
                     /partial=0
                     /tscore="3.07"
                     /gc_cont="0.388"
                     /rbs_spacer="5-10bp"
                     /start_type="ATG"
                     /score=606.5
                     /codon_start=1
     CDS             3085..5886
                     /rbs_motif="AGGAG"
                     /uscore="4.11"
                     /rscore="11.88"
                     /sscore="18.4"
                     /locus_tag="6A_05"
                     /conf="99.99"
                     /ID="6A_05"
                     /cscore="588.14"
                     /partial=0
                     /tscore="3.07"
                     /gc_cont="0.388"
                     /rbs_spacer="5-10bp"
                     /product="isoleucyl-tRNA synthetase"
                     /start_type="ATG"
                     /score=606.5
                     /codon_start=1
     gene            complement(6642..6863)
                     /rbs_motif="AGGA"
                     /uscore="2.85"
                     /rscore="6.93"
                     /sscore="12.77"
                     /locus_tag="6A_06"
                     /conf="89.11"
                     /ID="6A_06"
                     /cscore="-3.63"
                     /partial=0
                     /tscore="2.69"
                     /gc_cont="0.356"
                     /rbs_spacer="5-10bp"
                     /start_type="ATG"
```

```
                     /score=9.1
                     /codon_start=1
     CDS             complement(6642..6863)
                     /rbs_motif="AGGA"
                     /uscore="2.85"
                     /rscore="6.93"
                     /sscore="12.77"
                     /locus_tag="6A_06"
                     /conf="89.11"
                     /ID="6A_06"
                     /cscore="-3.63"
                     /partial=0
                     /tscore="2.69"
                     /gc_cont="0.356"
                     /rbs_spacer="5-10bp"
                     /product="transposase, partial"
                     /start_type="ATG"
                     /score=9.1
                     /codon_start=1
     gene            complement(6907..7605)
                     /rbs_motif="None"
                     /uscore="1.79"
                     /rscore="-3.27"
                     /sscore="1.59"
                     /locus_tag="6A_07"
                     /conf="100.0"
                     /ID="6A_07"
                     /cscore="61.66"
                     /partial=0
                     /tscore="3.07"
                     /gc_cont="0.416"
                     /rbs_spacer="None"
                     /start_type="ATG"
                     /score=63.3
                     /codon_start=1
     CDS             complement(6907..7605)
                     /rbs_motif="None"
                     /uscore="1.79"
                     /rscore="-3.27"
                     /sscore="1.59"
                     /locus_tag="6A_07"
                     /conf="100.0"
                     /ID="6A_07"
                     /cscore="61.66"
                     /partial=0
                     /tscore="3.07"
                     /gc_cont="0.416"
                     /rbs_spacer="None"
                     /product="transposase"
                     /start_type="ATG"
                     /score=63.3
                     /codon_start=1
     gene            complement(7736..9064)
                     /rbs_motif="GGAG/GAGG"
                     /uscore="4.62"
                     /rscore="6.9"
                     /sscore="9.17"
                     /locus_tag="6A_08"
                     /conf="100.0"
                     /ID="6A_08"
                     /cscore="160.69"
```

```
                     /partial=0
                     /tscore="-3.65"
                     /gc_cont="0.414"
                     /rbs_spacer="5-10bp"
                     /start_type="TTG"
                     /score=169.9
                     /codon_start=1
     CDS             complement(7736..9064)
                     /rbs_motif="GGAG/GAGG"
                     /uscore="4.62"
                     /rscore="6.9"
                     /sscore="9.17"
                     /locus_tag="6A_08"
                     /conf="100.0"
                     /ID="6A_08"
                     /cscore="160.69"
                     /partial=0
                     /tscore="-3.65"
                     /gc_cont="0.414"
                     /rbs_spacer="5-10bp"
                     /product="transposase"
                     /start_type="TTG"
                     /score=169.9
                     /codon_start=1
     gene            complement(9061..9960)
                     /rbs_motif="None"
                     /uscore="-2.76"
                     /rscore="-3.27"
                     /sscore="-9.5"
                     /locus_tag="6A_09"
                     /conf="100.0"
                     /ID="6A_09"
                     /cscore="65.23"
                     /partial=0
                     /tscore="-3.48"
                     /gc_cont="0.394"
                     /rbs_spacer="None"
                     /start_type="GTG"
                     /score=55.7
                     /codon_start=1
     CDS             complement(9061..9960)
                     /rbs_motif="None"
                     /uscore="-2.76"
                     /rscore="-3.27"
                     /sscore="-9.5"
                     /locus_tag="6A_09"
                     /conf="100.0"
                     /ID="6A_09"
                     /cscore="65.23"
                     /partial=0
                     /tscore="-3.48"
                     /gc_cont="0.394"
                     /rbs_spacer="None"
                     /product="transposase"
                     /start_type="GTG"
                     /score=55.7
                     /codon_start=1
     gene            complement(10097..10345)
                     /rbs_motif="AGGA"
                     /uscore="3.63"
                     /rscore="7.79"
```

```
                     /sscore="14.44"
                     /locus_tag="6A_10"
                     /conf="99.9"
                     /ID="6A_10"
                     /cscore="15.63"
                     /partial=0
                     /tscore="3.02"
                     /gc_cont="0.386"
                     /rbs_spacer="5-10bp"
                     /start_type="ATG"
                     /score=30.1
                     /codon_start=1
     CDS             complement(10097..10345)
                     /rbs_motif="AGGA"
                     /uscore="3.63"
                     /rscore="7.79"
                     /sscore="14.44"
                     /locus_tag="6A_10"
                     /conf="99.9"
                     /ID="6A_10"
                     /cscore="15.63"
                     /partial=0
                     /tscore="3.02"
                     /gc_cont="0.386"
                     /rbs_spacer="5-10bp"
                     /product="Putative transposase, ISSmu2, partial"
                     /start_type="ATG"
                     /score=30.1
                     /codon_start=1
     gene            complement(10457..10669)
                     /rbs_motif="GGA/GAG/AGG"
                     /uscore="3.38"
                     /rscore="-1.74"
                     /sscore="4.22"
                     /locus_tag="6A_11"
                     /conf="99.91"
                     /ID="6A_11"
                     /cscore="26.42"
                     /partial=0
                     /tscore="2.58"
                     /gc_cont="0.362"
                     /rbs_spacer="11-12bp"
                     /start_type="ATG"
                     /score=30.6
                     /codon_start=1
     CDS             complement(10457..10669)
                     /rbs_motif="GGA/GAG/AGG"
                     /uscore="3.38"
                     /rscore="-1.74"
                     /sscore="4.22"
                     /locus_tag="6A_11"
                     /conf="99.91"
                     /ID="6A_11"
                     /cscore="26.42"
                     /partial=0
                     /tscore="2.58"
                     /gc_cont="0.362"
                     /rbs_spacer="11-12bp"
                     /product="transposase"
                     /start_type="ATG"
                     /score=30.6
```

```
                /codon_start=1
gene            complement(10733..10939)
                /rbs_motif="None"
                /uscore="-6.49"
                /rscore="-4.0"
                /sscore="-6.86"
                /locus_tag="6A_12"
                /conf="95.28"
                /ID="6A_12"
                /cscore="19.93"
                /partial=0
                /tscore="2.5"
                /gc_cont="0.357"
                /rbs_spacer="None"
                /start_type="ATG"
                /score=13.1
                /codon_start=1
CDS             complement(10733..10939)
                /rbs_motif="None"
                /uscore="-6.49"
                /rscore="-4.0"
                /sscore="-6.86"
                /locus_tag="6A_12"
                /conf="95.28"
                /ID="6A_12"
                /cscore="19.93"
                /partial=0
                /tscore="2.5"
                /gc_cont="0.357"
                /rbs_spacer="None"
                /product="transposase"
                /start_type="ATG"
                /score=13.1
                /codon_start=1
gene            complement(10953..11288)
                /rbs_motif="None"
                /uscore="0.62"
                /rscore="-3.27"
                /sscore="-5.6"
                /locus_tag="6A_13"
                /conf="99.68"
                /ID="6A_13"
                /cscore="30.54"
                /partial=0
                /tscore="-3.65"
                /gc_cont="0.357"
                /rbs_spacer="None"
                /start_type="TTG"
                /score=24.9
                /codon_start=1
CDS             complement(10953..11288)
                /rbs_motif="None"
                /uscore="0.62"
                /rscore="-3.27"
                /sscore="-5.6"
                /locus_tag="6A_13"
                /conf="99.68"
                /ID="6A_13"
                /cscore="30.54"
                /partial=0
                /tscore="-3.65"
```

```
                     /gc_cont="0.357"
                     /rbs_spacer="None"
                     /product="transposase"
                     /start_type="TTG"
                     /score=24.9
                     /codon_start=1
     gene            complement(11342..11452)
                     /rbs_motif="GGAG/GAGG"
                     /uscore="-0.67"
                     /rscore="2.98"
                     /sscore="3.63"
                     /locus_tag="6A_14"
                     /conf="92.25"
                     /ID="6A_14"
                     /cscore="7.14"
                     /partial=0
                     /tscore="1.32"
                     /gc_cont="0.387"
                     /rbs_spacer="5-10bp"
                     /start_type="ATG"
                     /score=10.8
                     /codon_start=1
     CDS             complement(11342..11452)
                     /rbs_motif="GGAG/GAGG"
                     /uscore="-0.67"
                     /rscore="2.98"
                     /sscore="3.63"
                     /locus_tag="6A_14"
                     /conf="92.25"
                     /ID="6A_14"
                     /cscore="7.14"
                     /partial=0
                     /tscore="1.32"
                     /gc_cont="0.387"
                     /rbs_spacer="5-10bp"
                     /product="transposase"
                     /start_type="ATG"
                     /score=10.8
                     /codon_start=1
     gene            complement(11534..11800)
                     /rbs_motif="None"
                     /uscore="4.06"
                     /rscore="-3.27"
                     /sscore="3.21"
                     /locus_tag="6A_15"
                     /conf="98.86"
                     /ID="6A_15"
                     /cscore="16.21"
                     /partial=0
                     /tscore="3.07"
                     /gc_cont="0.307"
                     /rbs_spacer="None"
                     /start_type="ATG"
                     /score=19.4
                     /codon_start=1
     CDS             complement(11534..11800)
                     /rbs_motif="None"
                     /uscore="4.06"
                     /rscore="-3.27"
                     /sscore="3.21"
                     /locus_tag="6A_15"
```

```
                     /conf="98.86"
                     /ID="6A_15"
                     /cscore="16.21"
                     /partial=0
                     /tscore="3.07"
                     /gc_cont="0.307"
                     /rbs_spacer="None"
                     /product="transposase"
                     /start_type="ATG"
                     /score=19.4
                     /codon_start=1
     gene            complement(12004..14640)
                     /rbs_motif="None"
                     /uscore="0.85"
                     /rscore="-3.27"
                     /sscore="-5.89"
                     /locus_tag="6A_16"
                     /conf="100.0"
                     /ID="6A_16"
                     /cscore="151.62"
                     /partial=0
                     /tscore="-3.48"
                     /gc_cont="0.37"
                     /rbs_spacer="None"
                     /start_type="GTG"
                     /score=145.7
                     /codon_start=1
     CDS             complement(12004..14640)
                     /rbs_motif="None"
                     /uscore="0.85"
                     /rscore="-3.27"
                     /sscore="-5.89"
                     /locus_tag="6A_16"
                     /conf="100.0"
                     /ID="6A_16"
                     /cscore="151.62"
                     /partial=0
                     /tscore="-3.48"
                     /gc_cont="0.37"
                     /rbs_spacer="None"
                     /product="Calcium-transporting ATPase"
                     /start_type="GTG"
                     /score=145.7
                     /codon_start=1
     gene            complement(14817..16535)
                     /rbs_motif="None"
                     /uscore="-0.44"
                     /rscore="-3.27"
                     /sscore="-1.29"
                     /locus_tag="6A_17"
                     /conf="99.99"
                     /ID="6A_17"
                     /cscore="223.05"
                     /partial=0
                     /tscore="3.07"
                     /gc_cont="0.376"
                     /rbs_spacer="None"
                     /start_type="ATG"
                     /score=221.8
                     /codon_start=1
     CDS             complement(14817..16535)
```

```
                     /rbs_motif="None"
                     /uscore="-0.44"
                     /rscore="-3.27"
                     /sscore="-1.29"
                     /locus_tag="6A_17"
                     /conf="99.99"
                     /ID="6A_17"
                     /cscore="223.05"
                     /partial=0
                     /tscore="3.07"
                     /gc_cont="0.376"
                     /rbs_spacer="None"
                     /product="anthranilate synthase"
                     /start_type="ATG"
                     /score=221.8
                     /codon_start=1
     gene            16780..17019
                     /rbs_motif="GGA/GAG/AGG"
                     /uscore="-4.86"
                     /rscore="0.87"
                     /sscore="-1.09"
                     /locus_tag="6A_18"
                     /conf="71.95"
                     /ID="6A_18"
                     /cscore="5.18"
                     /partial=0
                     /tscore="2.91"
                     /gc_cont="0.35"
                     /rbs_spacer="5-10bp"
                     /start_type="ATG"
                     /score=4.1
                     /codon_start=1
     CDS             16780..17019
                     /rbs_motif="GGA/GAG/AGG"
                     /uscore="-4.86"
                     /rscore="0.87"
                     /sscore="-1.09"
                     /locus_tag="6A_18"
                     /conf="71.95"
                     /ID="6A_18"
                     /cscore="5.18"
                     /partial=0
                     /tscore="2.91"
                     /gc_cont="0.35"
                     /rbs_spacer="5-10bp"
                     /product="phage protein"
                     /start_type="ATG"
                     /score=4.1
                     /codon_start=1
     gene            17348..19597
                     /rbs_motif="GGAGG"
                     /uscore="2.44"
                     /rscore="14.48"
                     /sscore="19.33"
                     /locus_tag="6A_19"
                     /conf="99.99"
                     /ID="6A_19"
                     /cscore="462.93"
                     /partial=0
                     /tscore="3.07"
                     /gc_cont="0.409"
```

```
                     /rbs_spacer="5-10bp"
                     /start_type="ATG"
                     /score=482.3
                     /codon_start=1
     CDS             17348..19597
                     /rbs_motif="GGAGG"
                     /uscore="2.44"
                     /rscore="14.48"
                     /sscore="19.33"
                     /locus_tag="6A_19"
                     /conf="99.99"
                     /ID="6A_19"
                     /cscore="462.93"
                     /partial=0
                     /tscore="3.07"
                     /gc_cont="0.409"
                     /rbs_spacer="5-10bp"
                     /product="5-methyltetrahydropteroyltriglutamate--
homocysteine
                     methyltransferase"
                     /start_type="ATG"
                     /score=482.3
                     /codon_start=1
     gene            19741..20661
                     /rbs_motif="None"
                     /uscore="-0.0"
                     /rscore="-3.27"
                     /sscore="-0.21"
                     /locus_tag="6A_20"
                     /conf="100.0"
                     /ID="6A_20"
                     /cscore="132.52"
                     /partial=0
                     /tscore="3.07"
                     /gc_cont="0.396"
                     /rbs_spacer="None"
                     /start_type="ATG"
                     /score=132.3
                     /codon_start=1
     CDS             19741..20661
                     /rbs_motif="None"
                     /uscore="-0.0"
                     /rscore="-3.27"
                     /sscore="-0.21"
                     /locus_tag="6A_20"
                     /conf="100.0"
                     /ID="6A_20"
                     /cscore="132.52"
                     /partial=0
                     /tscore="3.07"
                     /gc_cont="0.396"
                     /rbs_spacer="None"
                     /product="5,10-methylenetetrahydrofolate reductase"
                     /start_type="ATG"
                     /score=132.3
                     /codon_start=1
     gene            complement(20838..21644)
                     /rbs_motif="None"
                     /uscore="0.0"
                     /rscore="0.0"
                     /sscore="3.22"
```

```
                     /locus_tag="6A_21"
                     /conf="100.0"
                     /ID="6A_21"
                     /cscore="160.85"
                     /partial=1
                     /tscore="3.22"
                     /gc_cont="0.399"
                     /rbs_spacer="None"
                     /start_type="Edge"
                     /score=164.1
                     /codon_start=1
     CDS             complement(20838..21644)
                     /rbs_motif="None"
                     /uscore="0.0"
                     /rscore="0.0"
                     /sscore="3.22"
                     /locus_tag="6A_21"
                     /conf="100.0"
                     /ID="6A_21"
                     /cscore="160.85"
                     /partial=1
                     /tscore="3.22"
                     /gc_cont="0.399"
                     /rbs_spacer="None"
                     /product="phosphoglucomutase"
                     /start_type="Edge"
                     /score=164.1
                     /codon_start=1
BASE COUNT     6509 a   3846 c   4297 g   6992 t
ORIGIN
        1 acgacgttgt aaaacgacgg ccagtgaatt gtaatacgac tcactatagg gcgaattcga
       61 gctcggtacc cggggatccc acccttatgg caataaaaga tgcaattgaa aaaattgttt
      121 catattttga tgccgacgaa gtaacagatc atgaagatgt tgctaaagaa cgtccagtta
      181 aagtacagaa aactgagcag acaccacctc aacagcaacg taaacctgag cgtcctcagg
      241 aaacagtccc tcctcgtcgc caacatatca aatcagacgt acagaaaact caagtccttc
      301 gtagtttacc tttgagtcga tcacaagtta atcagggatc ccaacaaatg aatacaacta
      361 aaactactat tgcaattaaa tatcctaaaa aatatgaaga tgctcaagaa attgtagaac
      421 ttttgattga aaatgaatgt gttttaattg attttcaata tatgttagaa gcacaagctc
      481 gtcgttgttt agattttatt gatggtgcta gcaaggtact tacaggaaac ttacaaaaag
      541 ttggttcttc catgtacttg ctcacaccaa tcaacgttgt tgttgacatc gaagagatta
      601 gtttgagtca tggaaatcaa gaatctactt ttgacttcga catgaagaga cgttaagcca
      661 tgtttgtatt actatatgtt atttgggcac gtgttgttaa cttggtagag atactccttg
      721 taatctatgc tttgttatct tggtttccag gagcttatga tacagctctt gggaagacta
      781 ttagacaaat tgttcagcca attcttgcac catttcgtag gctgaatttg gtttttgctg
      841 gtattgattt ttcaattatc gcaattatac ttttgttaaa tttctctctt agtattctta
      901 aggttgtcct attttgatgt ttgaagcaaa aaatattgaa caacattact cgttagaaga
      961 acgtcttttt ttagaaaagg ttaggggatt ttgtcaatta gttgaagatc ggtatagccc
     1021 atatttgact gatttcttaa atcctcgtga aatccagatt gtcaaagatt tagcaaatta
     1081 ctataattta aaattctttg taagttcaac ttttgataac gaagagtatg gaagagtgat
     1141 tcttgctccg gattattatg agcttgatga agatgacttt gaaattaaac gattagagat
     1201 ttcttatgct agacaattta ataaattgac acatcccaaa attttaggcg cactgcttaa
     1261 tcaacttggt cttgaacgtc aagtatttgg agatgttatt cttgatgaag aaggccgagt
     1321 tcaatttaac attgcttctc atctagcaag ctatgcaata atgactatta ctaaaatcgg
     1381 tagggtttcg gttacgctta gagaagtaag taaagatgat tggatttcca atcaagaaaa
     1441 atattctcaa tcttttgtct tgctttctag tatgcgtctt gataatgtgt tatctacagt
     1501 attaaaaaca tcgcgttcaa atgctttaaa gctaattgct tcaggcaaaa tcaaacttaa
     1561 ttataggcaa attgagaaat ctgatcagat aatatctatt ggtgatatga tttcggttag
     1621 aggttttggc cgatttcgtc taggtcaaca agaaggaatt tctaaatcag gaaaagccaa
     1681 ggtcgtcatc gacagtcttc taagactaca aaagtaggta tttaatttaa aagcatcaga
     1741 gatgctaatg aaacttgagg agatctgtaa tgacaattat agttcttgat attatcgaaa
     1801 aatagtttac aactaaattt tgtggatata atgaacaaga agtagatgat tttctcgata
```

1861 ttatcgtaga tggttatgaa gatttagttc attgaaatcg tgaacttgtt gctcatgtaa
1921 aagaacttga agaaatggta aaaaataaag gaggcctgta atggcaatca cagcacttga
1981 tattaaagat aaacagttta ccactaaatt tcgtggatac aatgaacaag aagtagatga
2041 atttcttgat attatcatag atgattatga agatttagtt cgtgacaatc gtgaacttac
2101 tactcgtgta aaagagcttg aagaaaaatt ggcttatttt gatgaaatga aagaatcatt
2161 gagccagtca gttatcttgg ctcaagaaac agctgaaaaa gttaaagctt cagcagcaga
2221 tgaatcagct aacttgatta acaaagccaa ctttaatgcg acacgcttga tcgaagaagc
2281 taaatctaag gcatctgaaa ttcttcgtga tgctacagat gaagcaaaac gtgttgctat
2341 tgaaactgaa gagcttaaac gtcaaagtcg tgtttttcac caacgtcttt tggctgcagt
2401 tgaagttcaa cttagcttag caagtgctcc agagtgggag gaattgttgc aaccaacagc
2461 tatttatctg caaaactcag atgttgcctt taaagaagtt gttgaaaaag tgctggatga
2521 acatgttcca gattcaaacg atgccgcttc attcgatgcg actcgtcaat tcactcctga
2581 cgaaatggct gaacttcaac gtcgcgctgc tgaaagcaat aaacaagtta aagatttctc
2641 taattcagat gttgacgaaa caaatgatat tacttcaact ccagtatatt ttggtattga
2701 agattcattc gaaagtgaag tagctaattt tccaatttct gcacctgtag acgaggacaa
2761 tgattcggaa caagaagtaa ctgttgaaaa aaatcttaat gaaacacaaa cttttaaatt
2821 aaatattagc gaataagaaa aacaaaactt tccaaatata ttaacagcga gtgggagatg
2881 gtgtgagtcc tacattccct atttgggtag ttatcatttt ctagcattct agctgaatta
2941 agtaagctag aaggatggtc ctccttacgg acatttgagg gagcaatgtt acttgctcta
3001 aactaaggtg gtaccacgag ctttcgtcct tttttgatga aggctctttt tatttagtat
3061 aaaaaaaatt aaagaggaga tataatgaaa cttaaagaaa cattaaatct aggtaagaca
3121 gcttttccaa tgcgtgctgg acttcctaat aaagaaccaa tctggcaaaa agagtgggaa
3181 gaagcgaaag tgtatcaacg tcgtcaagag ttgaatcaag gtaaaccaca cttcacactt
3241 catgatggcc ctccttatgc caatggtaat attcacgtgg gtcatgcgat gaataaaatt
3301 tctaaggata tcattgttcg atctaaatca atgtctggtt tctatgctcc atttattcct
3361 gggtgggata cacatggttt gccaattgaa caggtattgg ctaagcaagg cgtaaaacgt
3421 aaagaacttg atcgcgctga ataccttaag atgtgtcgtg attatgcact ctcacaagtt
3481 gataagcaac gtgaagattt caaacgtctt ggtatttcag ctgattggga aaatccttat
3541 gttaccttga caccagacta tgaggcagcc caaatccgtg tctttggcga aatggctaaa
3601 aagggttata tctatcaagg tgcgaaacct gtatactggt catggtcatc agaatcggca
3661 cttgctgaag cagagattga atatcatgat ttggtttcaa catctcttta ctatgccaat
3721 aaggttaaag atggtaaagg tgttttagat actgatactt atattgttgt ttggactaca
3781 acaccattta cagtgacagc ttcacgtggt ttggctgtag gtgcagatat tgagtatgtt
3841 cttgtaaaac ctgctggaga aactcgtaag tttattgttg cttctgaatt gctaaatagt
3901 ctttctgaga aatttgcttg ggaagaagta gaagttctta atacttaccg tggtgatgaa
3961 ttaaatcaaa tcgttacaga gcacccttgg gacagtgaag ttgatgaact tgtaattctt
4021 ggtgagcatg ttacaacaga ctctggtaca ggtatcgtcc atacagctcc tgggtttggt
4081 gaggatgact ataacgtagg tgttgctaat ggtcttgagg ttgtagtaac tgttaatgaa
4141 cgtggaatca tgatggaaaa cgccggtcct gattttgctg gtaaattcta cgataaagta
4201 gctccgattg ttatggagaa acttggagat ttacttcttg cgaaagaaga aatctctcac
4261 tcatacccat ttgactggcg tactaagaaa ccaatcatct ggcgtgcagt gccacaatgg
4321 tttgcatctg tttctaaatt ccgtcaagaa atcttggatg aaattgaaaa agttaaattt
4381 cattctgagt ggggtaaagt tcgtctttac aacatgattc gtgaccgtgg tgactgggta
4441 atttctcgtc aacgtgcttg ggtgttccca ctcccaatct tctatgctga agatagaaca
4501 ccaatcatga ctgaagaaac gattgaacat gtagctaaac tttttgaaga gcatggatca
4561 gttatttggt gggaacgtga tgccaaagat cttcttccag aaggtttcac acatccaggt
4621 tcaccaaatg gtgagttcac taaagaaaat gatattatgg atgtgtggtt tgactcagga
4681 tcgtcatgga atggtgttgt tgtaaaccgt ccagagttaa catatccagc tgatctctat
4741 ctcgaaggtt cagaccaata ccgtggttgg ttcaactcat cacttatcac ctcagttgcc
4801 aaccatggtg ttgcaccata taaacaactc ttgtctcaag gttttgcact tgatggtaag
4861 ggtgaaaaga tgtccaaatc tcttggaaat acaattgctc ctagtgatgt tgagaaacaa
4921 tttggtgctg aaattcttcg tctttgggta acaagtgtag atactagcaa tgatgttcgt
4981 atctcaatgg atatccttag ccaagtatca gaatcttatc gtaagattcg taacacactt
5041 cgtttcttga ttgctaatac ttctgacttc aatccaacaa ctgatgcagt agcatttgaa
5101 gatttgcgct ctgtcgatca atatatgact attcgtttta accaattggt taagaatatt
5161 cgtgatgcct atgaaaactt cgaattcttg actatttaca aatctttagt taacttcatt
5221 aatgttgaat tgtcagcctt ctatcttgat tttgctaaag atgtcgttta cattgagagt
5281 gctaaatcac ttgaacgtcg tcaaatgcaa acagtcttct acgatattct tgttaagatt
5341 acaaaactct tgactccaat ccttcctcat actgcagaag aaatttggtc atatcttgaa
5401 tttgaaaatg aagactatgt tcaattgtca gagttgccag aagcagaaga ctttgctaat
5461 caagatgcac ttcttgaaaa atggaatgcc ttcatggact tccgtggaaa agctcaaaaa

5521 gctttggaag aagctcgtaa tgaaaaagtt attggtaaat cgcttgaagc tcatttaact
5581 atctatccag atgctgaagt aaaagaactt ctcgaaagtt tgaatactaa ccttgctcaa
5641 cttttgattg tttcagctct aactattgct gaaggagatg ttcctgaatc tgctgtaaga
5701 ttccaaggtg tttcattcac agtagaacgt gctgaaggtg aagtatgtga ccgttgtcgt
5761 cgtattgatc caactactaa agaacgtagc tacaacgcga ctatatgtaa tcactgtgct
5821 agcattatcg aagaaaactt tgctgaagtt gtggcagaag gtttcgaagt tttaaaagca
5881 aaataagcta aaaagagttg ctctttgtta gctgtagtgg gtgacgaaaa gataacatct
5941 agagaggacc atataggtct tctcttttg aatgttcaga gtgatgaaaa cacgcttcct
6001 aaaggtgata gaggttgtga taattagcga actcttctga gaaatccagt gtcttctcaa
6061 ggacttcatg tgggtttaag gtttgacaaa aggttttcga atgaaatgat ttacgagata
6121 acttacggct gtccttttga gagagtcacc agtgattttt taaatctcaa taaggtagtg
6181 aatttttatt aaactggttc ataatggcaa ttcttgtctt taaaaaggct cgatcaagct
6241 gctagatgat gttgaaacga tcaataatga tttgggcatt tagaaaaagt cttcgaactt
6301 agtggcatat aaagctcctg acatatccat cgtgataaac cttacctttt ggcgtacttt
6361 caatggatac ttcaaaaaga agtttcgact agtggtttgg cggcgattat cgaggatggg
6421 tatgagtttg ttgttctcat agttttgagc cacaaaagcg aattcacctt tcttgaaact
6481 aaactcatcc caggacataa cagctggtag tttgtcataa tgttccttga aattaaactg
6541 gtcaagtttg cgatagacag tggacgtcga tacatgaagt cttctggcaa tatccgtaag
6601 tgatatcttc tcagtgagga gttgtgagac cttttgtcgg actagatttg aaatttggtg
6661 gtttttctca acgatatatg tctcagaccc cgtgacttgc ctacaacttt cacactggaa
6721 gcgacgcttt ttaagacgta gtagtattgg tattccagtt ttgcttgaac ctcgatatga
6781 atatccgttt caaaaacaag agaaataatg atgttagggt ctttaattcc gattaatttt
6841 gtggtattct tcataagtct cataagttct tcctaatgat ggttttgtcg ctcttcatta
6901 tagttcttat gggacttttt gtgtatactt aaaaagctct ataatctcta cagtggtttt
6961 acccactaca gaaattatag agcctctaat cgttgcttgc gtgcgtccgt cgaggattgt
7021 gatgactttt aatgagtcga aatcctgagc gatgaaactc atttttccct tcttgaagcc
7081 gtactcatcc cagctcatat tctcaggcag ccaattccag tcggttttaa attggaactc
7141 atttagcttg cgactgacgg ttgaggtaga gacggcaagg cgcttggcaa tgtcggtcat
7201 agactgtttt tcaatgagca attgagcgat tttctgatag acgacggttg cgatttggtg
7261 attcttcttg accagagaag tttccgcgac agccattttt ccgcactcct tacacttgaa
7321 acggcgcttt ttcaggcgaa tgagagttcg gtagccagca cactccagat acgggatttt
7381 agaggctttc tggaagtcgt atttgcccat ctgtcccttg caggcaggac atttaggaga
7441 ctcgtaatcc aagtaaccgt gaagttcttt gtgcgttccc atatcgtatt cattagtgat
7501 gataatattt ttgtctttta ttccaagaaa atttgtgata agatttagtt gttccatatg
7561 attctttcta aaatgatggt ttagtcgctt ttcattatag gtcatatggg acttttttt
7621 ctacaatcaa aaaggctcca taatctccat agaggattta cccactacag aaattataga
7681 gccggaatgt tatgtagttc aggctctttg tcaactgtag tgggtagatg aaaagctaaa
7741 ttcttgagag gaccagtttg gtcttctctt ttttaatatt tactgccatc aaaatccgtt
7801 tcttaaagtt ttcaaagttc ctgaagccaa aggcatttcg cttaatgacc ttgatgagat
7861 tgttcgttgc ttccagtttt gcgtttgaat acggcagttc gagggcgttg ataatcttgt
7921 ctttatcttt caaaaaagtt ctaaaaactg tctgaaagat agggtgtacg ctggaactcg
7981 tctcttcaat gaggtcaaag aaatggtccg agttcttctc ttggaaatgg aaaagtaaaa
8041 gttggtagag ttcataatgt tctctcagtt cttgagagta tgacagtagc ttttctagga
8101 tttccttgtt ggttaaatgc gcacgaaaag tcggacgata aaagcgttta tcgttgagtt
8161 tgcgactatc ttgttggatc agtttccagt agcgtttcag ggcacgatat tcctgtgact
8221 gacggtccaa atggttcatg atttgaatgc ggacgcggtt catagcacga ctaaggtgtt
8281 gcacaatgtg aaagcgatca aggacaatct tagcgttagg gaataatttt ctggcgatgt
8341 cgtaataagg gctaaacatg tccatagtga tgactttaac gcgatttcta tcctgtctgg
8401 aatagcgtag gaagtggttt ctaatcgttg cttgcgtgcg tccgtcgagg attgtgatga
8461 cttttaatga gtcgaaatcc tgagcgatga aactcatttt tcccttcttg aagccgtact
8521 catcccagct catattctca ggcagccaat tccagtcggt tttaaattgg aactcattta
8581 gcttgcgact gacggttgag gtagagacgg caaggcgctt ggcaatgtcg gtcatagact
8641 gtttttcaat gagcaattga gcgattttct gatagacgac ggttgcgatt tggtgattct
8701 tcttgaccag agaagtttcc gcgacagcca tttttccgca ctccttacac ttgaaacggc
8761 gctttttcag gcgaatgaga gttcggtagc cagcacactc cagatacggg attttagagg
8821 ctttctggaa gtcgtatttg cccatctgtc ccttgcaggc aggacattta ggagactcgt
8881 aatccaagta accgtgaagt tctttgtgcg ttcccatatc gtattcatta gtgatgataa
8941 tatttttgtc ttttattcca agaaaatttg tgataagatt tagttgttcc atatgattct
9001 ttctaaaatg atggtttagt cgcttttcat tataggtcat atgggacttt ttttctacaa
9061 tcaaaaaggc tccataatct ccatagagga tttacccact acagaaatta tagagccctt
9121 tttatgtatg ttcagtgtga tgaagacacg tttcttaaag ttgatgaagt ttctaaaacc

9181 gaagcccaag cgtttaatgt ctttaattaa cttattagtc gcttcaagtt ttgcgtttga
9241 atagtctgtt tctagtgcat tttggatgta ttgcttgtgt ctaagaaaag tcctaaagac
9301 cgtttgaaaa tagtgattga ccttgcttct attttcctct atcaggtcaa agaactcacc
9361 tactctcttc tcctgaaagt gaaaaagcaa aagctgataa agtgtatagt agtcagtaag
9421 ctcttttgaa aagactagtg tcttcttaac aacttcatgt ggtgctaaag tttggcggaa
9481 agtctttgaa tagaaagagt tgagagataa cttacagctg tccttttgga agagtcgcca
9541 gtgatttttc aaggctcgat agggtagtga cttcttatcg aactggttca taattgcaat
9601 tctagtcttt aaaaaggcac gtccaaggtg ctggatgatg tggaaacgat caagaacgat
9661 ttttgcgttt ggaaagagct tgcgagccag tggaatataa gctccagaca tatccatcgt
9721 gataaactgt acctgttgcc ggactttcaa tggatacttc aaaaagtagt ttcgtatagt
9781 agtttggcgg cgattatcaa ggatggttat gagttggttt gtctcataat tctgcgccac
9841 aaaagccaat tcccctttct tgaacccaaa ctcatcccag gacataacag cagggagttt
9901 gtcataatgt tccttgaaag tgaactgatc aagcttacga tagacagtgg acgtcgacac
9961 gcgaagtctt cttgcaatat cagttagtga caccttctca gttagaagct gtgtaacttt
10021 ttgtcggact agattggaga tttggcagtt tttctcaacg atagatgtct cagctaccgt
10081 gactcttcta caacttttac actggaaacg acgttttttt cagacgtagt agagttggcg
10141 ttcccgcttg ctcgagaaga gggattttag aaggcttttg aaagtcgtac ttgatcatct
10201 ttccatggca gtgaggacat gatggtgcag ggtaatcgag ttttgcttga atctcgatat
10261 gagtatcagt ttcaaaaaca agtgaaatct tgatattttg gtctttaatt ccgattaatt
10321 ctgtggtatt cttaataggt ctcataagtt cttcctaatg gtagttttgt cacttttcat
10381 tatagttctt atgggacttt tagtgtacac tcaaaaagct ctataatctc tacagtagtt
10441 ttacccacta cagaaattat agagccgttc tgtattgcac aggaccaagt ccttttaatt
10501 ttaccttaat gcgtttgttg ttgtaataat caatatagtc tacaatagct tgttccaatt
10561 gctcaagtga gtgaaacatc ttctcgtaac cataaaacat ctcagactta agaatatcaa
10621 agaaagactc catcatacca ttgtctggac tatttccctt acgtgacatg gatggttgga
10681 tccctttatc ctctaaaaat tgatgataat actgatgttg gtattgccaa ccttaatcac
10741 tatggagaat tgtattttca taatgctcct ctgtgaagga ctggtctaac atagatttca
10801 cttgttctaa gtttggtgaa gttgagagat tataagcgat aatttcgctg ttgaaaccat
10861 ctaaaacagg cgataaatac aatttatgtg tgctatttgg aatggcaaac tctgtcacat
10921 ctgtgtagca cttttccatt gggttggctg cttcaaactg ccgttggata agggttatct
10981 gctttcttgc caacttctcc ttggtatgaa gaatacttgc gtttacgaca aatttgggaa
11041 cttaaaccaa ggagcttcat cagacgtttg accttcttat ggttcactac aaaaccacga
11101 ttcctcaatt caagagttat tcgacgatat ccgtaatttc ccttatgttc atcataaatg
11161 gcttgtattt ccactttgat agattgattt ttatctactt gatttagttt ctttaagtgg
11221 taataatagg ttgaacgagc aagtttgata atgtttagaa ggatatctaa aggaaactct
11281 gttaccaatc cttgaacaat ttctgttttt cttcttgctc cttttcgtcc ctcaatcgga
11341 gttatctcaa ctttttaga atagcattct ccgctctcag gtactcattt tctgcttgaa
11401 gacgttctaa ctctgtcatc tcttcgggtt tcttctttgg cttactaccc atttaggta
11461 ctctccctcc tcttttctca agaagagtat acccattttt cttgtattgt gctatccaat
11521 tgttaagcaa cccttaattt gagaggccat agtccaggca aacactttgt tgagaacaac
11581 cttcaagtag aactttatca atcatttctc gctttaattt aggagaataa taacgattct
11641 ttccctttt gatgatgttt attccataat ggtcaattaa tttcaccata tattttagac
11701 cagaaacgtt caccccaaat tgatttgaaa gtcgtttaaa gctttctcct tgatttctca
11761 attcatatat ttgaacttta tcttcataag ttagtttcat aataaaaaca ccccaaaagt
11821 tagattttt tgtttttgtc taacttttgg ggtgcagttc atttaaaacc ttttttgtta
11881 tagagcaata atagaataat attgctctta tttttcatag gttaccttta gaaatttaga
11941 aaaaggattg gaagtaaaac ctagtccttt ttatcacttt gaagatttgc aatacctcat
12001 atcttatttg tttttagtta gagcttttcg aatgaaatct gctggtataa tcaatacagc
12061 aataagaaga gcaatgatat agtgttgaat atccatcgga acagtactaa atacttttcc
12121 accaaattga ataataaggg tttgaacaac agcaatagaa atcataacga ttaagaattt
12181 cttgttttta ccgatatgtt caaaaatatt gaagccatta ctacgtgtat taaatgagtt
12241 gaaaataaca gcatagataa atactgtaaa tgtaaaggtt gttaccattt caaagttacc
12301 agttccattt gtgataaagt cttggatacc accaacattt ttaagaatgg ctaaacatac
12361 aagagtaatg aaggcacttg caactccgat agcggatttc atataaccag taagaatatt
12421 tgctttctta gctactggtt tttcgttcat ataacgatct agcgcaggct cttcaccaaa
12481 ggcaagggca gcaagtgtgt ccatgataag gttaacccag agaatttgga tgattgtaaa
12541 tggctctttg agacccaaaa gtggcgagag aagtgacatg gcaatagttg ttacgttaac
12601 agttaattgg aagatgataa atttacttac tgacttactc atggtgcgtc catagagaac
12661 agctttctca atagaagtca atgagttgtt taagataacg atatctgagg cttcacgtgc
12721 gacttcagta ccgtcaccca ttgagaaacc aacatcagct gatttaaggg ctggtgagtc
12781 attgacccca tcacccgtca taccagctac catatcgagt tcttgagctg cctcaataag

12841 acgtttttta tccattggaa gggcacgact taccactttt aagtgaggca attgttgttt
12901 aagttcttga tctgagaggg cagaaagttc atcgtgagta aggacaaggt catttctcc
12961 agtgacgata cctgcttctt tagcgatagc aacagcggtt tctttacggt caccggttac
13021 cataactact tgaacacccg cacggttcat tgtttcaacg gtttgtttaa ttgaagagcg
13081 aacattatca cggatacata cgataccaat caagacttta tcattaccat ctgtattgag
13141 gatggcaagc aaacgcattg aacggttagc ttgttccaat gataactctt gaaaacgagc
13201 tttaatatca tctgtaaagt tttgtttatg accatcttta tcaagatagt agtagcagtc
13261 atttaggata aattctggag cacctttgat gtatattttt ccatcgttag tcgttacact
13321 tgcgaatttt gttgctgagt tgaattgttg cttctcagta attgtattag tatcgaaatc
13381 aagttgagaa cgtccaatca agaagtcaag aagtgcacgg tcagtagcat tactaccaac
13441 agcattacca tcagcaacca tggcatcgtt attaagaccg ataccattaa ttatttcagc
13501 tttaagacta tctgacatcg tatcaaaatc tggagcatcg gtttcaccag tagctgcata
13561 aagtgttcca tccccaagga agaagtcaac tacagatagt ttaccttctg tgattgtacc
13621 agtcttatca ctgaagagaa tattcatgta tccagcagtt tcaatagtat caggatggcg
13681 gactaggata ttttgagcta aaagtcgacc tgagttcatt gaggaaacga gtgctaacat
13741 cattggaagt ccttctggga cagccatgat gatgattgta accgcaaaga gaattgtttc
13801 aatgataaga aggaaaatcg agccaccatt gaggttgtta gctttattta gggcaataaa
13861 gcctaataca aggttgatta ctgaataggc agcacctgca ctataaccga ggaccccgat
13921 gtttcttgca agcttgttaa gtttttcttt agatggtgaa tttttactat cttcttgaag
13981 agacgtattg atacttccta aaacggtgtt atcaccaatt tgtgtagctt caatcacagc
14041 ttcatctgaa gttacgactg taccacggaa gaccttaaac tccgtaaaga ggtctgaaga
14101 atctggctct tctcggtcac caagaggaag ttttgtggca tcctcgcttt caccatttaa
14161 gacagcttgg tttactttta atttaccttc caagatgata ccgtcaactg gaatcttgtc
14221 ccctgattga aggagaattt gatccccttt tacaacatca tccacgagaa tctctttaag
14281 tttaccatct cgataaacct tagcattagt tttgaagcc tcttcttgga gaatattgaa
14341 tttttgctca ttacggtatt gagagactgc tgagaaccct gtagacatca aaatggcaaa
14401 aattagtgag atagcctcat accaattagc ctccccaata gcaggaaaaa tcattgcaac
14461 aaagttgaag atgattttta atcctaaggc agccaagagg atgagaatcc attgatcctg
14521 gaaggcttca ataaaaattg aaagcagcga gttagcctct ttactagaga gcctattatc
14581 gccatgagcg tttttacttg cttgaacttc tgcagaagtc aagcctttaa attttttcat
14641 taaacgtatt tatcctttct agaatgttca aaaatttaaa acctaataga agcagtaatt
14701 ttaataacta tcagtaagta ttatacttta ttttactatt aaattgttga tttttttatca
14761 agagaagtag tgattgattt ctagaaaatg aaatttcaca attttaaatc catattttag
14821 aaatctaccc tcaattcata caaccctctc acagcattac agccatagat ggcttgagcc
14881 tgttttaaat cttccagagt caaaactttc tctgtgacct gattattagc aatgaggctt
14941 tgtctgtaaa taccgtttaa aagaccgagg tgaacaggtg gagtgtagag ttggtccttg
15001 attttaagaa cgaggttacc gatagaagtt tctaaaagtt ctttcttttg attatagtag
15061 atttgctcat gaggtcctag actaatgtgt ggtcgataac ttgttttaaa gtaggtatag
15121 gggttattca aaggatgtgt ttgcttaact aatcttgctt gacaaaaatc atcgtctaat
15181 tctgttaatt gagcatgttc aaaagtaagt ttaccatctt ttgcaaggga caatttaaga
15241 cggtagtctg tgtcaaaatc aagggactga cacaaatctt cgacttgatt ttgaacttct
15301 tttttattga aaggataagc aaaatagcga ctggactctt gtagacgatt gagatgttct
15361 ttaagatgga gtagtttacc ttgatgaatc cgtccagttg agataagatc aaatctagga
15421 ttttgacggt atagaatagc tgatttttgc cttgtttctt catattcagc ttcccatttg
15481 ctgtcccagg tgattccacc gcctactcca tatatagcat tgtttccctg catttgaagt
15541 gttcggatgg ctacgttgaa aatagttggt ccattaggta gcaagatacc aatggctcca
15601 caatagacgc ctcgagcttg tttttcgacg tttttaataa ttgccatagt agctattttt
15661 ggtgctcctg taatagatcc acaaggaaaa agggcttgga agatgtcatc caaacgactg
15721 tttggtagga gttgcgtctc aatagttgaa gtcatttgcc aaacagtaga gtattgttca
15781 acctggcaaa gtctttttac atttcactc cctattttg aaatacgatt catgtcattt
15841 ctaagaagat ctacaatcat catattttca gagcgatttt tctgatcatg agcaagccat
15901 tgtgcttgtt taaggtcagt ttcagttgtc aagccacgat ttgttgtccc tttcatagga
15961 cgtgtggtca atatatcacc atcttttta aagaagagtt caggacttat ggaaatgatg
16021 gagacatcat catgttgaat aaaggcattg taatgtgcat tttgctcaac aaccaatcgg
16081 ttgtagatgg caaatggatc agctgttatg ttctgttgaa gttggacggt aaaattgacc
16141 tggtaggtgt ttccttgacg aatatggtgg tgtatatgct caatagcagc cttgtattcc
16201 tctgcagaag ttagttcttg ccaagattct ggtaagggaa caggctcata agcaagaggg
16261 atagactctg tttgaacagt atcgtgaata gtaaaataga gaagatactc tgacattaat
16321 gggctatcaa aaatttgaaa tttaggttca aaggcctgag aagcttcata ggctacatag
16381 cctacagcaa aatagccttt ttcttgatag gcttctattt gatttataag tgatttaact
16441 tccttaatgt ctttggtttt tatctctttt atggcgtgag tgaagatttg tctgacgcca

16501 agttctttaa aatcaataac ggttttctta tgcatacata gagtataaca taagaagata
16561 gcctctggtt ctttttcatg atttaattct ctttcttaaa attttaacca aaaatgaact
16621 tttcagatag ttttatatat gttataataa tatgaactaa aagaagattt tagagggaaa
16681 tatgactaaa aaaaaataaa tttgagaaag gggacagtag tctttcaaat aaaaaaattg
16741 aaaaaagtga cttccaagcg gaattcttgg aaacatcaaa tgtacgagct tgagattgcc
16801 attgcaaaag agtcaagcaa taaaaagaga gacgagctta taaaacttca taatggctat
16861 atgagtgagt atgatagttt aaagaagcaa atcatttggt tagcagtatt gggaatatgt
16921 ttcctagtgg tcttggtgat tttgggctat tttatttgga atagctataa cagtgagaac
16981 atgtcctata catcttcttg gcttcaagaa cgagtttaag atttagtttt gattaaaacg
17041 ccttatttta tgactttact acgcaatagt gaacatagat gagttgatta ctaaatatta
17101 aagaagaaaa agtaagatat atctctagac aaaagtctag agtttttttt tgataaaaat
17161 gaccttatca tctccctata tagttattag ctatacaaat atctaattaa aaataagaca
17221 acgataccag aaatccttta ttttatagga tatagtttaa aactatatat attgaaaact
17281 gataacaatt atacagatat ttatttcttt ggtaagatta aatcaacaac aaagaggagg
17341 acacaagatg tcaactacta tcattggttt cccacgtttg ggtgaattcc gagaattaaa
17401 atttactaca gaaaaatatt ttagaaatga aattactgca gacgaacttt tagcggctgc
17461 taaagatttg cgtgcaaaac actggaatat tgttaaagaa aaaggtatta ctgaaattcc
17521 ttcaaatgac ttttctcatt acgataactt ccttgatgcg gccttccttt tcaacgtagt
17581 tcctgagtct gtgaaaaact tggatttgac tgaattggaa caatactttg ctttggcacg
17641 tggttaccaa ggtgaaaagg gggatgtgcg tgctcttcca atgaagaaat ggttcaacac
17701 caactaccat tacatcgttc caaaatttga aaaaacaaca gcagtaaaat tggctggtca
17761 caagattttt gatgagtacc aagaagccaa agacttgggt ctggatactc gcccagttgt
17821 ggttggacca ttcactttcc ttcaattgtc tgatttcgaa gatggtgtga aagcagaaga
17881 cttcgtcgat agctttattg ctgcctacca agatgtcttt gcaaaattgg ctgaacttgg
17941 tgctactcgt atccaacttg atgaaccagc acttgttaaa gatttgactg ctgacgaaaa
18001 agcactcttc ttgaatctgt acaataaaat cttggctgac aagaaaggcc ttgaggtctt
18061 gattcaaact tactttggtg atgttcgtga tgtatacaat gatcttgtaa acttgccagt
18121 agatgctatc ggtcttgact ttgtcgaagg taagaagtcg cttgaacttg tcaaaggtgg
18181 attcccagct gataagactc tctatgcagg tattgtcaat ggtaaaaaca tctggcgcaa
18241 caactacgaa aagagtttgg ctgttcttga acaaattcca gctgaaaaaa tcgtattgac
18301 aagctcatgc tcacttcttc atgtgccatt tacgactgct aatgaagaat ttgaaccagc
18361 aatcttgaac cactttgctt ttgccgttga aaaattagat gaacttcgtg atttggatgc
18421 tatccgtaat ggtcaaggtg ctgaatcact tgctgctaac aaagagctct ttgctattga
18481 acgtgttggt gcaaatgctg aacttcgtgc tcgtattgca ggtttgacag aagcagatta
18541 cacacgttta ccggctttcg cagaacgtga agccattcaa aaggatgcct tcaaacttcc
18601 attgcttccg acaacaacta tcggttcatt tcctcaaact aaggaagttc gtgccaaacg
18661 tttggctttc cgtaagaatg aattgtctca ggaagaatat gatgcctttc ttgctgaaat
18721 tatagatgaa tggattaaat ggcaagaaga agttggcttt gacgtccttg tacatggtga
18781 gtttgaacgt aatgacatgg ttgagtactt cggtcaaaac ttgtctgggt acctcttctc
18841 taaaaatggt tgggtgcaat catatggtat gcgtggggta aaaccaccaa tcatctgggg
18901 tgatgtcact cgtcttaatc caatcactgt aaaatggtct agctacgcac aaagccgtac
18961 tgacaagcct gttaaaggta tgttgactgg gccagttaca atcctcaatt ggtcattccc
19021 acgtgaagat atctctatta aagattcaac tcttcaaatc gctcttgcta tcaaggatga
19081 agtgcttgac ctcgaagctg caggcattaa aattatccaa atcgatgagg ctgctcttcg
19141 tgaaaaattg ccactccgtc gtagtgactg gtacgaagac taccttgact gggctattcc
19201 agccttccgt ttggtacact caacagtagc accagacaca caaatccaca cacacatgtg
19261 ttactcagaa tttacagata tcattccagc tatcgacaac ttggatgcag atgttatctc
19321 ttttgaagct agtcgttcaa accttgaaat tttggatgaa cttaaagcac aaaacttcca
19381 aacagaagtt ggacctggag tttatgatat ccactcacct cgtgtcccac aagacggtga
19441 aattgaccac actatcgaag cgatcttggc taaagttcca agtagtaaag tatggatcaa
19501 ccctgactgt ggtttgaaga ctcgtggtat taaagaaact aaagaaagct taaccaagct
19561 tctagaagca gcaaaagcgg ctcgcaaaaa cttgtaagta gaagttataa taccttgaaa
19621 agcttctttc ctatatgtag aaggttttca atagtgaata attttctaaa gatttttgta
19681 atcaagctag atgcaaaatg tgatcaataa ttcctacgag tttacttgac actaataaaa
19741 atgcttttgt ctcaacaaaa atctaaaacg aagaaggtaa aacttatgac aagccaaaca
19801 ccatccctct cttttgaagt ctttccacct aatccagccg taggcaataa gaaaatcttg
19861 cttgcccttg atgacatgaa aggatcggca ccccatttta tcagtgtaac agctagtaat
19921 aacaaatata atatcaaaga gacaacggtt cctttggcag accatattca aaatgacctg
19981 gctattccta ctattgctca ccttccagca atctatttga gcaaagaaaa ggtagctgat
20041 actttgcgag aattagatgc tgttggagta aatcgtatcc tggcacttcg tggcgacatc
20101 attcctgggg tcgaaccttt aaaagacttc agatatgcaa ctgatttgat tgagtttatc

```
20161 aaggcagaag ctcctaactt tgatatcatt ggtgcttgtt atcctgaagg tcatcctgat
20221 tcaccaaatc aaatctctga tattcaaaat cttaagaaga aagtagatgc aggttgttca
20281 agtcttgtaa cccaactctt ctttgataat gaacgcttct acgatttcca agataaatgt
20341 actcttgctg gcattaatgt gcctatctat gctggtatta tgcctatttt gaatcgtaac
20401 caagccctac gtttacttaa gacgtgtgaa aatatccatt tgccacgtaa attccgtgct
20461 attttggata agtatgagca tgattctgaa tctctgagag ctgccggtct agcttatgct
20521 gttgaccaaa tcgtggattt ggttacgcaa gacgttgctg gagttcacct ttatacgatg
20581 aataatgcag agactgcacg tcatatttac gaagctactc actccttgtt taagcatcat
20641 tcacaagtcg gcgcactgta attgaataat agaaaaacca cctatatatt taatctatag
20701 gtggtttctt ggtgtagcag cgaaagattg taattactta tatggggcat tattatcatg
20761 tatcaaaaaa gacctagagc ttagctctaa gtcaaaagta ttattcacct acaaaagcat
20821 tgatttctgc ttcgatgtta gcaatttttt tctttagcat ccgcttcagt ttctccaact
20881 gttgcaatgt aaaatttgat ttttggttct gtacctgaag gacgaacggc aaaccatgaa
20941 tcatcagcca agatgtattt caaaacgtta cttggaggag ttgtcaattt ttctacgccg
21001 tcagcagtag tagctgtttg ttccaagaag tcttctgttt tagcaatatc agtgttgttg
21061 aattgtttag gagcattgcg acggaatttg tccatgattt tcttgatttc tgcagcacca
21121 tcaacacctg aaagcgtaac tgaaattgtc ttttctgaga agtaaccata ttgtttgtag
21181 atttcttcga taccatctgc caatgtcata ccacgtgaac ggtagtatgc agcaatttct
21241 gcaacgataa gaacggcttg gatagcgtct ttatcgcgta caaatggttt gatgaggtaa
21301 ccgaagcttt cttcaaaacc aaacatgtaa gtgtaattgt gttgtgtttc aaattcatga
21361 atcttttcac cgataaattt gaagccagtc aagacattaa acattgttgc gccgtagctt
21421 tctgcaatct tagtaactaa ttcagttgat acgattgatt tacaaagggc agcattagca
21481 gggagagtac cagctgtttt gtgagcttca aggatatatt tggcgatgat agcaccaatt
21541 tggttaccag aaaggttgag gtatgaacca tctggttggc ggatttcaac gccaagacgg
21601 tccgcgtcag ggtcagttgc aaccaatacg tcggcatcta catt
//
```

Kan10

```
LOCUS       10K
DEFINITION
FEATURES             Location/Qualifiers
     gene            153..980
                     /rbs_motif="None"
                     /uscore="3.51"
                     /rscore="-3.27"
                     /sscore="-3.23"
                     /conf="100.0"
                     /cscore="135.48"
                     /partial=0
                     /tscore="-3.48"
                     /rbs_spacer="None"
                     /gc_cont="0.407"
                     /start_type="GTG"
                     /score=132.3
                     /locus_tag="10K_01"
     CDS             153..980
                     /rbs_motif="None"
                     /uscore="3.51"
                     /rscore="-3.27"
                     /sscore="-3.23"
                     /conf="100.0"
                     /cscore="135.48"
                     /partial=0
                     /tscore="-3.48"
                     /product="4-alpha-glucanotransferase"
                     /rbs_spacer="None"
                     /gc_cont="0.407"
                     /start_type="GTG"
                     /score=132.3
                     /codon_start=1
                     /locus_tag="10K_01"
     gene            1060..3246
                     /rbs_motif="None"
                     /uscore="8.28"
                     /rscore="-3.27"
                     /sscore="1.37"
                     /conf="99.99"
                     /cscore="285.85"
                     /partial=0
                     /tscore="-3.65"
                     /rbs_spacer="None"
                     /gc_cont="0.379"
                     /start_type="TTG"
                     /score=287.2
                     /locus_tag="10K_02"
     CDS             1060..3246
                     /rbs_motif="None"
                     /uscore="8.28"
                     /rscore="-3.27"
                     /sscore="1.37"
                     /conf="99.99"
                     /cscore="285.85"
                     /partial=0
                     /tscore="-3.65"
                     /product="maltodextrin phosphorylase"
                     /rbs_spacer="None"
                     /gc_cont="0.379"
                     /start_type="TTG"
```

```
                     /score=287.2
                     /codon_start=1
                     /locus_tag="10K_02"
     gene            complement(3352..3792)
                     /rbs_motif="None"
                     /uscore="1.42"
                     /rscore="-3.27"
                     /sscore="1.22"
                     /conf="100.0"
                     /cscore="44.7"
                     /partial=0
                     /tscore="3.07"
                     /rbs_spacer="None"
                     /gc_cont="0.306"
                     /start_type="ATG"
                     /score=45.9
                     /locus_tag="10K_03"
     CDS             complement(3352..3792)
                     /rbs_motif="None"
                     /uscore="1.42"
                     /rscore="-3.27"
                     /sscore="1.22"
                     /conf="100.0"
                     /cscore="44.7"
                     /partial=0
                     /tscore="3.07"
                     /product="hypothetical protein X841_05855"
                     /rbs_spacer="None"
                     /gc_cont="0.306"
                     /start_type="ATG"
                     /score=45.9
                     /codon_start=1
                     /locus_tag="10K_03"
     gene            complement(3734..4402)
                     /rbs_motif="None"
                     /uscore="6.14"
                     /rscore="-3.27"
                     /sscore="5.28"
                     /conf="100.0"
                     /cscore="64.59"
                     /partial=0
                     /tscore="3.07"
                     /rbs_spacer="None"
                     /gc_cont="0.332"
                     /start_type="ATG"
                     /score=69.9
                     /locus_tag="10K_04"
     CDS             complement(3734..4402)
                     /rbs_motif="None"
                     /uscore="6.14"
                     /rscore="-3.27"
                     /sscore="5.28"
                     /conf="100.0"
                     /cscore="64.59"
                     /partial=0
                     /tscore="3.07"
                     /product="hypothetical protein"
                     /rbs_spacer="None"
                     /gc_cont="0.332"
                     /start_type="ATG"
                     /score=69.9
```

```
                     /codon_start=1
                     /locus_tag="10K_04"
     gene            4585..5574
                     /rbs_motif="AGGAG"
                     /uscore="3.46"
                     /rscore="11.88"
                     /sscore="18.4"
                     /conf="99.99"
                     /cscore="178.52"
                     /partial=0
                     /tscore="3.07"
                     /rbs_spacer="5-10bp"
                     /gc_cont="0.384"
                     /start_type="ATG"
                     /score=196.9
                     /locus_tag="10K_05"
     CDS             4585..5574
                     /rbs_motif="AGGAG"
                     /uscore="3.46"
                     /rscore="11.88"
                     /sscore="18.4"
                     /conf="99.99"
                     /cscore="178.52"
                     /partial=0
                     /tscore="3.07"
                     /product="lipoate-protein ligase A"
                     /rbs_spacer="5-10bp"
                     /gc_cont="0.384"
                     /start_type="ATG"
                     /score=196.9
                     /codon_start=1
                     /locus_tag="10K_05"
     gene            complement(5571..5693)
                     /rbs_motif="AGGA/GGAG/GAGG"
                     /uscore="0.29"
                     /rscore="2.01"
                     /sscore="3.12"
                     /conf="67.3"
                     /cscore="0.02"
                     /partial=0
                     /tscore="1.47"
                     /rbs_spacer="11-12bp"
                     /gc_cont="0.301"
                     /start_type="ATG"
                     /score=3.1
                     /locus_tag="10K_06"
     CDS             complement(5571..5693)
                     /rbs_motif="AGGA/GGAG/GAGG"
                     /uscore="0.29"
                     /rscore="2.01"
                     /sscore="3.12"
                     /conf="67.3"
                     /cscore="0.02"
                     /partial=0
                     /tscore="1.47"
                     /product="hypothetical protein V528_04795"
                     /rbs_spacer="11-12bp"
                     /gc_cont="0.301"
                     /start_type="ATG"
                     /score=3.1
                     /codon_start=1
```

```
                /locus_tag="10K_06"
gene            5808..6872
                /rbs_motif="None"
                /uscore="4.17"
                /rscore="-3.27"
                /sscore="-1.65"
                /conf="100.0"
                /cscore="77.3"
                /partial=0
                /tscore="-3.65"
                /rbs_spacer="None"
                /gc_cont="0.385"
                /start_type="TTG"
                /score=75.7
                /locus_tag="10K_07"
CDS             5808..6872
                /rbs_motif="None"
                /uscore="4.17"
                /rscore="-3.27"
                /sscore="-1.65"
                /conf="100.0"
                /cscore="77.3"
                /partial=0
                /tscore="-3.65"
                /product="site-specific tyrosine recombinase XerS"
                /rbs_spacer="None"
                /gc_cont="0.385"
                /start_type="TTG"
                /score=75.7
                /codon_start=1
                /locus_tag="10K_07"
gene            complement(7041..9581)
                /rbs_motif="AGGAG"
                /uscore="1.01"
                /rscore="11.88"
                /sscore="15.95"
                /conf="99.99"
                /cscore="460.66"
                /partial=0
                /tscore="3.07"
                /rbs_spacer="5-10bp"
                /gc_cont="0.404"
                /start_type="ATG"
                /score=476.6
                /locus_tag="10K_08"
CDS             complement(7041..9581)
                /rbs_motif="AGGAG"
                /uscore="1.01"
                /rscore="11.88"
                /sscore="15.95"
                /conf="99.99"
                /cscore="460.66"
                /partial=0
                /tscore="3.07"
                /product="aminopeptidase N"
                /rbs_spacer="5-10bp"
                /gc_cont="0.404"
                /start_type="ATG"
                /score=476.6
                /codon_start=1
                /locus_tag="10K_08"
```

```
     gene            complement(9715..10371)
                     /rbs_motif="AGGA/GGAG/GAGG"
                     /uscore="0.06"
                     /rscore="4.18"
                     /sscore="8.31"
                     /conf="100.0"
                     /cscore="130.3"
                     /partial=0
                     /tscore="3.07"
                     /rbs_spacer="11-12bp"
                     /gc_cont="0.411"
                     /start_type="ATG"
                     /score=138.6
                     /locus_tag="10K_09"
     CDS             complement(9715..10371)
                     /rbs_motif="AGGA/GGAG/GAGG"
                     /uscore="0.06"
                     /rscore="4.18"
                     /sscore="8.31"
                     /conf="100.0"
                     /cscore="130.3"
                     /partial=0
                     /tscore="3.07"
                     /product="PhoU family transcriptional regulator"
                     /rbs_spacer="11-12bp"
                     /gc_cont="0.411"
                     /start_type="ATG"
                     /score=138.6
                     /codon_start=1
                     /locus_tag="10K_09"
     gene            complement(10396..11154)
                     /rbs_motif="AGGAG"
                     /uscore="0.37"
                     /rscore="11.88"
                     /sscore="16.45"
                     /conf="100.0"
                     /cscore="111.64"
                     /partial=0
                     /tscore="3.07"
                     /rbs_spacer="5-10bp"
                     /gc_cont="0.41"
                     /start_type="ATG"
                     /score=128.1
                     /locus_tag="10K_10"
     CDS             complement(10396..11154)
                     /rbs_motif="AGGAG"
                     /uscore="0.37"
                     /rscore="11.88"
                     /sscore="16.45"
                     /conf="100.0"
                     /cscore="111.64"
                     /partial=0
                     /tscore="3.07"
                     /product="phosphate ABC transporter ATP-binding protein"
                     /rbs_spacer="5-10bp"
                     /gc_cont="0.41"
                     /start_type="ATG"
                     /score=128.1
                     /codon_start=1
                     /locus_tag="10K_10"
     gene            complement(11167..11970)
```

```
                     /rbs_motif="AGGAG"
                     /uscore="-2.14"
                     /rscore="11.88"
                     /sscore="13.97"
                     /conf="100.0"
                     /cscore="132.6"
                     /partial=0
                     /tscore="3.07"
                     /rbs_spacer="5-10bp"
                     /gc_cont="0.398"
                     /start_type="ATG"
                     /score=146.6
                     /locus_tag="10K_11"
     CDS             complement(11167..11970)
                     /rbs_motif="AGGAG"
                     /uscore="-2.14"
                     /rscore="11.88"
                     /sscore="13.97"
                     /conf="100.0"
                     /cscore="132.6"
                     /partial=0
                     /tscore="3.07"
                     /product="phosphate ABC transporter ATP-binding protein"
                     /rbs_spacer="5-10bp"
                     /gc_cont="0.398"
                     /start_type="ATG"
                     /score=146.6
                     /codon_start=1
                     /locus_tag="10K_11"
     gene            complement(11981..12865)
                     /rbs_motif="GGA/GAG/AGG"
                     /uscore="3.77"
                     /rscore="0.91"
                     /sscore="8.97"
                     /conf="100.0"
                     /cscore="105.97"
                     /partial=0
                     /tscore="3.07"
                     /rbs_spacer="5-10bp"
                     /gc_cont="0.423"
                     /start_type="ATG"
                     /score=114.9
                     /locus_tag="10K_12"
     CDS             complement(11981..12865)
                     /rbs_motif="GGA/GAG/AGG"
                     /uscore="3.77"
                     /rscore="0.91"
                     /sscore="8.97"
                     /conf="100.0"
                     /cscore="105.97"
                     /partial=0
                     /tscore="3.07"
                     /product="phosphate ABC transporter permease"
                     /rbs_spacer="5-10bp"
                     /gc_cont="0.423"
                     /start_type="ATG"
                     /score=114.9
                     /codon_start=1
                     /locus_tag="10K_12"
     gene            complement(12855..13769)
                     /rbs_motif="AGGAG"
```

```
                     /uscore="4.24"
                     /rscore="11.88"
                     /sscore="20.43"
                     /conf="100.0"
                     /cscore="123.14"
                     /partial=0
                     /tscore="3.07"
                     /rbs_spacer="5-10bp"
                     /gc_cont="0.414"
                     /start_type="ATG"
                     /score=143.6
                     /locus_tag="10K_13"
     CDS             complement(12855..13769)
                     /rbs_motif="AGGAG"
                     /uscore="4.24"
                     /rscore="11.88"
                     /sscore="20.43"
                     /conf="100.0"
                     /cscore="123.14"
                     /partial=0
                     /tscore="3.07"
                     /product="phosphate ABC transporter permease"
                     /rbs_spacer="5-10bp"
                     /gc_cont="0.414"
                     /start_type="ATG"
                     /score=143.6
                     /codon_start=1
                     /locus_tag="10K_13"
     gene            complement(13772..14647)
                     /rbs_motif="GGAGG"
                     /uscore="0.1"
                     /rscore="14.48"
                     /sscore="17.65"
                     /conf="100.0"
                     /cscore="133.23"
                     /partial=0
                     /tscore="3.07"
                     /rbs_spacer="5-10bp"
                     /gc_cont="0.404"
                     /start_type="ATG"
                     /score=150.9
                     /locus_tag="10K_14"
     CDS             complement(13772..14647)
                     /rbs_motif="GGAGG"
                     /uscore="0.1"
                     /rscore="14.48"
                     /sscore="17.65"
                     /conf="100.0"
                     /cscore="133.23"
                     /partial=0
                     /tscore="3.07"
                     /product="phosphate-binding protein"
                     /rbs_spacer="5-10bp"
                     /gc_cont="0.404"
                     /start_type="ATG"
                     /score=150.9
                     /codon_start=1
                     /locus_tag="10K_14"
     gene            complement(14772..16082)
                     /rbs_motif="GGA/GAG/AGG"
                     /uscore="0.1"
```

```
                     /rscore="0.91"
                     /sscore="4.08"
                     /conf="100.0"
                     /cscore="171.68"
                     /partial=0
                     /tscore="3.07"
                     /rbs_spacer="5-10bp"
                     /gc_cont="0.41"
                     /start_type="ATG"
                     /score=175.8
                     /locus_tag="10K_15"
     CDS             complement(14772..16082)
                     /rbs_motif="GGA/GAG/AGG"
                     /uscore="0.1"
                     /rscore="0.91"
                     /sscore="4.08"
                     /conf="100.0"
                     /cscore="171.68"
                     /partial=0
                     /tscore="3.07"
                     /product="23S rRNA methyltransferase"
                     /rbs_spacer="5-10bp"
                     /gc_cont="0.41"
                     /start_type="ATG"
                     /score=175.8
                     /codon_start=1
                     /locus_tag="10K_15"
     gene            complement(16165..16932)
                     /rbs_motif="AGGA"
                     /uscore="0.77"
                     /rscore="7.92"
                     /sscore="6.26"
                     /conf="100.0"
                     /cscore="90.42"
                     /partial=0
                     /tscore="-3.65"
                     /rbs_spacer="5-10bp"
                     /gc_cont="0.37"
                     /start_type="TTG"
                     /score=96.7
                     /locus_tag="10K_16"
     CDS             complement(16165..16932)
                     /rbs_motif="AGGA"
                     /uscore="0.77"
                     /rscore="7.92"
                     /sscore="6.26"
                     /conf="100.0"
                     /cscore="90.42"
                     /partial=0
                     /tscore="-3.65"
                     /product="inositol monophosphatase"
                     /rbs_spacer="5-10bp"
                     /gc_cont="0.37"
                     /start_type="TTG"
                     /score=96.7
                     /codon_start=1
                     /locus_tag="10K_16"
     gene            complement(16922..17203)
                     /rbs_motif="GGA/GAG/AGG"
                     /uscore="-0.28"
                     /rscore="0.91"
```

```
                     /sscore="4.95"
                     /conf="99.99"
                     /cscore="37.35"
                     /partial=0
                     /tscore="3.07"
                     /rbs_spacer="5-10bp"
                     /gc_cont="0.404"
                     /start_type="ATG"
                     /score=42.3
                     /locus_tag="10K_17"
     CDS             complement(16922..17203)
                     /rbs_motif="GGA/GAG/AGG"
                     /uscore="-0.28"
                     /rscore="0.91"
                     /sscore="4.95"
                     /conf="99.99"
                     /cscore="37.35"
                     /partial=0
                     /tscore="3.07"
                     /product="hypothetical protein"
                     /rbs_spacer="5-10bp"
                     /gc_cont="0.404"
                     /start_type="ATG"
                     /score=42.3
                     /codon_start=1
                     /locus_tag="10K_17"
     gene            complement(17196..17609)
                     /rbs_motif="GGA/GAG/AGG"
                     /uscore="-5.09"
                     /rscore="0.91"
                     /sscore="-7.82"
                     /conf="100.0"
                     /cscore="53.7"
                     /partial=0
                     /tscore="-3.65"
                     /rbs_spacer="5-10bp"
                     /gc_cont="0.353"
                     /start_type="TTG"
                     /score=45.9
                     /locus_tag="10K_18"
     CDS             complement(17196..17609)
                     /rbs_motif="GGA/GAG/AGG"
                     /uscore="-5.09"
                     /rscore="0.91"
                     /sscore="-7.82"
                     /conf="100.0"
                     /cscore="53.7"
                     /partial=0
                     /tscore="-3.65"
                     /product="ArsR family transcriptional regulator"
                     /rbs_spacer="5-10bp"
                     /gc_cont="0.353"
                     /start_type="TTG"
                     /score=45.9
                     /codon_start=1
                     /locus_tag="10K_18"
     gene            complement(17680..18591)
                     /rbs_motif="None"
                     /uscore="0.72"
                     /rscore="-3.27"
                     /sscore="5.08"
```

```
                     /conf="100.0"
                     /cscore="133.22"
                     /partial=0
                     /tscore="3.07"
                     /rbs_spacer="None"
                     /gc_cont="0.404"
                     /start_type="ATG"
                     /score=138.3
                     /locus_tag="10K_19"
     CDS             complement(17680..18591)
                     /rbs_motif="None"
                     /uscore="0.72"
                     /rscore="-3.27"
                     /sscore="5.08"
                     /conf="100.0"
                     /cscore="133.22"
                     /partial=0
                     /tscore="3.07"
                     /product="riboflavin biosynthesis protein RibF"
                     /rbs_spacer="None"
                     /gc_cont="0.404"
                     /start_type="ATG"
                     /score=138.3
                     /codon_start=1
                     /locus_tag="10K_19"
     gene            complement(18588..19421)
                     /rbs_motif="GGA/GAG/AGG"
                     /uscore="4.44"
                     /rscore="0.91"
                     /sscore="7.76"
                     /conf="100.0"
                     /cscore="123.57"
                     /partial=0
                     /tscore="3.07"
                     /rbs_spacer="5-10bp"
                     /gc_cont="0.421"
                     /start_type="ATG"
                     /score=131.3
                     /locus_tag="10K_20"
     CDS             complement(18588..19421)
                     /rbs_motif="GGA/GAG/AGG"
                     /uscore="4.44"
                     /rscore="0.91"
                     /sscore="7.76"
                     /conf="100.0"
                     /cscore="123.57"
                     /partial=0
                     /tscore="3.07"
                     /product="tRNA pseudouridine synthase B"
                     /rbs_spacer="5-10bp"
                     /gc_cont="0.421"
                     /start_type="ATG"
                     /score=131.3
                     /codon_start=1
                     /locus_tag="10K_20"
     gene            complement(19638..19943)
                     /rbs_motif="None"
                     /uscore="-1.81"
                     /rscore="-3.27"
                     /sscore="-9.38"
                     /conf="99.87"
```

```
                     /cscore="38.15"
                     /partial=0
                     /tscore="-3.65"
                     /rbs_spacer="None"
                     /gc_cont="0.399"
                     /start_type="TTG"
                     /score=28.8
                     /locus_tag="10K_21"
     CDS             complement(19638..19943)
                     /rbs_motif="None"
                     /uscore="-1.81"
                     /rscore="-3.27"
                     /sscore="-9.38"
                     /conf="99.87"
                     /cscore="38.15"
                     /partial=0
                     /tscore="-3.65"
                     /product="hypothetical protein, partial"
                     /rbs_spacer="None"
                     /gc_cont="0.399"
                     /start_type="TTG"
                     /score=28.8
                     /codon_start=1
                     /locus_tag="10K_21"
     gene            complement(20600..20884)
                     /rbs_motif="GGAG/GAGG"
                     /uscore="-0.26"
                     /rscore="6.9"
                     /sscore="9.06"
                     /conf="99.99"
                     /cscore="31.03"
                     /partial=0
                     /tscore="3.07"
                     /rbs_spacer="5-10bp"
                     /gc_cont="0.379"
                     /start_type="ATG"
                     /score=40.1
                     /locus_tag="10K_22"
     CDS             complement(20600..20884)
                     /rbs_motif="GGAG/GAGG"
                     /uscore="-0.26"
                     /rscore="6.9"
                     /sscore="9.06"
                     /conf="99.99"
                     /cscore="31.03"
                     /partial=0
                     /tscore="3.07"
                     /product="hypothetical protein, partial"
                     /rbs_spacer="5-10bp"
                     /gc_cont="0.379"
                     /start_type="ATG"
                     /score=40.1
                     /codon_start=1
                     /locus_tag="10K_22"
     gene            complement(21150..21770)
                     /rbs_motif="AGGAG"
                     /uscore="-0.93"
                     /rscore="11.88"
                     /sscore="14.01"
                     /conf="100.0"
                     /cscore="109.89"
```

```
                     /partial=0
                     /tscore="3.07"
                     /rbs_spacer="5-10bp"
                     /gc_cont="0.407"
                     /start_type="ATG"
                     /score=123.9
                     /locus_tag="10K_23"
     CDS             complement(21150..21770)
                     /rbs_motif="AGGAG"
                     /uscore="-0.93"
                     /rscore="11.88"
                     /sscore="14.01"
                     /conf="100.0"
                     /cscore="109.89"
                     /partial=0
                     /tscore="3.07"
                     /product="type I restriction endonuclease subunit S"
                     /rbs_spacer="5-10bp"
                     /gc_cont="0.407"
                     /start_type="ATG"
                     /score=123.9
                     /codon_start=1
                     /locus_tag="10K_23"
     gene            complement(21893..22387)
                     /rbs_motif="GGAGG"
                     /uscore="6.28"
                     /rscore="14.48"
                     /sscore="23.83"
                     /conf="100.0"
                     /cscore="87.16"
                     /partial=0
                     /tscore="3.07"
                     /rbs_spacer="5-10bp"
                     /gc_cont="0.378"
                     /start_type="ATG"
                     /score=111.0
                     /locus_tag="10K_24"
     CDS             complement(21893..22387)
                     /rbs_motif="GGAGG"
                     /uscore="6.28"
                     /rscore="14.48"
                     /sscore="23.83"
                     /conf="100.0"
                     /cscore="87.16"
                     /partial=0
                     /tscore="3.07"
                     /product="lipid hydroperoxide peroxidase"
                     /rbs_spacer="5-10bp"
                     /gc_cont="0.378"
                     /start_type="ATG"
                     /score=111.0
                     /codon_start=1
                     /locus_tag="10K_24"
     gene            complement(22452..22820)
                     /rbs_motif="None"
                     /uscore="0.35"
                     /rscore="-3.27"
                     /sscore="-5.77"
                     /conf="100.0"
                     /cscore="50.42"
                     /partial=0
```

```
                /tscore="-3.65"
                /rbs_spacer="None"
                /gc_cont="0.322"
                /start_type="TTG"
                /score=44.6
                /locus_tag="10K_25"
CDS             complement(22452..22820)
                /rbs_motif="None"
                /uscore="0.35"
                /rscore="-3.27"
                /sscore="-5.77"
                /conf="100.0"
                /cscore="50.42"
                /partial=0
                /tscore="-3.65"
                /product="hypothetical protein, partial"
                /rbs_spacer="None"
                /gc_cont="0.322"
                /start_type="TTG"
                /score=44.6
                /codon_start=1
                /locus_tag="10K_25"
gene            complement(22866..23231)
                /rbs_motif="GGA/GAG/AGG"
                /uscore="-0.47"
                /rscore="0.91"
                /sscore="-3.2"
                /conf="96.63"
                /cscore="17.81"
                /partial=0
                /tscore="-3.65"
                /rbs_spacer="5-10bp"
                /gc_cont="0.325"
                /start_type="TTG"
                /score=14.6
                /locus_tag="10K_26"
CDS             complement(22866..23231)
                /rbs_motif="GGA/GAG/AGG"
                /uscore="-0.47"
                /rscore="0.91"
                /sscore="-3.2"
                /conf="96.63"
                /cscore="17.81"
                /partial=0
                /tscore="-3.65"
                /product="haloacid dehalogenase, partial"
                /rbs_spacer="5-10bp"
                /gc_cont="0.325"
                /start_type="TTG"
                /score=14.6
                /codon_start=1
                /locus_tag="10K_26"
gene            23361..24617
                /rbs_motif="AGGA"
                /uscore="3.29"
                /rscore="7.92"
                /sscore="14.27"
                /conf="100.0"
                /cscore="100.67"
                /partial=0
                /tscore="3.07"
```

```
                     /rbs_spacer="5-10bp"
                     /gc_cont="0.396"
                     /start_type="ATG"
                     /score=114.9
                     /locus_tag="10K_27"
     CDS             23361..24617
                     /rbs_motif="AGGA"
                     /uscore="3.29"
                     /rscore="7.92"
                     /sscore="14.27"
                     /conf="100.0"
                     /cscore="100.67"
                     /partial=0
                     /tscore="3.07"
                     /product="transposase"
                     /rbs_spacer="5-10bp"
                     /gc_cont="0.396"
                     /start_type="ATG"
                     /score=114.9
                     /codon_start=1
                     /locus_tag="10K_27"
     gene            complement(24687..25967)
                     /rbs_motif="GGAGG"
                     /uscore="0.39"
                     /rscore="14.48"
                     /sscore="17.93"
                     /conf="99.99"
                     /cscore="227.32"
                     /partial=0
                     /tscore="3.07"
                     /rbs_spacer="5-10bp"
                     /gc_cont="0.406"
                     /start_type="ATG"
                     /score=245.2
                     /locus_tag="10K_28"
     CDS             complement(24687..25967)
                     /rbs_motif="GGAGG"
                     /uscore="0.39"
                     /rscore="14.48"
                     /sscore="17.93"
                     /conf="99.99"
                     /cscore="227.32"
                     /partial=0
                     /tscore="3.07"
                     /product="O-acetylhomoserine sulfhydrylase"
                     /rbs_spacer="5-10bp"
                     /gc_cont="0.406"
                     /start_type="ATG"
                     /score=245.2
                     /codon_start=1
                     /locus_tag="10K_28"
     gene            complement(26088..28400)
                     /rbs_motif="GGAG/GAGG"
                     /uscore="3.96"
                     /rscore="6.9"
                     /sscore="13.93"
                     /conf="99.99"
                     /cscore="429.0"
                     /partial=0
                     /tscore="3.07"
                     /rbs_spacer="5-10bp"
```

```
                     /gc_cont="0.425"
                     /start_type="ATG"
                     /score=442.9
                     /locus_tag="10K_29"
     CDS             complement(26088..28400)
                     /rbs_motif="GGAG/GAGG"
                     /uscore="3.96"
                     /rscore="6.9"
                     /sscore="13.93"
                     /conf="99.99"
                     /cscore="429.0"
                     /partial=0
                     /tscore="3.07"
                     /product="ATP-dependent DNA helicase PcrA"
                     /rbs_spacer="5-10bp"
                     /gc_cont="0.425"
                     /start_type="ATG"
                     /score=442.9
                     /codon_start=1
                     /locus_tag="10K_29"
     gene            complement(28570..28818)
                     /rbs_motif="None"
                     /uscore="-2.21"
                     /rscore="-3.32"
                     /sscore="-3.17"
                     /conf="52.15"
                     /cscore="3.54"
                     /partial=0
                     /tscore="3.02"
                     /rbs_spacer="None"
                     /gc_cont="0.386"
                     /start_type="ATG"
                     /score=0.4
                     /locus_tag="10K_30"
     CDS             complement(28570..28818)
                     /rbs_motif="None"
                     /uscore="-2.21"
                     /rscore="-3.32"
                     /sscore="-3.17"
                     /conf="52.15"
                     /cscore="3.54"
                     /partial=0
                     /tscore="3.02"
                     /product="hypothetical protein"
                     /rbs_spacer="None"
                     /gc_cont="0.386"
                     /start_type="ATG"
                     /score=0.4
                     /codon_start=1
                     /locus_tag="10K_30"
     gene            28916..30259
                     /rbs_motif="AGGAG/GGAGG"
                     /uscore="-0.15"
                     /rscore="7.19"
                     /sscore="10.11"
                     /conf="99.99"
                     /cscore="224.67"
                     /partial=0
                     /tscore="3.07"
                     /rbs_spacer="11-12bp"
                     /gc_cont="0.412"
```

```
                     /start_type="ATG"
                     /score=234.8
                     /locus_tag="10K_31"
     CDS             28916..30259
                     /rbs_motif="AGGAG/GGAGG"
                     /uscore="-0.15"
                     /rscore="7.19"
                     /sscore="10.11"
                     /conf="99.99"
                     /cscore="224.67"
                     /partial=0
                     /tscore="3.07"
                     /product="sodium:alanine symporter"
                     /rbs_spacer="11-12bp"
                     /gc_cont="0.412"
                     /start_type="ATG"
                     /score=234.8
                     /codon_start=1
                     /locus_tag="10K_31"
     gene            30388..31758
                     /rbs_motif="AGGA"
                     /uscore="5.45"
                     /rscore="7.92"
                     /sscore="16.43"
                     /conf="99.99"
                     /cscore="265.64"
                     /partial=0
                     /tscore="3.07"
                     /rbs_spacer="5-10bp"
                     /gc_cont="0.416"
                     /start_type="ATG"
                     /score=282.1
                     /locus_tag="10K_32"
     CDS             30388..31758
                     /rbs_motif="AGGA"
                     /uscore="5.45"
                     /rscore="7.92"
                     /sscore="16.43"
                     /conf="99.99"
                     /cscore="265.64"
                     /partial=0
                     /tscore="3.07"
                     /product="tRNA modification GTPase"
                     /rbs_spacer="5-10bp"
                     /gc_cont="0.416"
                     /start_type="ATG"
                     /score=282.1
                     /codon_start=1
                     /locus_tag="10K_32"
     gene            31874..32002
                     /rbs_motif="None"
                     /uscore="1.05"
                     /rscore="-6.48"
                     /sscore="-3.88"
                     /conf="65.22"
                     /cscore="6.62"
                     /partial=0
                     /tscore="1.55"
                     /rbs_spacer="None"
                     /gc_cont="0.287"
                     /start_type="ATG"
```

```
                     /score=2.7
                     /locus_tag="10K_33"
     CDS             31874..32002
                     /rbs_motif="None"
                     /uscore="1.05"
                     /rscore="-6.48"
                     /sscore="-3.88"
                     /conf="65.22"
                     /cscore="6.62"
                     /partial=0
                     /tscore="1.55"
                     /product="AraC family transcriptional regulator"
                     /rbs_spacer="None"
                     /gc_cont="0.287"
                     /start_type="ATG"
                     /score=2.7
                     /codon_start=1
                     /locus_tag="10K_33"
     gene            32250..32840
                     /rbs_motif="AGGA"
                     /uscore="3.88"
                     /rscore="7.92"
                     /sscore="7.67"
                     /conf="100.0"
                     /cscore="54.35"
                     /partial=0
                     /tscore="-3.48"
                     /rbs_spacer="5-10bp"
                     /gc_cont="0.357"
                     /start_type="GTG"
                     /score=62.0
                     /locus_tag="10K_34"
     CDS             32250..32840
                     /rbs_motif="AGGA"
                     /uscore="3.88"
                     /rscore="7.92"
                     /sscore="7.67"
                     /conf="100.0"
                     /cscore="54.35"
                     /partial=0
                     /tscore="-3.48"
                     /product="AraC family transcriptional regulator"
                     /rbs_spacer="5-10bp"
                     /gc_cont="0.357"
                     /start_type="GTG"
                     /score=62.0
                     /codon_start=1
                     /locus_tag="10K_34"
     gene            complement(33050..33337)
                     /rbs_motif="None"
                     /uscore="-2.26"
                     /rscore="-3.27"
                     /sscore="-2.46"
                     /conf="99.98"
                     /cscore="39.71"
                     /partial=0
                     /tscore="3.07"
                     /rbs_spacer="None"
                     /gc_cont="0.372"
                     /start_type="ATG"
                     /score=37.3
```

```
                     /locus_tag="10K_35"
     CDS             complement(33050..33337)
                     /rbs_motif="None"
                     /uscore="-2.26"
                     /rscore="-3.27"
                     /sscore="-2.46"
                     /conf="99.98"
                     /cscore="39.71"
                     /partial=0
                     /tscore="3.07"
                     /product="hypothetical protein"
                     /rbs_spacer="None"
                     /gc_cont="0.372"
                     /start_type="ATG"
                     /score=37.3
                     /codon_start=1
                     /locus_tag="10K_35"
BASE COUNT    10179 a   6638 c   6336 g  10338 t
ORIGIN
        1 tttcagaaaa aacgaaacca gtggtatcta aagtagtaac aacacctaac ggtaaagtct
       61 tcttcgttta aacttcaaac gaaattctta aggaaactga aaaagttact gaaactccaa
      121 gccaaagtca cgacgttgta aaacgacggc cagtgaattg taatacgact cactataggg
      181 cgaattcgag ctcggtaccc ggggatccca ccggagttcc accagataac ttctcaccaa
      241 caggacaact ttgggggaat ccaatttatg attgggataa acacaaagca gaaaactatg
      301 cttggtgggt cttccgtatt caagaatcat tcaaactttt tgattgcttg cgtatcgacc
      361 acttcaaagg tttctctgat ttctgggaaa ttcctgctgg agatgaaact gctgaaaatg
      421 gtaaatgggt tccaggacct ggttatgacc tcttcaaagt ggttaaagaa gagttgggtg
      481 atcttaagat tattgccgaa gaccttggta atattgatga taaaacacgt aaacttcttg
      541 cagactgtgg ttacccagga atgaagattc ttcactttgg attctatgat accactggta
      601 acagcattta tgttccacac gcatacacac aacactcagt ggcttacact ggtacacatg
      661 ataatgaagt tactaatggt tggtataata acttgacaca agagcaacgt gattacactg
      721 atgcttatat caaccgtcgt caaggtgaac caatcacacg tgcccttctt cgcacactct
      781 tcgcaacagt aagtaacaca gctattgcga ctatgcaaga tattcttgat aaaaatgaaa
      841 atagccgtaa aaacatccct aacacagttg gaggaaactg gagatggcgt atgcttccag
      901 gtgaaatcac cgtcgataag aaagaatttc tgactgatat tacagaaaga tataatcgag
      961 gaaatcgagg aaataaataa aatgtctaca tttacaaatt acgttgaagc gaaaaataaa
     1021 aaccttaaat aactttcaaa cgaagaaatc tactaccttt tgttagaatt tgtaaaagaa
     1081 gcagctgctt caaaacctaa aaatgatagt aaacgtaaag tttactacat ctcagctgag
     1141 ttcttgattg gtaaactttt gtctaataac ttgattaact tgggtatcta taaagatgtg
     1201 aaagctgaat tagctgctgc cggaaaatca atcagtgaaa tcgaagatat tgaaccagaa
     1261 ccatctcttg gtaacggtgg tttgggacgc ttggctgcat gtttcataga ttctatgtca
     1321 acacttggca ttaacggtga aggtgtcggt cttaaatacc actgtggtct tttcaaacaa
     1381 gtcttcaaag ataacaaaca atatgctgaa ccagattact ggattgaaga tcaatcatgg
     1441 ttggttccaa ctgacatctc ttacgatgtc ccattcaaaa acttcacttt gaaatctcgt
     1501 ttggaccgtt tggatgtctt gggttacaag aaagaaacta agaactacct taacctcttc
     1561 gatatcaatg gtttggacta tggtttaatt gaaaaaggaa tcacttttga tcaagatgaa
     1621 attcaaaaga acttgacact cttcctttac ccagatgatt caactcgtaa aggtgagctc
     1681 ttgcgtatct accaacaata cttcatggtg tcaaacgctg ctcaactttt gattgacgaa
     1741 gctatcgaac gtggttctaa ccttcacgat cttccagatt acgctttcgt acaaatcaac
     1801 gatactcacc catcaatggt tatccctgaa ttgattcgtc ttttgacaga aaaacatgga
     1861 tttgaatttg atgaagcagt agcaatcgtg agcaaaatgg taggttacac aaaccacact
     1921 atcttggctg aagctcttga aaaatggcct cttgactatc ttgaagaagt tgttccacac
     1981 cttgttgtta tcatcaagaa attggatgcc atcgttcgtg gcaaattcga tgatccacgt
     2041 gttcaaatta ttgacgaaca caaccgtgtt cacatggccc gcatggatat ccacttctct
     2101 acaagcgtaa acggtgttgc aaagcttcac acagaaatcc ttaagacatc agagttgaaa
     2161 cctttctacg acctttaccc agaacgtttc aacaacaaaa caaacggtat ttcattccgt
     2221 cgttggttgg aattcgctaa ccaagaactt gctgattaca taaaagagtt aattggtgat
     2281 gattacctta ctgatgccac tcaattggag aaattagctg cctttgcaga tgatcctgca
     2341 gtacacaaac gtttgtctca aatcaaatat gataacaaat tagctcttaa acgttatctt
     2401 aaatccaata aaggtattga tcttgacgaa aattcaatca tcgatacaca aatcaaacgt
     2461 ttccacgaat acaaacgtca acaaatgaac gctttgtatg tcatcacaaa tacttggaaa
```

2521 tcaaaaaaag gaaaccttcc aaaacgtaaa atcacaatca tctttggtgg taaagcagct
2581 cctgcctatg ttattgctca agacatcatt cacttgatct tatcactttc tgaactcatt
2641 aataatgatc caaaagtaag caaatacctc aacgtccact tggttgagaa ctacaacgta
2701 acagttgctg aacaccttat cccagcgaca gatatttcag aacaaatctc attggcttct
2761 aaagaagctt caggtacagg taacatgaaa ttaatgctta atggtgccct tacacttggt
2821 acaatggatg gtgccaacgt tgagattgct aaattggtag gaccagaaaa catcttcata
2881 ttcggtaaag actctgatac aatcattgac ctttataaaa aagaaggtta cgtatcacgt
2941 gattactacg aaaatgatgc taacatcaaa gcagctgttg attttatcgt aagcgaggac
3001 cttgtaaaag ttggggataa agaacgtttg gaacgtcttt acaaagaact tatctctaaa
3061 gactggttca tgacattgat tgacttggcc gaatacatcg agaagaaaga agaagtatac
3121 gcagcttacg aagatcaaga tgcatggaac aagaaagttg tttacaacat tgctaaagca
3181 ggattcttct catctgaccg tacaatcgaa caatatgaca aagatatctg gcacacaaaa
3241 tattagccta gttgcataga aggaaaacaa gatcagaaat ggtcttgttt ttttgcatga
3301 aaaaacacag ccaataggat aattaaccgt gtttacataa ataagattat gttatttcgc
3361 atgataattt agtatatcat taatatatgc ttctttattt gatgaatcgt aagtattaga
3421 cttagagtat tttagataat ggatttcgtc ttttgttgtt ctagattctt cttctaggta
3481 agaatccgta atagtaatct taaaattctc gctcttacct gttaaatcaa gactaagtgg
3541 aaaatatgta aattcatctg agtaaaagac catcttataa aaattatagc catctcctct
3601 aaaatcattt aagcgaggaa atgttgttat attgtatctt tcatcccaaa gattaccata
3661 ttctctaata tcgtagtcat cgaagaagtc ctgaattctt tctttagttc tttctttact
3721 agcatcatcc ttatcaaagg taacaaaggg aagcctccca atcccatatt gaaatgtctc
3781 cttcatttcc atagataata taacttttca ttttttctga cttaggattt ttcaactgaa
3841 agttgtattc acttacacca tcttttacag ctccagtttg atataggggta tcgccaggga
3901 ctatcaaaaa gagaaaatct cttaggtaca attcgaagtc ggtatctatt gttttatcat
3961 gtaatccagg gaaacgctga atcatttgtt tgtaattctc tgcattagag aaccattctt
4021 tatcaaagcc tagaccgact tcaaaaactt tataatcgaa attgttttca taacgttcag
4081 aatagttttc agtggaagtc acctgtctaa atggtaaagt catattggta ttatctgtaa
4141 aagtgatatg caaaaaaagc cttctcggat caattccttt gtcagtataa tggctgtcct
4201 tatatgtaga gagattatca gggaaatcgt cttttacaaa gtgtaaaact tcaatctcta
4261 tatctttgac aatttctttt tgaggtttta tcttcttgac cttgtaatga taagccttgt
4321 aatagtaatt gctcttatca tagagataag gtctaaaagc tctattataa ccgttaaaaa
4381 gaagaagacc tatacaagcc ataagaagaa gaagcgctag tatgattttc agtcttttac
4441 aatacctaca aaattacttt atcatcccta actcccatgt tttttactat tatatcatga
4501 aaataattgc tctcaagcca tcttcgtgta ctttgtcacc gttttccatt ataatatagg
4561 taccacatta caagaggaga tatcatgaaa tatatcgtaa acacatcaaa tgatcctgcc
4621 tataatattg ctttggaagc ctatgccttt cgtgaattgg tagatgaaga tgaattgttc
4681 attctctgga ttaaccgtcc agctatcatt gttggtaaac accaaaatac cattcaagag
4741 attaacaaag aatatactga tgctcatgat attaaggtcg ttcgtcgttt atctggtggt
4801 ggtgctgttt atcacgattt gaacaacctc aactatacta tcatctcaaa caaggcagat
4861 gaaggcgcat ttgactttaa gacattttct caaccggtaa tcgcaacact tgctgatttg
4921 ggcgttaagg ctgaatttac tggccgtaac gatcttgaga tcgatggtaa aaaattttgc
4981 ggaaatgccc aagcctacta taaaggtcgt atgatgcacc atggttgctt actctttgac
5041 gttgatatgt ccgttcttgg tcaagccctt aaagtcagca aagacaaaat tgaatctaaa
5101 ggcgttaagt ctgtacgtgc tcgtgtgact aacattgtag acgaattgcc agaaaaatgc
5161 actgtaaatg aattctcaga taagatcttg gaaaaaatga aagaattcta cccagaaatg
5221 accgaatatg tcctttctga agacgatttg gctaagattc aagagtctta tgagaaacaa
5281 ttcaatacat gggactggac atatggccaa tctccagaat acacggtaga acgtaatgtc
5341 cgttacccag ctggtaagat ttctacctat gcaaatgttg aaaattcaat tattaaatct
5401 gttaagattt acggagattt ctttggtatt ggcgatgtat ctgatattga agaacttttg
5461 gtcggtgtac cttacgagta cgaatatgtc ctggcaaaac tcaaaaccat tgacacaacg
5521 ctttatttct cacgcatgac aactgaggag gttgcaaaag ctatcgtagc ttaatcattg
5581 ttatcattgt ttgaaacaat ctggtaaact ccaggttgtt tttgtatgcg taaattattg
5641 tagttgatta gtaagttttt aatagcatag gacagcaaac ctaaaaagcc cataagagaa
5701 gaacacctca taacatcttt ccgaaaaact gtaattttct tgaaagaaat ataagggtgt
5761 gatatacttc tattaagtaa tttaagtaat tatggagaat aagattaatg aaacgtgaac
5821 tcttactaga aaaaattgaa gaatataaat ctcttatgcc ttggtttgtt ttggagtatt
5881 atcaatctaa actatcggta ccgtattctt tcacgacctt atatgaatat ctcaaggaat
5941 ataaacgctt ttttaactgg ttaattgact caggtatttc agatgctgat gatattgcct
6001 caattcatat caaaaccttg gagaatctaa ctaaaaaaga tatggaatcg tttgtcctct
6061 atctacgtga acgtccatct ttaaatacct attcaaagaa acagggtgtc tctcaaacaa
6121 ccattaatcg tacgctttca gctctatcta gtctctataa gtatttaact gaggaggtcg

6181 agggacctga tggtgaacca tatttctatc gtaacgtcat gaaaaaaatt tcgactaaga
6241 aaaagaaaga gaccttggct gcacgtgctg agaatatcaa acaaaaactt tttctaggcg
6301 atgaaaccat gaagttcctt gattatgtag aaaatgaata cgaagtcaaa ctctcaaatc
6361 gtgcgaaatc ttcgttttat aagaataaag agcgagattt agccatcatt gccctgctgc
6421 tgtcttcagg cgttcgactc tctgaggctg taaatctgga ccttaaagat atcaatttaa
6481 aaatgatggt tattgacgtt actcgaaaag gtggtcaacg ggactcggtg aatatggcaa
6541 gttttgcaag accctatctt gaaaactatc ttagcatacg taataaacgc tataaggctg
6601 aaaagcaaga tgttgctcta tttttaacag aatatcgagg cgttcccaac cgtattgatg
6661 cttcaagtat cgaaaaaatg gttgggaagt attctcagga tttcaagatt cgtgtcactc
6721 cccacaaact acgtcatact ttggcaacac gtctttatga tgctactaag tctcaagttt
6781 tagttagtca tcaacttggt catgcttcca ctcaggtcac tgatctttac acccatattg
6841 taaatgatga gcaaaaaaaa tgctctagat aatttataat taaccaatca aaatcattcc
6901 gatttaggtt gattattttt attatacata aatttaatta tgtgatgcta attttattta
6961 cgattgttag cagaactaaa attatgtaaa tcattgtgaa taaaaacaaa ctctccttca
7021 tggaaagctt gtttttttata ttaataatct ttaagagctg attcaaccgc agctttttct
7081 ttttcaatta agtctacgcg tgcagcaatt gttttaattc ccatcaaaat attacgttcc
7141 aaagctttgt cacttgtttg tggctcgaaa aatgcaatat attggtcaag acgttcttga
7201 tttttaaaga ttcctgctgg gatattaaca aaactatcaa agctcatatc gccaccaaga
7261 gctgctttaa tccattccca gtttctttta gcccagtccc aaacggtctc ttgagcaaaa
7321 gatttgctga ggaagttcat ataccagaga tacaaatctt gcggtttaac aacgttttta
7381 tcttttagct gacctagtac gtagtcaaga ccttcttgat ttttaagata tgaaagtgct
7441 tgggtcaatt gacgacggaa gttactgtcg tttgttgcca cgtatgcatt aatatactct
7501 tcaacaagag acagactgtt ttcttgtttc atttggttga tgaggacaag gccacggata
7561 cttgctggaa tgctttctat gttttcttta tgggcttgga aaacatttgc ggctgcaagg
7621 actgttggtt gatagtctgc ttcaatcaag tagctgagag ctgtttgacg gaccatttca
7681 tcttcatcag actccccttc tttggcatcg aaaccaaggc gttcaaagtc attttggaat
7741 tgacgacgta ccaaggcttt ataatgcact tcggcttctg tatcttcatc gataaagcgt
7801 ttcaaaccag caaggatttg agatttagct tgagaaatca agaagctttc ttctttttcg
7861 acaagatcaa gaagaccaac taggcttgca tacgagatac gtccactttc cgctagaaga
7921 cggcgttctt gtaagatttg aagtttactt acagtatcaa ggtttgcaaa atcttctaag
7981 atattgtcca agagttgtcc ttgatagtct gtgatgtaat gtgctgtatt agctgtatta
8041 agacggagaa ctccattatt ttcagtagca agctggctat aatttggaat ctcaatacgc
8101 tcttctgaaa gtgtatctgg aagcccattc cagtttgtat tcaatggaat ctcccaaagg
8161 cgccctttgt cttcatgttc accaatgaag aattgttttt gactaaggat aagcgtatca
8221 tcaacaactt ctgcactaac cactgggtat cctggttgct ctaaccatgt gtccatgaag
8281 ctagagacat ccttacctga agcctctgaa agagcattcc aaaggtcacg tccaacagta
8341 ttgttatatt ggtgtttttc aaagtaagct ttaagccctt tggcaaaggc ttcatcgcct
8401 aaccaacggc ggagcatatg cataagacgg ctacccttgg cataaacaat agcactatca
8461 aagagggtgt taatctcgtc tggatgactg acttccatgt ggacagactg tacgccgtct
8521 gttgcgtcac gttgaagagc attaggtaca ccagttgttt ggaaatcttc aaagatgttc
8581 cagcttggtt caatagcatt cactgaaaca tactccatca tgttggcgaa actttcgtta
8641 agccataaat catcccacca tttcatggta acaaggttac cgaaccattg gtgagccaat
8701 tcgtgagcaa caacaagggc aacttgttga cggcttgcag ctgagctatt ttcatctaca
8761 aggaggtaaa cttcacgata ggtaacaaga ccccagtttt ccatggcacc cgctgaaaag
8821 tcaggaagag caaggtggta tgataatgga ataggatact tgacttggaa gtagtcttca
8881 tagaattcga tgacacgaac ggcaatatca agagcgaagt cgaagctatt ttctgcttgt
8941 gcaactgtag cgaagacacc aacttcagtg ccgtttttttg ttttagctgt tttaccatga
9001 agagcaccga aaccaaaggc gagaaggtat gtagacatgc gaggtgttgt ctcgaatgtc
9061 caaactccag tttcttcacg caaatggcta ttgatttctg gcatgtttga caaggcagta
9121 tcgccctctt cagcgtcaaa tttcaatgaa agatcaaaag ttgcttttgc ttctggttca
9181 tcaacacaag gaaaagcttc acgagcgaaa tgagattcaa actgtgttga aataatttcc
9241 ttcttttcac cattgtaagt gtaatatgat ggataaatac cagtcatatt atctgtgatg
9301 cggccagaaa attctatgaa aattgttaga accccagttt ctggaagttc aatatgaaaa
9361 gcctcattgg catcatccat ttggaagttc aaggattcat tatccaagag aactgaatta
9421 atggtaaggt ctttttggtg aagagagatg tgattatcaa tggcttcacc agtaatggca
9481 acattccctg taaaggtctt ttcagaacga ttaatgtcca agaaaagatt gtaattttca
9541 gggatgaaac tttcgataaa acgagcaaca gatgctgtca tatgtaaata ctcctaatct
9601 aactttttcgt atcgaaacag actgatattt gtttcgatat caggtacttt aacaaaagaa
9661 atgagcctga attattcaat acaggctcat tataacatag attttaaact ttgcctataa
9721 ttcaatgatt tttccagact tgaggtagac aacccattca caaagattac gggcataatc
9781 accaatacgt tctaggtaca cgagtacttg gatatactct ttggcagcaa atgctgcatc

9841 tggattttcg cggacacctt ctactgtcat agcttggatg tcacggaaat aattatcaat
9901 aatctcgtct gaagctgcga tttcataagc acgttcttcg tcaccattca agtaaacttc
9961 gagagcttct tcgaccatgt ggcgggcggc ttttcccatt ttcttgattt cagtttcaat
10021 atctgtcatt ctgctttcac ccttaatacg gatggttgct ttagcgatgg ccaccgcatg
10081 gtcacccata cgttcgacgt cactagatgc ttttaatgca gtgattacag tacgtaaatc
10141 acttgataca ggttgctgga gcgcaataag ttcaagtgat tttttctcaa gtttggtttc
10201 gtagttattg acgatttcgt cgttttcgat aacttccttg gctaattcaa ggtcatgact
10261 aataaaggca cgcagagatt ttttgagctg tgcagcaact tcagtaccca tggcataaaa
10321 ttggttgtga agcttttcta attcttcttc aaagggtgtt cgtaacatca taattgttaa
10381 tgactccgtc ctttcttaac ctaacttacc ggtaatgtaa tcttcggttt ctttgtgttg
10441 tggattaagg aacatgtctt tagttgatga atactcaata agttctccat cgaggaaaaa
10501 ggctgttcgt tgagatatac ggcttgcctg ttgcatagaa cgtgtgacaa gcaacaatgt
10561 atattgatct ttaaggttat agagtgtttc ttcaatctta ccagcggaaa tagggtctag
10621 ggcagatgtt ggttcgtcaa gaagaatcac ctttggtgag gtggcaagta cccgtgcaac
10681 acaaactcgt tgttgttgcc cacctgaaag accaagtgct gagtcatgaa gtcggtcctt
10741 aacctcctcc cagactgaag ctcctttag agattcttct actgctttgt caagaaccgc
10801 cttatctttg ataccattga tacgaaggcc atagactacg ttctcatata tagacatagg
10861 gaagggattg ggttgttgga agaccatacc aatttcctta cggagttcaa cagtatctgt
10921 tcgaggactg tagacattat ggccattgta ggaaactgca ccagtaactg tgcattcagg
10981 gtttaagtcc cccattctgt tgatagaacg aagcaaggtt gattttcctg atccagatgg
11041 cccgataagg gctgtgattt catttggata gaaatccata gtcacattat taatggcttt
11101 tttcttattg aaatacaccg ataaatcatt gattgaaatg attggttcag tcataagtgc
11161 tcctttctat ccaaagcgtc ctgatacata gtcactggtt gactgtagag cagcatcttg
11221 gaagattttc ttggtttcat catattcgat aagatcacca aggtagaaaa atgctgtata
11281 atcactggca cgagccgctt gttgcatatt gtgtgtgacg ataataatgg aataatcttc
11341 tttcagctca aacatggttt cttctagctg catagtagct acaggatcta atgaagctgc
11401 tggctcatcc ataagaagaa tctgtggttt aacggcgata gcccttgcga tacaaagacg
11461 ttgttgctga ccaccagaga gtgtaaaagc tgacttatga aggtcatctt ttacttgttc
11521 ccaaagtccc gcttgtttaa gtgatgtttc aacaatctca tccaaagttt gcttatcttt
11581 cacaccatta cattcatggg caaaggtgat gtttttatag atagacttgg caaatgggtt
11641 tggacgttga aagaccattc caatgtgttt gcgcatttca taaacattga tattacttgc
11701 attgacatca actccttcat aaaggatttg tccagtaacc ttggcaacat caatagtatc
11761 attcatgcgg tttaggctac gaagaaaggt tgatttccca catcctgagg gaccgatgag
11821 tgcagtgatt ttattttttt cgaattgcat atcaatacca ttgatggctt ctttttgacc
11881 atagtagaca tgcaaatcct tggtttcaag agcgagcttc ttctctggga aggtgataat
11941 atgacgctcg tcccaattgt attttgtcat gaaatctcct ttaagcggat gttaatttct
12001 tatggaggta agctcctaaa cgacgtgcac caaagttgaa aatcaagatg aaaatcataa
12061 gcactgctgc tgaaccagca gacactgcag tggcgtcagg tgtcgtacct tctgagttga
12121 ccttccagat gtgaacagca agtgtctcag cttgacggaa aattgatatt ggactagata
12181 tgctaagcgg attccagttg ctccagttaa gcgctggtgc agattgtcct gctgtgtaaa
12241 taagtgctgc tgcttcccca aaaatacgtc cagaggaaag tactattcca gtcacaatac
12301 caggcaaagc ttcaggaatg acaacgtgac atatagtttc ccatctagac aagcccaagg
12361 caagtccagc ttcgcgttga gtgtggtgaa ctgtgcgtaa actatcttca atggtacgag
12421 tcatttgagg caagttaaag acagtcaatg ccaaggcacc tgaaaggatt gaaaatccgt
12481 actggaattg tacaacaaaa attaaataac caaagagtcc gaccaccact gaaggcagtg
12541 atgaaaggat ttcaatacag gtacggacaa agttgctgag ccggcctttt ttggcatatt
12601 ctgataggaa gataccagct cccattgaaa gaggaacaga aataattaaa gtaataacca
12661 acaaaaataa tgaattgtat agttgaactc caattcctcc tccagctttg tgagatgatg
12721 acatactagt caggaatgac caactgatgt gtggaatccc gcgaaccaag atatagagaa
12781 tcaaggctgc taggattgtt acaataatgc cagcaatcac atagagcgtg ctgatagcaa
12841 ttttatcgac ttttttagcg ttcataattt ctctttctct cacgagtgat aaatttcacc
12901 aacatattga aaccaagact catcaataag agcaccaagg caagtgacca aaggacatta
12961 tttgaaacag ttcccatgac tgtatttccg attcccatag tcaatacaga ggtcaacgtt
13021 gctgctggtg tcgttaaaga cgtaggaata actacggaat ttccaacaac catctgaacg
13081 gcaagagcct caccaaaggc acgggccata ccaaagataa cggcagtgaa aatacccggt
13141 ttagccgcat taagtgtcac acgccaaatt gtttgccagc gagtagctcc catggcaaga
13201 ctagcttcac gataatgacg tggcactgca cgaagtgcat ctgttgtcat aaaggttacg
13261 gttggtagaa tcatgacgaa taagacaaag gtacctgaaa ggataccaaa acctgttcca
13321 ccaaaaaatga agcgaacaaa tggaaccacg acttgcagac caatgaatcc gtaaacaaca
13381 gatggaattc ctactaagag ttcaatagcg ggttgtagga atttagctcc cttaggagaa
13441 atctcagtca taaagatagc agcaccaatg gcaaatggcg ttgcaatcaa ggctgaaagt

13501 aaggtaacca aaaatgaccc tataatcatt ggcagagccc cgaatttgcc ttcagacggt
13561 gaccaatcgc cactaaagag aaactcgact aagctgactt tattaacaaa gaaagtcgcc
13621 aagccttttt gagcaacgaa aagtaaaatc aatgctacga tgaaaacaat caaggccaag
13681 caaaggaaag tgattgtttt accaaatttc tcaaggcgtg aatttttaga gggtgaagtt
13741 aattgtttca tcaatttttc atttttcatg attactcctc actcttcttt gtaaccttgc
13801 cgtcagcatc cttagtaact ttcatagtat gaattggaat gtatcccatt tttttaacga
13861 tattttcttg aacctcgtca ctaatcatgt agtctaagaa ttgtttggtg tagctattag
13921 gtttcccttt agtatacata tgctcgtagg accaaatagg ccaatcgtta gtcgcaacat
13981 ttttcgcatt aggttcaaat ccatttagtt tcaatgtttt aactgaatca tcaaggtaag
14041 caaatgatag gtatgaaata gcacctggtg tctgagcgac aattgactta accattccgt
14101 tagaatcttg ctcctgggta cggattggtg atttgccatc cataatcaca gcatcgaaag
14161 ttacgcgtgt tcctgagcta gctgctcggt taacgatagt aatttccaaa tcctgtccac
14221 caagctgttt ccagtttgtg atttttccag tgaagatttt acgaagttgt tctgttgtca
14281 aattatcaac ggatactttc tgattagtga taactgcaat acctgcaact gcaacttgaa
14341 agtctacgag cttactggca tcgattccag atttttcctc agcgaaaaga tcactattcc
14401 caatttcaac agcacctgat tgcacttggg aaagtccagt accagaaccg ccccccttgga
14461 cgttaaccac aatttcagga ttcttttcag caaaaccata tgatgctgct tctactaagg
14521 gttgaagagc cgtagaacca actgctgtaa tggactgacc acggtcaatc caagaggcac
14581 aaccagttaa gactagcatg gccattgcca gtgataaaat cactgcaagg cctttttgttt
14641 ttttcatttc tatcctccga tatgttgtca caaaatttaa ccaatgcctt caatatatac
14701 tgttaaaaga cattaaatta attataccaa gtctaggtta attatgtcat gttacagttt
14761 gattacaggc ttcaaatatg gaatcttaaa cccttaggat agtagttttt taaagtattt
14821 ccaataacct tagcaaagcc aaatccatta ccatctacca aaagttgata ccaaccgttt
14881 tctaacgttt ggtcaagtgt aaccacatta ccagaggcat actgtgcaaa ttgttcttga
14941 gttaactcta tacttgagac tacttcatcc gatgttaaag caattccaag tgcaaaactt
15001 ggctcaaaac gtttttttctt gaagatgcca agatgcaagc cattgcgtgc gattttaact
15061 ttggaaagat ctggtagtcc cctaggaagt agatagaggt tgtcgccaaa gacttgtagt
15121 aaaccgtcta gaggtagctt gaggtgtttt ttaacaaaat catcccagag ctgtttttgc
15181 tccttattga ggtttgtttt cccctctttg atgcatgact gctcaggcat gcgcttatcc
15241 tggaattttg ccacaaactg accttccccc ttgaaatggt gtggatacat tcgagctgtt
15301 tctggaaaat caatcccatc gctcatcccg ttcaatttag ggattgccac caattcaagg
15361 tcagggtaat tctctaaaat ccaagcaaca acaccttcat tttcctcagg agaccaggta
15421 catgttgagt aaactaattg tcctcctggg gctaacatct tcaaaccatc tgctaggatg
15481 tccttctgca attgtgccag ttcactagga tagtccttgt gccaatactg gattgcatca
15541 gggtccttgc gaaacattcc ttctccagaa catggtccat caaagacaat catatcaaaa
15601 taatttttaa aaactttggc tagtttatca gctgacgtat tagtcacaac aacattttta
15661 gcaccaaatc gctcaatatt ctcaactaga accttgctac gtttgaaatt gatttcgttg
15721 ctgacgagta aacctgtgtt atccagatag cttaggagat gcgtggattt tccgcctggt
15781 gctgcggcta agtctagaac acggcttcct tttgatggag ctgccacttg agcaaccatt
15841 tgagcagctg gttcttgaga ataaactaaa ccagtcgcgt ggtcagaaga ttttcctgaa
15901 attttaccgt aatggcccca aggtgttcca ggaataggat caggatagtc cttctgctgc
15961 tttttaagag ggttaatgcg gtaggcagac acagcttctt gatcaaaaga tgtaaagaat
16021 tcctccgctt cagcaccaag taagtcttta tatttttcta taaaatctga tggaaatttc
16081 atagtttcct ttctatcaaa aaaactggac cactcaagag aacttgctat tctgatttgt
16141 ccagaatagt atatattttt aaagttaatt ttctgagtta agaatatctc tgatttcttc
16201 aagtctatct ttaggaacaa gcatcacagc ttggcgacta gaataatcaa gtagctctcc
16261 agtaatggtt tcaagttggt agcccaattt ttttcccata atacttgctg cagcataatc
16321 ccatgggtaa ataactgaaa agtaagctaa aatttgacca gaaaggacct tggacatact
16381 tagaccagca ccaccataca ctcgaacacc caaagttttg tcaatgaatc cttttatacc
16441 atggtaattt ttagcgtaca tagcagcatt actacctact agacaacgat ccagaggacg
16501 tctcttatat ggtgaaagca atttatcgtt gcagtatacg tcaaactgac ccccaccata
16561 gaaaagctga tcagccataa cgttgtatat taggccaaat tttccctgtc ccttttcgta
16621 ataggcaatc atgacacaga aattatcctg ttggacgata aaattgactg tcccatcaat
16681 aggatccaag acccagacgt ttccatcttt gatattatga accatgttat tttcttcagc
16741 caaaatattg tctgttggga aagctgattt aatgcgtgct actagtaaat cttgcgtttg
16801 tttatcaata ttagttacta aatcatcaaa agcagacttt tcctctaccg tgattgtttt
16861 actcaaactt tttttaataa acgccccagc ttccttgatg atttctttag caaaaacaag
16921 tttattttcc aagtgttaca aatccttttt cttttgcttt agcagctctg acagcttggt
16981 agattgaaaa accgcttgct tcttggaagg ccttgtctag ttgcttttca ggtgcttttc
17041 ctggaacaac tgttttaaac gccttataag cctccaagag ctgatcacgg tcaaccttac
17101 tctcatatgc tttttctacc tgactcaaaa aatgaagcac tgtggtaatt tcctcagtgc

17161 tccatgatgg atcgagtggg taggaataat ttcctttagt cattattacc cttaatttca
17221 gcactaatag tcgcttgacg caattcttgc tttctatagt cacgaggcaa aaaggcacga
17281 atctcatctt cgttaaaacc gatttgcata tgtctgttat ccataatgat tggtcgacga
17341 agaagatttg gatattcaga aatcaattga atgagctcct caattgtcaa attatccaca
17401 tcaatattca acttctgaaa gactttagaa cgagttgaaa tgatatcttc agtaccattt
17461 tccgtatgag acaaaatatt catcaattca tcgtgagtta atgggctcgt gatgatattg
17521 cgttcgtcaa aagcaacttc ataattgatc aaccatgctc gtgcctttct acagctggtg
17581 cagctaggtg atgaaaataa agttaccatg caaacacccc tttcacatta actttccaaa
17641 aatattatac attattttct tagttttggc tacgctttct taaagttttc cgcaattttt
17701 tttatccgct ttcagttggt cacatagctc gtctatatta ttgaatttta ttatatcacg
17761 gatataatct aaccagaaga cttcgattgt tttccatat atatcacctt caaaatcaaa
17821 gatattggct tcaagacgta actctgtacc accaaaagtg acgtttttttc ctagactagt
17881 catggaacga tagcgcttac catcaattac gacatccgca acatagacac catctgctgg
17941 tagaatagtc ctatcgatag gtgctagatt tgctgttggg aaaccaatag tccgaccacg
18001 cgcatcacca tgtaccacga tccctcgcgt agagaattca tgtcctaaga gattattggc
18061 ctctttcaca ttgccttctc ggattaattg acgaacacgc gttgatgaaa tcttttcgcc
18121 accactttgc tgttcctcaa tgacatgaac agtaccagaa aaattacggt ctaaatagtc
18181 cgtatcagtg cgatggtgtc cgaatttata gtcgaaccca actacaatct cttgagcttt
18241 tagacggtta atataacgtg taatgaagtc gtcagaactt gttcgagcaa aatcactatt
18301 aaaatctgtt aagtaaagtt gatctgtgcc ataggcttta aaaagatcac aacgtctctg
18361 aggtgctgtg atatgcagaa ggagatcatc cgtatagcgt tggaaagtca gttgagggct
18421 ctcgttaaag gtaaggacag ctacttcaag ctgtttttca tcagcaatct ggcgtgcctt
18481 atcaaaaagg accttgtgac cacgatgaag agcatcgaaa taccctaaga ccaagacggt
18541 ttccttatcc tgatggatat ctttataatc gtttatcggc gttactttca ttgacttaat
18601 acttttctag gtttatagac ttgacctcgc ttttccataa tagctagaac tttgtcgccc
18661 aaaaaggcag caaccaaagg ctcttggcta ttaattgaaa taaagcgacc aaatgagatt
18721 tctttggcat catcttcagt gacttgtacg gctggcaggt cctgaacgcc aaattcaata
18781 gggagtagga aggactggtc tcctgcttct accatttcag aaatttgtga cagagttagg
18841 gatgaatcta atgttaaacc tgcagaagct gttcgacgca gagcagacat atgactagca
18901 aagcctagtt ttacacccaa atcaacagat aaggtgcgga cataagttcc tttactacaa
18961 gcaacacgga aggtgaatct tgcagtaccg ttttctagct caataggtga ggttcggaca
19021 aactcggtaa tttttacctg acgttgagga cgttcaactt cttctccagc tcgggcgtac
19081 tcataaagtt tctttccgtt gatcttaacc gctgaataca taggaggaat ttgtgtgatt
19141 tctccctcaa aactagccat cgcctgatcg acagttgctc catctagctc tgttattgga
19201 gttgtttgga cgacttctcc actagcatct tcggtagttg tcgaaaatcc aatagtgatt
19261 tcgccttcat agactttacc cgcttcagtc atgtattcaa taacacgagt agcttttcca
19321 actgctatag gtagaacacc agtaacatct ggatcaagag ttccgccgtg cccaattttt
19381 tttctcatgt aatattttgc gtagtttaaa aacagcatca tgactggtca ttccagcctc
19441 tttttttag attaataatt cctgaaatca tacttttaat tatatcaaaa gcaatcctaa
19501 agagctaggt agaaacataa agaggcagag aacaacatct ctaacctcct aacttttata
19561 atgagaagaa atcacaggac tacttgttca ttttaatcga tatctctatt tagatcactt
19621 tccaattcaa agaaagtcta aatatcttgc acatactcta agacaccttg aaaatccccc
19681 tggtcatccc tgacagcagc ataagttaca tagacgaatt ttcctaaacg ttcagacttg
19741 aaccacattg gaacttggtc acgttcccca cttctgagta agttaaaggt tttcttgacc
19801 ttatcaagta ctttgggtgg atgacgaagt tcaacatttc gtccaatttg gctaggtgtt
19861 cgtttaaaga ccatctcatc cgccgggaca ttatcattgt agtattggaa aacatcatct
19921 ttattaacaa aagtgatttc caaagggaga tgattaagaa tgagattagc ctgttcaaca
19981 gaaaggtaac cattaccaaa tggctgagtg gtttctctgt ttgcgacaga tttatttttt
20041 ggttttggcg tgaaggtgat agtgaattga ccctcaggag tgtcgatgat tcgagtattt
20101 tcggtatttt cctcagtcct tgtcctagac tgtttttttt tataattgca tagccgtagg
20161 catcgctttc ctcggcaatt gaaagctaat catcctgtgt gaaggtttca agcagaatca
20221 taagtaagat agcctcttct tttaagatca tagcttcaaa ttcttccgaa aatgtttcga
20281 atgcttttga gaattcttca atatcagcat cagagttcga aagttcttgg agtttagacc
20341 ctgcatctca aaagagtttt cggatatcgt catctactct ccacataacc tttggaggtg
20401 aatcgtgacc ataacgctcc ataatgggaa agaaaagctt ttctttacgc gtataatgat
20461 tatggaattg cccaagtaaa gtcatctgat gcttaagacc gtttaataaa tcagatttaa
20521 agggctcatt ttcaggttta ctgatgtttt ctatgatacg acggatacga agtatagctg
20581 aacgtagggc tagattttct tacttgaaaa catagacagg atgaccttct tgatcagcat
20641 ctgctacttc gacatctgca attgctccct tgaataagtt ggcatggaca ttacagagac
20701 tcattacatc ttcaaaggta accccatctt cagatgccat caattcatgc tccatcatag
20761 atatttcaag tgcagatact cccttgaagt gttgattgaa aagctcttga acactttcgg

```
20821 gactagctcc atgatgtaaa tcaaggagaa tttctttaag tatttgaata cgattgtttt
20881 ccatattagt ctccgatgac ctcataacca ttgaacagta aggttcactt aattttagat
20941 aagggaatat ttgataactt agagccagct tttaaacttg ttaccttacc gacagtattt
21001 agcatagctg ggttggacaa gggcttgaaa ccaagattaa tcagtaattc cttgacctct
21061 ggatgctctt ttatgattgt tgcaacaggt tgtgataaat ctatagtgaa tgaaaaaaaa
21121 gaccaacaaa tcttagtttg ttggctttgt taattcccaa atagggcttc tgtaacgttt
21181 tgctggatat tttcccactc ctgctctgca cgaaccatct tacggtggta atcggcacgc
21241 ccacgtaagt aggcagcaat ttgataatct tgcttaacaa tcggtatctc aggaatttta
21301 atatccaaga ggtcagctgt cgacaaattt aggacagcct tacccttatc agcatagtct
21361 aaataggcac gcccaatctc agtttcaaga aagaatttga tgtagaaacc acgaagtttc
21421 tctttgggac gaagtactgt tatgtttgat gaagcgacta cttcacgctt accttgatct
21481 tcgaaaacag caactttctg aaccgttccc ttagaagcaa taaggacatc accatcttct
21541 aaaagaaagc gtaataattt tcggcgttct tcagaaaacg tctttaggtt atcgtaggca
21601 atgccgttgc ttgtcatatc cgataggtta ataactgcaa actctcctgg ttctgcctta
21661 gcaggaacgg cttttccttt aaattgatcg acaacatctc ctaaacgaac gagtttatct
21721 gtattagctt cgtaatcgat aataagttga ggtctttctt tttcagacat tggatctcct
21781 ttttatattc tagtatccgt aattagtatt tctatacaat aatagcaaga agctaatact
21841 tttgcaagtg aaaactgtaa taaaggcaaa aaaagagacc taagtctctt aattataaac
21901 ttttaacagc tgccactgct gcttcatagt ttggatctga attgatattg tccaaatact
21961 caacataggt aatcttgttg tcagcatctg taactaaaac tgcacgagct aacaagtgcc
22021 attcttcgat aaggacacca tattcttttc cgaaactatt gtcatagtag tcagaaagca
22081 tgatagcatt ttcaagacct tcagcaccac accaacgagc ttgtgcaaat ggtaggtcca
22141 ctgaaatagt aattaccact atatcatcta gttctgacaa ttccttgtta aaggtacgtg
22201 tttgagttga acagatacct gtatcaatgg atggaaggat agagataact tttttcttac
22261 cgtcgaattc tgaaagtgtt ttttgactca aatctgtggc tgtcaagtta aagtcacgca
22321 agatatctcc aacttgaagt tgcttgtctt cagctactgt aactggtttt ccaataaatg
22381 ttggcatgtt agtcctccaa tacttttttta actattgtac tgtaaatata gggacaagcc
22441 taatcatttg tttagaattt ttcaaacagt agtctaattt tttcagcaat aactgcatta
22501 attgcaggaa taacctcatc cgcaaagtta tttaggtagg gataatcacc aatttgcact
22561 ccatatatag catttttgaa aagagaaata tcattttgat catttccaaa agctacaaag
22621 tctttaccaa agtggtttaa aacagtacta gccttggtca cacctttagg attaatgtaa
22681 agacaccttt cgatatcatg ataactaagg tctaaacatt ccagtttgtt tagattaaag
22741 ataaggtctt cacgtatatc ttcatgctct acaagtggaa taacaacttt gatgggctca
22801 ttaagttcat gaataggcaa aatccttgct ttgtttaatg gatcaacatt tgaaagaaaa
22861 gggatttact cagaattttg aatagcataa ttaaaggtat catcaataaa ataagggaga
22921 ttataaatat cacagtagct aacaatcgtt cttagagcag aagaaggtat tctgtattgt
22981 gatactaagc gttcatcttg aaaaacttca ccaccgttta agctaactac tgtcagcttt
23041 ctcaattctc cttctaaaat actaagacag tctctgtaag agcatgctgt tataaaaata
23101 atttcatgcc cgtattttgg tgcagttaaa agaacctttt gtaggtcttt tgacatggtc
23161 ataccatcaa aacatagggt tccatccaaa tcaaaaacaa atttcatagg gatagtataa
23221 caaaaaacca actaaagagg gctctataat ttctgtagtg ggtaaaacta ctgtagagat
23281 tatagagctt tttgagtgta cacaaaaagt cccataagaa ctataatgaa aagcgactaa
23341 actaccatta ggaagaactt atgagactta ttaagaataa cacagaatta atcggaatca
23401 aagaccaaaa tatcaagatt tcacttgttt ttgaaactga cactcatatc gagattcaag
23461 caaaacttga ttaccctgca ccatcatgtc ctcattgcca tggaaagatg atcaagtacg
23521 actttcaaaa gccttctaaa atccctcttc tcgagcaagc gggaacgcca actctactac
23581 gtctgaaaaa acgtcgtttc cagtgtaaaa attgtaggag agtaagggtg gctgagacat
23641 ctatcgttga gaaaaactgc caaatctcca atctagtccg acaaaaagtt acacaactcc
23701 taactgagaa agtgtcacta actgatattg caagaagact tcgcgtgtcg acgtccactg
23761 tctatcgtaa gcttgatcag tttactttca aggaacatta tgacaaactc cctgctgtta
23821 tgtcctggga tgagtttggg ttcaagaaag gggaattgac ttttgtggcg cagaattatg
23881 agacaaacga actcataacc atccttgata atcgccgcca aactactata cgaaactact
23941 ttttgaagta tccattgaaa gtccgacaac aggtacagtt tatcacgatg gatatgtctg
24001 gagcttatat tccactggct cgcaagttct ttccaaacgc aaaaatcgtt cttgatcgtt
24061 tccacatcat ccagcacctt ggacgtgcct ttttaaagac aagaattgca attatgaacc
24121 agttcgataa gaagtcacta ccctatcgag ccctgaaaaa tcactggcga ctcttccaaa
24181 aggacagccg taaactatct ctcaactctt tttattcaaa gactttccgc caaactttag
24241 caccacatga agttgttgcg aagacactag tcttttcaaa agagcttact gactactata
24301 cactttatca gcttttgctt tttcactttc aggagaagag agtagatgag ttctttgacc
24361 tgatagagga aaataggagc aaggtcaatc gctattttca aactgtcttt aggacttttc
24421 ttagacacaa gcaatacatc aaaaacgcac tagaaacgga ctattcaaac gcaaaacttg
```

24481 aagcgactaa taagttgatc aaagacatca aacgtttggg cttcggtttt agaaacttta
24541 tcaactttaa gaagcgtgtc ttcatcacac tgaacataca taaaaagagg acctatccgg
24601 tcctctctag atgttagctt ttcgtcaccc actacagttg acaaagagcc ctaaagagct
24661 ggctaaatca ttttgtattg aaatacttag attttatcaa gagctagttt taaatcttca
24721 atcaaatcat ctgcgttttc caaaccaact gagatacgaa tttgatttgg agtgacacca
24781 caagctagta aatcagcttc tgccaattgt tggtgagttg ttgttgcagg atgcaccacc
24841 aaagatttg catcagcaac gttagctaaa tctgagaaga tttcaagatt atctataatg
24901 gtacgtgctt cgtcttgtcc acctttaaca tggaaagtga aaatagaacc tggtccttta
24961 gagaagtatt tttgtgccaa atcatagtaa ggacttgatg gtaatccagg atagttaact
25021 ttttcaactt ttggatgatt ttccaaaaag tctacgattt ttttagtatt ttcgacgtgg
25081 cgttcgacac gaagtgaaag tgtttcaagt ccttgcaaga gcaagaaagc attgaaaggt
25141 gccaaggcag cccctgtgtc acgcaaaagt tgtgtacgaa gggcagttac aaatgctgct
25201 ggaccaacat cacgcgtata gcttatattg tggtaagatg ggtcctcgtc aaccaactgt
25261 gggaatttgc ctgaagcttc ccagtcaaat ttaccagaat cgacgataag acctccaata
25321 gttgttccat gtccaccgat aaacttagtt gcagaatgaa ccacgatatc tacaccatgg
25381 gagataacat tgatgagata tggagttcca aaagtattgt cagcaaccaa cggaatctta
25441 tgactgtgag caatctcagc aattttttca aaatcaggaa tattgataag cggatttccc
25501 aaagactcaa tcaagacaag cttggtatta tctttgatag cagcttcaat ttcttcagga
25561 ttgtcaacat ctacaaaagt tgttgtgata ccatagcgtg gaagagtttc ttttaagaga
25621 ttgaatgtcc caccatagag ggttgttgcg gcaacaacat gatcgccagc atgtgccaaa
25681 ccaaggatag cataggtgat tgcagccata cctgatgccg tagccagggc cccaacacca
25741 ccttcgaggg ctgcaatacg ttcttcaagt actgctactg ttggattagt aatacgtgtg
25801 tagatattac caggctttct aagggcaaag agatcttcac cttcttgagc gtcgtcaaaa
25861 acatatgaag tggtttggta aattggaaca gcacgtgatt ttgtttcctt gtcaggtgtt
25921 tgtccagcgt gtaattgtaa ggtttcaaat gaaaattctc gtgacatatg agtgtcctcc
25981 aaaacaaaag ttgccttcat tctacaccta gaaaagctag ctgtctaata gaaatttttt
26041 atcaatgtga tagggagggc ttataaaaag gacctttcgg tccctttcta tttcttcaca
26101 ataggtgcta cgcttgccaa gactttttta agtccaactt ctggaaattt tatcttaagt
26161 tcctgtgtct tgccacttcc agttacttct aggacggtac catctcccca tttcttgtgg
26221 tgagcgatat ctccgatttc ccaatctgta gctgagtttc cagagtctga agccttacta
26281 aaaggtagtc cccagtagc ttttgaaaat ggctgagcgc caccagtatg tttttgcggc
26341 tgtgcatttg cttttctggt ctgaagcgct tgttgtaaac tcatgccctg accaaattgt
26401 atcggctctt cctttgtaaa acgaacacca aatgatgaat tggctggacg tgctagacct
26461 tgatattgta ataaatcatc tgaaatttca cgtaagaaac ggctaggacg attatagcca
26521 gtcttaccaa aaagcgtacg cgtattagcg ttggtaagga aaagaatctc ttccgcacga
26581 gtgataccta cgtaagccaa acgacgctct tcttcgagtt catctggctc ttctgaagca
26641 cgtgacaaag ggaagactcc ttcttccata ccaattagga agacaactgg gaattcaagc
26701 cctttggcgg cgtggagggt cattaaggtt acttctgctg cctcagcctc accatcgtcc
26761 gtgtcagcaa tcaaagccaa atcattcaag aaacggccta aacggtcaat gcctgtctca
26821 tcttcagttc catctgtatt agtctcgtcg aaattctttg ttacagacat gaactcttcg
26881 atattttcaa tgcgtgattg gctttcaagc gtttgttgca tggacagagc atccagataa
26941 ccagacttgt ctaaaatagc ttctacagta tcggtaatgc ttagcccatc caactgatca
27001 cgtaggttca gaatcatgtt agcgaaatcg taaacacctt gagctgcttt ccctttaata
27061 ggagacaaca tgatattagc tgaagcatct aggagactca tgttttgctc ataggcaaag
27121 ttccgaagct tttcaagtgt gccaggccca actccacgtt taggttcatt gacgacacgt
27181 tcaaaactaa tgttatcaga agtgttggca atcaagttca aataagaaat aacatcacga
27241 atctctttac ggctgtagaa ttttgtccca ccaaccattg tatatggaat attagacttg
27301 agcaaggctt cctcaatggt acgagactga gcattggtac ggtaaagaac agcgaagtct
27361 ttgaaattct tgactttttc acggacgata ttatcgattg ttgaagctac gaatacagct
27421 tcatcacgct cgtcgttagc acggtaataa acaatctgtt caccttcatc attttgagtc
27481 caaagtttct taggacggcg gttgcggttg ttcttgatga cttcgttagc cgcttgaaga
27541 attttcttgg ttgaacggta gttttcttcc agaaggacaa ccttagcttc aggataatcc
27601 ttctcaaaat caagaatgtt ctgcatatca gctccacgcc atccatagat tgattggtca
27661 gcatcaccca cgacacagat attcttaaag cgtgaagcta ggagttttac caattggtat
27721 tgagcatggt tggtatcttg atactcatct acgtgaatgt actgataacg ttgctgataa
27781 taagcgagga catcgggatt tttgtcaaag agacgaagag tcatcataat caaatcgtcg
27841 aaatccatgg cctcactacg acgaagttct tcttgatagg ccttgtaaca tttggctacg
27901 atttgcgtat acatgtcacc agcttgatgc tcataagcta tctcatctaa gaggtcattt
27961 ttggcgttag aaatggtccc taaaatggca cgttcattcc actttttagg atctaaattc
28021 agattcttga gaatacgttt catcaaagta cgttgttctc ctggatctac aatagtaaag
28081 tttctgttgt agccaatatg atccgcttca cgacgaagga ttcgtacaca catgctatgg

28141 aaggtcgcaa tcaaggtttc tgaagttgcg ggatttaaag ccactgcacg ttcacgcatt
28201 tcacgggcag ccttattggt aaaggtaatg gccaaaatat tccaaggatt gatcattttt
28261 tcgtcgataa ggtaggcaat gcgatgggta aggacacgag tcttacctga tccagcacca
28321 gccataatca aaagagggcc ttcagtcgtt tggactgctt cggcttgttg atcattcata
28381 ccagttaata atgggttcat catattctcc attctcaaaa ataagctcta ctattttatc
28441 atttttaga tgaaaatgca gagtctattc gtaatagaaa aaaataattg agtgaaaaac
28501 tgtttgtttt tgctagggtc tcagcttaat aaaaaggatg acattacgtg ctttttgaga
28561 gattgaaaac tatagataac gataaattga aagctcaata ttcggatttt ttacgtatat
28621 aataaaatta tatgttgaca gcgttcggtt ttttcgatat gatggaaccg taataaaata
28681 ataaccgagt gatgtcatca ggatgactgg tgattgacgg acttctggag agatcttgaa
28741 acggcttatg ctgtagacaa gacaccgaag ggcaaggga taagacccga aactctcagg
28801 taaaaggaca gaaagcattg aaagttttta actttcagta atagctttgt actttcagag
28861 gtctggttaa gccaaacctc tttttgacgt ctcggtctaa ggagatttta aacgcatgtt
28921 agacttttc acttccattg atgactttgt atggggacct ccccttcttg tccttcttgt
28981 aggaactggt atctacctta caatccgtct tggactttg caaatcattc gtctgcctaa
29041 agcctttaaa cttatctttg ctgaagataa aggagagggt gatatttcta gttttgcagc
29101 ccttgccaca gcacttgctg caactattgg tactggtaat attgttggtg ttgcgacagc
29161 cattaagact ggtgggcctg gtgctctttt ctggatgtgg attgctgctt tctttggtat
29221 ggcaaccaaa tatgctgaag gtctcttagc cattaaattc cgtacgcttg atgaccaagg
29281 aaatgtctct ggtggaccaa tgtattacat cacacacggt tttagtggaa aatggcaaat
29341 aatcgctaag cctttagcct acttcttcgc ttttgctggc gttcttgtcg catggttggg
29401 tattggtacc ttctcgcaag ttaactcaat cacatcatct cttcaaaaca gctttggttg
29461 gtcaccaaaa attgttagtg ttatcttagc tcttattact gctgctatca ttttcggtgg
29521 tattcaatct atttcaaaag tatccgaaaa agttgtgcca tttatggctg gtctttacat
29581 cattgccgca cttattgtca tttttgctaa tttcaatcag ctgctccctg tgcttgagat
29641 ggttttccaa tcagcattta ctggtaaagc tgctgtcgga ggttttgctg gagcaactgt
29701 catggctgct atgcgacttg gtattgcgcg tggggtattt tctaatgaat caggtcttgg
29761 ttctgcccct attgctgctg ctgctgccaa gactgaagag cctgttgaac aaggtttgat
29821 ttctatgaca ggtaccttca tcgatactat tatcatttgt acgcttactg gacttgctat
29881 tctcgtaact ggacaatggt ctggtagctt agaaggtgca ccaatgacac aagcggcctt
29941 ctcttctatt tttggaactt ttggagaact tgctctaaca ctttctttag tcttgtttgc
30001 ctttacgact attctaggtt ggtcatatta tggggaacgt tgtttcgaat ttattttcaa
30061 atctaccaaa ttcctctcat tttatcgtct tatctttgta gttatggtcg ctcttggtgg
30121 ctacctaaca cttgaggttg tttggaaaat tgcggatatc gtaaatggct tgatggcatt
30181 gcctaacttg attgccctcc tagtcctttc accaattatt ctcaaagagt ctaaatcata
30241 ttttgaacgt cataaataag gaaaagagct caggctcttt tttcttgcct ttaaaggtaa
30301 ccaatccttg ctactactgc tcatttgagt tacaatgaaa ttaaatcatt ttaaaacttg
30361 taaagaaaaa aatccaaagg aaaaactatg tcaattacca aagaattcga tactatcact
30421 gctatctcaa ctcccttagg tgaaggtgct atcggtatcg tccgcttgtc tgggaccgat
30481 gctattgcta ttgctaataa ggttttcaaa ggtaaaaatc ttgaaaccgt tgattctcat
30541 actatcaact acggccatat tgtcgaaaat aatgaaatta ttgatgaagt catggtcagt
30601 gttatgcgtg caccaaaaac ctttacacgt gaggatgtcg tagaaatcaa tacacatggt
30661 ggtgtcgcag ttaccaatga aatccttcaa ctcctgattc gttctggtgc tcgtatggcc
30721 gaaccagggg aatttaccaa acgtgccttc ttaaatggac ggatagattt gacccaggct
30781 gaagctgtca tggatttgat tcgtgccaaa actgataagg ctatgactgt tgctgtctct
30841 cagttagatg gttcgcttaa aaatctcatt aacaacacac gccaagaaat ccttaatact
30901 ttggcacaag tcgaagtcaa tattgattac ccagagtatg atgatgttga agaagttacc
30961 acaaatctag tccgtgagaa aactcaagag ttccaggctc tcttggaaaa tcttcttgca
31021 actgccaaac gaggaaaaat cctacgagaa ggtctttcga cggcaatcat aggccgtcct
31081 aatgtcggaa agtctagcct tctcaacaat ctcctacgtg aggagaaagc catcgtcact
31141 gatattgaag ggactacccg tgatgttatt gaagaatacg ttaatatcaa gggtgttcct
31201 cttaagttaa tcgataccgc tggtattcgt gacacagatg atgtggtcga aaaaattggt
31261 gttgagcgtt ctaaaaaagc tcttgaagag gctgatttgg tccttcttgt gcttaatagc
31321 tctgaaccac ttaccgacca agaccgtaca ctgcttgaca tcagccaaaa tagcaaccga
31381 atcattctct taaataagac ggaccttcca caagctatcc agactgaaga gcttcctgag
31441 gaccttatcc caatttcagt cctcaaaaat gaaaacattg acaaaatcga agatcgtatc
31501 aaccaactct tctttgataa tgctggctta gtagaaaaag atgctactta tctttcaaat
31561 gcacgccata tctcactcat tgaaaaggcg cttgaaagtc tcgaagccgt taatcaaggt
31621 cttgaattag gtatgcctgt tgacctatta caagttgata tgactcgcac atgggaaatt
31681 cttggcgaaa ttactggtga cgccgcaccg gatgagctta ttactcaact ctttagtcag
31741 ttctgtctcg gtaaatagac ataacttaat ttatgtaaaa agatagattg ctatcttttt

```
31801 attttaaatt aagttattac agaaatataa ttatgttaaa aataataaat ttatacctta
31861 aaatgatttt gccatgcttt tcgaaaaaga aaatgctgtc atggtactat cctatgattt
31921 tgaaaggtta atgatgggcc aacaaactac tgatactgat tttaaacatt ttatagattt
31981 tgatgataat cttctttttt agactaactg aaactaaagt tgacaatgga cgacctgaca
32041 ttctttttca ttggcaccca gaacttgaaa tctaatacgt ctatgaaggg actgctcgct
32101 atcacattga ttatgactat tttgatagtc aggctggaga tatctttcta attcgtccta
32161 atggactcaa ttctgtccat cctattgcta atcagtcgca ccatactaac actcttcatt
32221 ttcatttaga tatgttagga caatttctag tggatcctct tagtctccgt tatcttcaac
32281 ccttacagaa ttctaatttt aagttcaaac aatgtttaaa accaactgat gaggggtatt
32341 ctgaaattaa gggaacccta tttgcaattt ttaagatcat tagacaaaaa gagagacact
32401 acgggatttt gcttaagtcc aaatcggagg aactcatcta ccttttctac tactatcgtt
32461 tggtagagag aaagacttct gatgatcatt atcgtaaaaa tgaaaaaatt cgggaaatca
32521 ttgatcacat caataagcat tatgtcgaga cgcttactat tgaacaattg tccgagctca
32581 tggggtacag taagacgcat tttatgacca tatttaaaca acatacaggt acctcttgca
32641 ctgaatttat tattattcag gatagactga atgctgcttg tgaggaactc cgtaatagcg
32701 taaaaccagt tttagaaatt tctacgagcg ttggttttaa caacctttca aactttaacc
32761 gccaatttaa gcattattat gctcaaacac cgagccagta caggaagaca catagcaaca
32821 agaaaacaaa taagagttaa ccattggtta actcttatta tttatgcaga gaagctattt
32881 agtttttctc tttacctttg tgtttgccac taagtccaaa catcgtaagc aacatgccca
32941 gtccaagaag acctgcatgt gaagctttat cccctgttgt tggaagttct tcggtttggc
33001 ttggagtttc agtaactttt tcagtttcct taagagtttc gtttgaagtt taaacgaaga
33061 agactttacc gttaggtgtt gttactactt tagataccac tggtttcgtt ttttctgaaa
33121 ctttagaagc tacagttgag tcaacaggat ttactggatt aaccgttgta tcttctgcat
33181 tctttttctt gttcaaagca gcaaaattgg cacttgaaat agaaatttta cgtgaattag
33241 gattaataac tgcgtatttc gtaagatctg catgcgtcaa gtaatcggca aagactgtat
33301 tgagtgaagg accttcttca cgaagaccac caagcattgt gaagccatca ccaccagcaa
33361 gccaagaaat cgtttgttac tagataataa ggtgggatcc tctagagtcg acctgcaggc
33421 atgcaagctt gagtattcta tagtctcacc taaatagctt ggcgtaatca tggtcatagc
33481 tgtttcctgt g
//
```
